# Phase II trial with nivolumab and sorafenib in HCC identified enrichment of immunosuppressive monocytes in patients with Child-Pugh B liver dysfunction

**DOI:** 10.1016/j.jhepr.2026.101817

**Published:** 2026-03-12

**Authors:** Bridget P. Keenan, Zenghua Fan, Bryan Khuong Le, Quincy Harris, Jocelin Chen, Matthew Clark, Averey Lea, Li Zhang, Alexander Cheung, Frances Lara, John D. Gordan, Paige Bracci, Spencer C. Behr, Lawrence Fong, Alan P. Venook, Edward J. Kim, Robin K. Kelley

**Affiliations:** 1 Helen Diller Family Comprehensive Cancer Center, University of California, San Francisco (UCSF), San Francisco, CA, USA; 2 Division of Hematology/Oncology, UCSF, San Francisco, CA, USA; 3 Cancer Immunotherapy Program, UCSF, San Francisco, CA, USA; 4 Department of Epidemiology and Biostatistics, UCSF, San Francisco, CA, USA; 5 UC Davis Comprehensive Cancer Center, Sacramento, CA, USA; 6 Department of Radiology & Biomedical Imaging, UCSF, San Francisco, CA, USA; 7 Immunotherapy Integrated Research Center, Fred Hutchinson Cancer Center, Seattle, WA, USA

**Keywords:** Immunotherapy, Hepatocellular carcinoma, Child-Pugh score, Monocytes, Cirrhosis

## Abstract

**Background & Aims:**

Immune checkpoint inhibition (ICI) and anti-angiogenic therapies are active in hepatocellular carcinoma (HCC), although patients with impaired hepatic function have worse outcomes.

**Methods:**

We conducted an open-label phase II clinical trial to assess the safety and efficacy of the multikinase inhibitor, sorafenib, combined with nivolumab, in patients with advanced HCC and varying liver function. In a Part 1 safety lead-in, we investigated the maximum-tolerated dose (MTD) of the combination in patients with Child-Pugh A or B7. In Part 2, we enrolled patients with Child-Pugh B7-9 HCC with the primary endpoint of grade ≥3 treatment-related adverse event (TRAE) incidence. Exploratory endpoints included immunological biomarkers.

**Results:**

Overall, 25 patients were consented and 16 eligible patients enrolled. In Part 1, dose-limiting toxicity occurred in one of six patients in Dose Level -1, and two of five patients in Dose Level 1; Dose Level -1 was determined to be the MTD. In total, 69% of patients experienced a grade ≥3 TRAE, with similar distribution for patients with Child-Pugh A and B (70%, 95% CI: 0.35–0.93 *vs.* 66.7%, 95% CI: 0.22–0.96). The objective response rate was 6% and median overall survival was 12.99 months (Child-Pugh A: 15.26 and Child-Pugh B: 10.41). We found that patients with Child-Pugh B liver disease harbored more circulating suppressive CD14^+^ monocytes at baseline compared with those with Child-Pugh A disease.

**Conclusions:**

Although combination sorafenib and nivolumab demonstrated acceptable safety at the MTD in both Child-Pugh subgroups, the objective response rate was below the prespecified threshold to be declared worthy of further exploration. A distinct immune cell profile in patients with Child-Pugh B disease might define mechanisms of resistance and potential therapeutic targets in this population with unmet clinical need.

**Impact and implications:**

This study addresses the crucial need for effective therapies for patients with advanced HCC and compromised liver function, a population historically excluded from many clinical trials. The findings suggest that, although the combination of sorafenib and nivolumab demonstrated acceptable safety across different liver function subgroups, there was a low response rate overall. Importantly, the discovery of distinct immune profiles, particularly an increase in suppressive immune cells in patients with worse liver function, provides insights into potential mechanisms of resistance to immunotherapy. These results are crucial for understanding how we can improve the treatment of advanced HCC, especially in patients with impaired liver function.

**Clinical trial:**

NCT03439891.

## Highlights

• The combination of sorafenib and nivolumab showed acceptable safety in Child-Pugh A/B HCC.
• Grade ≥3 TRAEs occurred in 69% of enrolled patients.
• The objective response rate of 6% did not meet the threshold for further exploration.
• Patients with Child-Pugh B disease had more suppressive CD14^+^ monocytes at baseline.
• Myeloid cells should be targeted in advanced liver disease to overcome resistance.

## Graphical abstract

**Figure:**
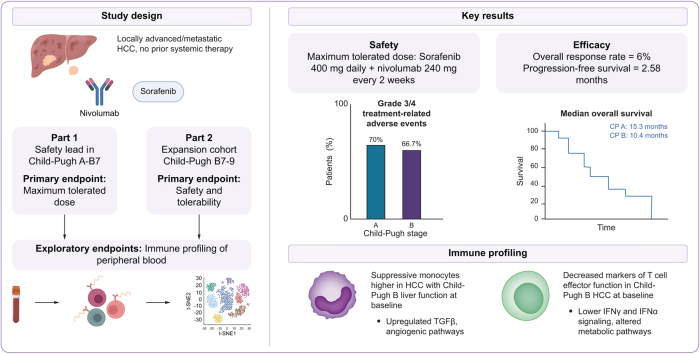

## Introduction

Hepatocellular carcinoma (HCC) is the third leading cause of cancer death worldwide and is increasing in incidence in the USA.[1], [2], [3] Most patients are diagnosed with, or progress to, advanced stages of disease requiring systemic therapies, although only a subset of patients experience durable benefit. Antiangiogenic therapies, including multikinase inhibition with agents such as sorafenib, lenvatinib, cabozantinib, and regorafenib, have been a mainstay of therapy for advanced HCC since the pivotal SHARP trial demonstrated improvement in overall survival (OS) with sorafenib compared with placebo.^4^ Immune checkpoint inhibition (ICI) with monoclonal antibodies targeting programmed cell death protein 1 (PD-1) and programmed cell death ligand 1 (PD-L1), such as nivolumab, pembrolizumab, atezolizumab, and durvalumab, can achieve durable objective responses in 15–18% of patients but low rates of disease control overall.[5], [6], [7], [8]

Combining antiangiogenic therapy with ICI holds the potential to augment the immune response through modulation of both the tumor immune microenvironment and microvasculature.[9], [10], [11] Multikinase inhibitors, the targets of which include vascular endothelial growth factor (VEGF) receptor isoforms, have been shown to differentially inhibit immunosuppressive immune cell populations, including regulatory T cells and myeloid cells, as well as inducing PD-L1 expression on immune cells, factors that might favor immune responses on ICI.[12], [13], [14], [15], [16], [17] Inhibition of VEGF or its receptors could also foster an immune responsive microenvironment through normalization of microvasculature.^18^^,^^19^

The benefit of combining ICI with VEGF-targeted therapies in HCC has been established in multiple phase III clinical trials.[20], [21], [22] The combination of atezolizumab plus bevacizumab was demonstrated to be superior to sorafenib in the IMbrave150 trial, with median OS of 19.2 months for atezolizumab plus bevacizumab *vs.* 13.4 months for sorafenib (hazard ratio [HR] 0.66).^20^^,^^23^ Likewise, the combination of VEGR receptor (VEGFR)-2 inhibition with rivoceranib and the anti-PD-1 ICI, camrelizumab, showed significant prolongation of OS and progression-free survival (PFS) in the CARES-310 trial, but was accompanied by higher rates of grade 3 and 4 toxicity.^21^ In the COSMIC-312 trial, a regimen of cabozantinib plus atezolizumab also achieved significant prolongation in PFS over standard sorafenib, but failed to improve OS, with higher rates of toxicity.^22^ Collectively, these studies validate the activity of ICI combined with antiangiogenic agents in HCC but underscore the potential for higher rates of toxicity.

Despite these recent advances in treatment options, an ongoing challenge remains to characterize the safety and efficacy of systemic therapies in patients with greater degrees of underlying liver dysfunction. Patients with more severe underlying liver dysfunction represent a substantial proportion of advanced HCC (40–50%) but historically have been excluded from prospective clinical trials because of poor prognosis.^24^^,^^25^ Retrospective data and small prospective studies suggest that patients with worse degrees of liver dysfunction, including patients with Child-Pugh scores of B or albumin–bilirubin (ALBI) grade 2 or higher, have lower rates of objective response and shorter median OS on systemic therapies, including ICI, compared with patients with preserved liver function, although rates of immune-related adverse events (IRAE) are similar.^5^^,^[26], [27], [28], [29], [30], [31], [32] Although there are poorer overall outcomes compared with Child-Pugh A, a subset of patients with Child-Pugh B cirrhosis achieve robust responses on ICI-based therapy that exceed the rate and duration of response for sorafenib monotherapy, underscoring the importance of identifying mechanisms of response and resistance in this vulnerable population with poor prognosis.^28^^,^[33], [34], [35], [36]

We developed this pilot study to examine the safety and preliminary efficacy of the combination of sorafenib with nivolumab in patients with advanced stages of HCC and varying degrees of liver dysfunction, along with exploring changes in peripheral blood immune cell profiles on treatment. At the inception of this study, there was no established first-line combination immunotherapy regimen for advanced HCC. With mid-study regulatory approvals of combination immunotherapy regimens in patients with preserved liver function, the focus of the study was amended to advanced HCC with Child-Pugh B liver dysfunction, an area of ongoing unmet clinical need. Beyond clinical endpoints of safety and preliminary efficacy, we explored blood samples using high-dimensional single cell transcriptomics and proteomics to understand immune responses to the treatment combination, along with the contributions of liver disease etiology and severity.

## Patients and methods

### Trial design

This was a single-arm, multicenter, investigator-initiated phase II trial with safety lead-in to evaluate the safety and efficacy of sorafenib and nivolumab for the treatment of patients with advanced unresectable HCC, without prior systemic therapy, with Child-Pugh A or B liver function. Participants were enrolled at two sites: University of California, San Francisco (UCSF) in San Francisco, CA, USA and University of California, Davis (UCD) in Davis, CA, USA. This study was conducted in accordance with the Declaration of Helsinki and after approval by the UCSF Cancer Center and UCD Institutional Review Boards. Both study sites were monitored by the UCSF Data and Safety Monitoring Committee (DSMC) according to the NCI-approved Data and Safety Monitoring Plan for multicenter trials. All patients provided written informed consent. The study was registered at Clinicaltrials.gov as NCT03439891. Part 1 of the pilot study was designed to first determine the maximum-tolerated dose (MTD) of sorafenib in combination with nivolumab in an Escalation Cohort with 3 + 3 design. Given the mid-study regulatory approvals of other combination immunotherapy regimens for patients with advanced HCC and Child-Pugh A liver function,^7^^,^^20^ the Part 2 Expansion Cohort of this study was amended to restrict eligibility to Child-Pugh B liver function to address the unmet clinical need in this population. The study was discontinued early in December 2022 because of the changing treatment landscape resulting in slow accrual.

### Patients

Patients with advanced or unresectable HCC,^37^ measurable by RECIST 1.1, who had not been treated with previous systemic therapy were eligible. In Part 1, patients with a Child-Pugh score of A or B7 were eligible; in Part 2, eligibility required a Child-Pugh score of B7–9 liver function. Patients were required to be age 18 years or older and have ECOG performance status of 0–1 at the time of enrollment. Patients with HBV infection were allowed with appropriate antiviral prophylaxis, but patients with both active HBV and HCV or HDV were excluded. Patients with uncontrolled ascites, hepatic encephalopathy within the past 6 months, or upper gastrointestinal bleeding within the past 12 months were excluded. Treatment with systemic steroids or other immunosuppression, active autoimmune disease, and uncontrolled high blood pressure (>140/90 mmHg at study entry) were also exclusions. Treatment with previous chemoembolization, radioembolization, local ablative therapies, or hepatic radiation was not allowed within 4 weeks of enrollment, and hepatic resection was not permitted within 6 weeks of enrollment.

### Treatment

In the Part 1 dose escalation portion of the study, patients were treated with sorafenib at a starting dose of 400 mg orally once daily (Dose Level -1) or 400 mg orally twice daily (Dose Level 1) plus nivolumab 240 mg intravenously every 2 weeks. In the Part 2 dose expansion portion of the study, patients were treated at the MTD, which was sorafenib 400 mg orally once daily and nivolumab 240 mg intravenously every 2 weeks. Treatment was continued until progression, unacceptable toxicity, withdrawal of consent, or the study end.

### Assessments

Adverse events were graded and recorded throughout the study according to NCI CTCAE Version 4.03. For Part 1 of the study, the dose-limiting toxicity (DLT) window was 28 days (1 cycle). Subjects had to receive two doses of nivolumab and at least 75% of sorafenib doses within 28 days (1 cycle), or experience a qualifying DLT event, to be evaluable for DLT. DLTs were defined as clinically significant toxicities that were at least possibly treatment related and met the criteria listed in the study protocol. Dose escalation was determined in a standard 3 + 3 design. Cross-sectional imaging with multiphase computed tomography (CT) or magnetic resonance imaging (MRI) of abdomen plus pelvis and CT scan of chest with/without or without contrast was performed at screening, then every 8 weeks until end of treatment. Tumor responses were measured using RECIST 1.1. The evaluable population for the secondary efficacy endpoints included all patients who had received at least one complete cycle of therapy (4 weeks) and had restaging imaging for response assessment or were removed for clinical progression.

### Endpoints

In the Part 1 safety lead-in, the primary endpoint was establishing the MTD of the combination of sorafenib and nivolumab in patients with Child-Pugh A–B7 liver function. In Part 2, the primary endpoint was the safety of the combination in patients with Child-Pugh B liver function. Secondary endpoints included the following: safety and tolerability of the combination overall; rate of IRAEs for the combination overall and in patients with Child-Pugh B liver function; objective response rate (ORR) by RECIST 1.1; and duration of response (DOR); PFS, and OS for the overall study and in patients with Child-Pugh B liver function. An ORR of 15% was prespecified as the target threshold for efficacy to consider further study of the combination.

### Research biospecimens

Peripheral blood mononuclear cells (PBMCs) were obtained from patients pre- and on-treatment for the subset of patients treated at the UCSF study site (Table 1; Table S1). Blood samples were processed using Ficoll (Cytiva, Marlborough, MA, USA); after centrifugation, the PBMC layer was isolated and cryopreserved in cell media with human serum and DMSO. Previously frozen PBMCs were thawed using media containing RPMI, heat-inactivated sterile filtered FBS, penicillin–streptomycin, non-essential amino acids, sodium pyruvate, and L-glutamine. For tissue correlation with PBMC findings, tumor samples were collected from a separate cohort of patients undergoing resection and consented under the UCSF Hepatobiliary Tissue Bank and Registry (IRB #12-09576) (n = 7 patients total; Table S2). For some resections, adjacent liver tissue was also available and processed in parallel. Samples were digested in RPMI containing Collagenase I & II (0.1 mg/ml, Sigma-Aldrich, St. Louis, MO, USA) and DNAse I, minced, and digested for 1 h using the GentleMACS system (Miltenyi Biotec, Bergisch Gladbach, Germany). Isolation of live cells (and, in some cases, CD45^+^ cells following sorting for live cells) was performed using MACS LS columns (Miltenyi Biotec).

**Table 1:** Demographic characteristics of patients enrolled in the Phase II clinical trial of combination sorafenib and nivolumab.

Demographic	Part and sorafenib starting DL			
	DL -1 (Part 1)	DL 1 (Part 1)	DL -1 (Part 2)	Overall
	n = 6	n = 5	n = 5	n = 16
Median age, years (SD)	64 (4.1)	67 (10.3)	66 (6.6)	66 (7.3)
Male, n (%)	5 (83.3)	3 (60)	5 (100)	13 (81.3)
Race, n (%)				
White	3 (50)	1 (20)	4 (80)	8 (50)
Black or African-American	1 (16.7)	0	0	1 (6.3)
Asian	2 (33.3)	4 (80)	1 (20)	7 (43.8)
American Indian or Alaska Native	0	0	0	0
Ethnicity				
Non-Hispanic/Latinx	6 (100)	4 (80)	3 (60)	13 (81.3)
Hispanic/Latinx	0	1 (20)	2 (40)	3 (18.8)
Viral status				
HBV+∗	3 (50)	4 (80)	2 (40)	9 (56.3)
HCV+∗	3 (50)	1 (20)	2 (40)	6 (37.5)
Non-viral	1 (16.7)	1 (20)	2 (40)	4 (25)
ECOG at enrollment				
0	5 (83.3)	1 (20)	2 (40)	8 (50)
1	1 (16.7)	4 (80)	3 (60)	8 (50)
Child-Pugh score at enrollment				
A5	4 (66.7)	2 (40)	0	6 (37.5)
A6	2 (33.3)	2 (40)	0	4 (25)
B7	0	1 (20)	3 (60)	4 (25)
B9	0	0	2 (40)	2 (12.5)
ALBI grade at enrollment				
1	2 (33.3)	0	0	2 (12.5)
2	4 (66.7)	4 (80)	2 (40)	10 (62.5)
3	0	1 (20)	3 (60)	4 (25)
BCLC				
B	1 (16.7)	1 (20)	2 (40)	4 (25)
C	5 (83.3)	4 (80)	3 (60)	12 (75)
Alpha-fetoprotein ≥400 ng/ml, n (%)	1 (16.7)	2 (40)	2 (40)	5 (31.3)
Extrahepatic disease	5 (83.3)	5 (100)	2 (40)	12 (75)
Vascular invasion	3 (50)	1 (20)	4 (80)	8 (50)

ALBI, albumin–bilirubin; BCLC, Barcelona Clinic Liver Cancer; DL, dose level.

∗ Some patients were both HBVcAb+ and HCV antibody positive, but were HBsAg negative.

### Single cell RNA sequencing/CITEseq

We then combined 1.1x10^5^ cells from each PBMC sample into staining buffer (1 mmol/L EDTA and 2% heat-inactivated FBS in calcium/magnesium-free Dulbecco PBS; Thermo Fisher Scientific, Waltham, MA, USA) and stained with one pooled cocktail containing a custom panel of 197 Total-Seq C antibody–oligonucleotide conjugates (BioLegend, San Diego, CA, USA) as per standard protocols (Supplementary CTAT Table) following pre-incubation with TruStain FcX (Fc Receptor Blocking Solution, BioLegend). Droplet-based single cell RNA sequencing (scRNAseq) was performed using the 10X Genomics Chromium Single Cell 5ʹ Reagent Kits v2, according to the manufacturer’s instructions. 10X Genomics 5′ v2 Library Construction kits for feature barcoding (protein antibodies) and TCR amplification were used to generate libraries as per the manufacturer’s instructions. scRNAseq of tumor samples was completed on fresh material with 10X 5’ v1 kits. All sequencing was performed on an Illumina NovaSeq S4 sequencer with paired end 200 bp read length and 25,000 reads per droplet for gene expression libraries and 5,000 reads per cell for feature barcode and TCR libraries.

### Bioinformatics

CellRanger (10X Genomics, Genome Build: GRCh38 3.0.0, Supplementary CTAT Table for all software versions) was used to align the raw sequencing data. The feature barcoding library sequences were aligned to a customized reference genome provided by BioLegend containing the oligonucleotide sequences corresponding to each antibody. Pooled samples were demultiplexed by a genotyping-free pipeline Freemuxlet to assign cells to each individual patient.^38^ Doublet detection was run by Scrublet^39^ using default parameters on each sample. We used the Scanpy^40^ data analysis pipeline for preprocessing and analysis of scRNAseq data. We applied the following cutoffs for filtering high quality cells: <10% mitochondrial genes, >100 and <2,500 genes expressed per cell, and excluded platelets, red blood cells, and doublets. We filtered out ribosomal genes and genes detected in fewer than three cells. Following sequencing alignment, preprocessing, quality control, and doublet removal, we recovered 177,000 cells from all samples combined, corresponding to >3,000 cells per sample. We log_2_ plus one transformed and normalized the data to 10,000 counts per cell, regressed out the percentage of mitochondrial genes and number of gene counts, and scaled genes to unit variance. We performed batch correction using Harmony^41^ and then performed k-nearest neighbor graph construction and clustering on gene expression data; for analysis of all immune cells, we clustered cells with a resolution of 1.0. We reclustered on myeloid or T cells individually, removing any contaminating cells (non-myeloid or non-T cell); for myeloid cells, we used a resolution of 0.6, whereas, for T cells, we used resolution 1.3. We processed protein data by normalization and log_2_ plus one transformation as for RNA. Differential gene expression analysis was performed by the package Memento,^42^ where significant genes (*p* <0.01) were selected for pathway enrichment analysis by GSEA implemented by the prerank module from package GSEApy^43^ and normalized enrichment scores <1 or >1 were selected for visualization. Similarity comparison of cell clusters between two datasets was performed by the pseudobulk method described previously,^44^ in which gene expression was aggregated from the cell level to the sample level. Shared genes in both datasets were selected for the correlation calculation, while, in each dataset, the union of top 100 genes in each cell cluster was selected.

### Statistical analysis

Part 1 of the study was designed as a safety lead-in. A standard 3 + 3 dose escalation was used to determine the MTD. In Part 2 of the study, patients with Child-Pugh B disease were enrolled at the MTD with the primary endpoint of safety as measured by rate of grade ≥3 TRAEs (CTCAE v. 4.03) assessed as at least possibly related to sorafenib, nivolumab, or the combination of therapies. An acceptable rate of grade ≥3 TRAEs was predefined as no more than 50% based upon the previously reported rate of grade ≥3 TRAE of 24.5% among patients with Child-Pugh B7–8 HCC treated with nivolumab in the CheckMate 040 Child-Pugh B cohort^33^ and 28% of patients with Child-Pugh B7–9 HCC treated with nivolumab in a retrospective institutional cohort,^26^ along with rates of grade 3–4 TRAE of 30–36% in patients with Child-Pugh B7–9 HCC treated with sorafenib in the GIDEON registry.^35^ With an expected sample size of 12 patients with Child-Pugh B7–9 HCC treated in Part 2, the 95% CI for determining the rate of grade ≥3 TRAE if the actual rate was 50% would be (0.22–0.78). With an abbreviated sample size of six patients in the Child-Pugh B cohort in Part 2, the 95% CI would be (0.10–0.90) if the actual rate was 50%.

To annotate cells, we used the embedded SCANPY function to identify differentially expressed genes in each cluster compared with the union of the rest of the clusters, which uses the Benjamini–Hochberg method^45^ to control the false discovery rate. For frequency proportions, weighted least squares was used to adjust for the number of cells sequenced in each individual and Benjamini–Hochberg method was used to adjust *p* values for multiple comparisons. To assess the correlations of the frequency of cell types with other cell types, ALBI score, Child-Pugh score, PFS, and IRAEs, we used Spearman’s rank correlation coefficient. For the IRAE correlation analysis, each patient was categorized by the highest grade IRAE if they had multiple occurrences.

## Results

### Study population

**Fig. 1:**
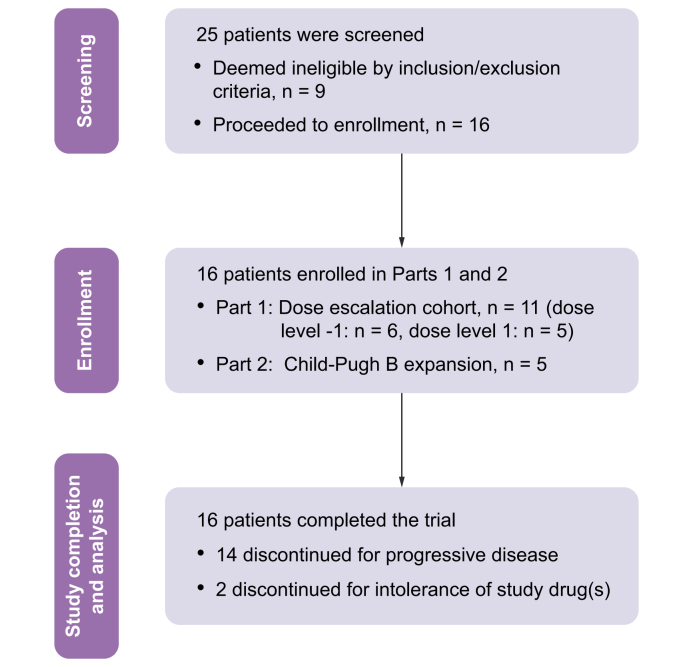
CONSORT diagram outlining trial design and number of patients in each part.

Between March 2018 and May 2022, a total of 25 patients were screened and 16 patients were enrolled at the two study sites: UCSF (n = 15), UC Davis (n = 1) (Table 1 and Fig. 1). Eleven patients were enrolled in Part 1, and 5 in Part 2 before study discontinuation. The median age was 66 years (range: 50–82 years), and 81% of patients were men. The study population was representative of the patient population seen at the enrolling sites and was 50% White, 43.8% Asian and 6.3% Black; 18.8% of patients identified as Hispanic/Latinx. Of the patients, 56.3% were HBV-positive, as defined by detectable HBsAg or HBV DNA and/or positive HBcAg, whereas 37.5% of patients had positive HCV antibody, and 25% had nonviral etiology. Liver function was characterized as a Child-Pugh score of A5 in 37.5%, A6 in 25%, B7 in 25%, and B9 in 12.5%, and ALBI grade 1 in 12.5%, 2 in 62.5%, and 3 in 25%. The Barcelona Clinic Liver Cancer (BCLC) stage was C in 75% and B in 25%. Other baseline patient and disease characteristics are summarized in Table 1.

### Safety

In Part 1, we assessed the primary endpoint of MTD of sorafenib in combination with nivolumab in DLT-evaluable patients (n = 11). At the starting dose of sorafenib 400 mg once daily in combination with nivolumab 240 mg intravenously every 2 weeks (Dose Level -1), there was one patient with a DLT (grade 3 rash) and, therefore, the cohort was expanded to three more patients who did not experience DLTs. The dose was escalated to Dose Level 1 (sorafenib 400 mg twice a day in combination with nivolumab); two out of five patients treated experienced DLTs (one with grade 3 hyperbilirubinemia and ascites and another with grade 3 fatigue). Dose reductions or delays were required in one (20%) patient in Dose Level 1, and four (36.4%) in Dose Level -1. The MTD was determined to be Dose Level -1, with sorafenib 400 mg daily and nivolumab 240 mg intravenously every 2 weeks. Part 2 patients were treated at the MTD with the combination from Part 1.

We next evaluated TRAEs that occurred in more than one patient by grade for Parts 1 and 2 combined (n = 16; Table 2), as well as according to dose level (Table S3) and Child-Pugh status (Table S4). The rate of occurrence of any grade 3 or 4 TRAE in Child-Pugh A was 70% (7/10 patients; 95% CI: 0.35–0.93) and in Child-Pugh B, 66.7% (4/6 patients; 95% CI: 0.22–0.96). The most common grade 3–4 TRAEs were palmar-plantar erythrodysesthesia, elevated aspartate aminotransferase, and elevated bilirubin, each occurring in 3/16 patients (18.8%). IRAEs possibly or definitely related to nivolumab were evaluated by grade for Parts 1 and 2 combined (n = 16) (Table S5). The rate of occurrence of any grade 3 or 4 IRAE in Child-Pugh A was 20% (2/10 patients; 95% CI: 0.03–0.56), and in Child-Pugh B, 50% (3/6 patients; 95% CI: 0.12–0.88) (Table S6). Systemic steroids were initiated for treatment of possible IRAEs in 38% (6/16) patients and in all but one case, the steroids were initiated after the first cycle of therapy was completed (Cycle 2 and beyond). Two patients with elevated transaminases initially attributed as IRAEs and treated with steroids were subsequently adjudicated as being more likely to result from disease progression with extensive tumor burden rather than being immune related. Another case of grade 3 rash treated with steroids was adjudicated as being more likely to be related to sorafenib than to nivolumab, based on response to dose reduction in sorafenib and enabling rechallenge with nivolumab after discontinuation of steroids. In total, three of 16 patients (19%) had confirmed IRAE requiring steroids, with one event each of grade 3 keratoacanthoma, grade 2 mucositis, and grade 4 immune-related hepatitis requiring prolonged courses of steroids. The patient with grade 4 immune-related hepatitis later developed grade 5 sepsis because of bacteremia.

**Table 2:** Treatment-related adverse events occurring in more than one patient by Grade.

Treatment-related adverse events occurring in n >1 patient, n (%)	Grade 1 or 2	Grade 3 or 4	Any grade
Rash	10 (62.5)	1 (6.3)	11 (68.8)
Increased aspartate aminotransferase	4 (36.4)	3 (18.8)	7 (43.8)
Diarrhea	6 (37.5)	0	6 (37.5)
Hypertension	5 (31.3)	1 (6.3)	6 (37.5)
Increased alanine aminotransferase	5 (31.3)	0	5 (31.3)
Myalgia	5 (31.3)	0	5 (31.3)
Palmar-plantar erythrodysesthesia syndrome	2 (12.5)	3 (18.8)	5 (31.3)
Dry skin	4 (36.4)	0	4 (36.4)
Hoarseness	4 (36.4)	0	4 (36.4)
Mucositis	3 (18.8)	1 (6.3)	4 (36.4)
Constipation	3 (18.8)	0	3 (18.8)
Decreased appetite/anorexia	3 (18.8)	0	3 (18.8)
Emesis	3 (18.8)	0	3 (18.8)
Fatigue	2 (12.5)	1 (6.3)	3 (18.8)
Hyperbilirubinemia	0	3 (18.8)	3 (18.8)
Nausea	3 (18.8)	0	3 (18.8)
Pruritus	3 (18.8)	0	3 (18.8)
Dyspepsia	2 (12.5)	0	2 (12.5)
Dry eye	2 (12.5)	0	2 (12.5)
Headache	2 (12.5)	0	2 (12.5)
Hypoalbuminemia	2 (12.5)	0	2 (12.5)
Hyponatremia	1 (6.3)	1 (6.3)	2 (12.5)
Weight loss	2 (12.5)	0	2 (12.5)

### Efficacy

**Fig. 2:**
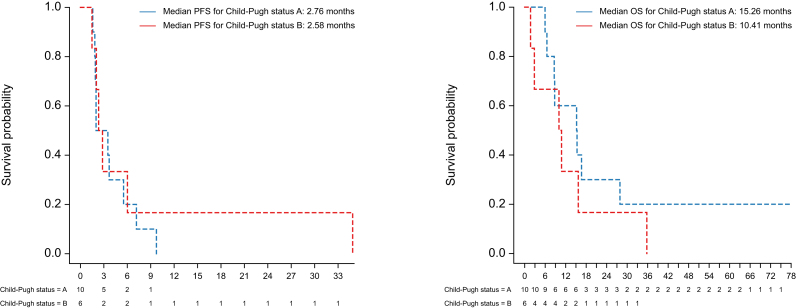
PFS and OS by Child-Pugh liver function status. (A) PFS in months for patients with Child-Pugh A (n = 10) and B (n = 6) liver disease. (B) OS in months for patients with Child-Pugh A (n = 10) and B (n = 6) liver disease. OS, overall survival; PFS, progression-free survival.

We evaluated the efficacy of the treatment combination across both dose levels due to the small sample size. Patients received a median of 4 cycles, with a range of 2-28 cycles administered. The ORR per RECIST 1.1 was 6.3%, with one patient with Child-Pugh A liver disease experiencing a partial response (PR) and no patients with Child-Pugh B liver disease with objective responses (Table 3). The disease control rate was 56.3% (nine of 16 patients), with four patients each with Child-Pugh A and B liver disease experiencing stable disease in addition to the patient with PR. For the one PR, the DOR was 9.73 months. Overall, the median PFS was 2.58 months (95% CI: 2.01–7.17), compared with 2.76 (95% CI: 1.91–NR) for Child-Pugh A and 2.58 (95% CI: 2.04–NR) for Child-Pugh B (Fig. 2A). The median OS was 12.99 months (95% CI: 8.65–35.8), compared with 15.26 (95% CI: 8.65–NR) for Child-Pugh A and 10.41 (95% CI: 2.83–NR) for Child-Pugh B (Fig. 2B). When clinical responses were analyzed by dose of sorafenib, patients treated with Dose Level -1 had longer PFS compared with patients in Dose Level 1 (3.68, 95% CI: 2.83–NR *vs.* 1.84, 95% CI: 1.58–NR months, *p* <0.001), and there was no significant difference in median OS (15.2, 95% CI: 10.03–NR *vs.* 8.78, 95% CI: 5.95–NR months; *p* = 0.932) (Table S7).

**Table 3:** Clinical responses overall and by Child-Pugh status.

Characteristic	Child-Pugh A (n = 10)	Child-Pugh B (n = 6)	Total (n = 16)
CR	0	0	0
PR	1	0	1
SD	4	4	8
PD	5	2	7
NE	0	0	0
Response rate, n (%)			
ORR (CR/PR)	1 (10)	0	1 (6.3)
Disease control rate (CR/PR/SD)	5 (50)	4 (66.7)	9 (56.3)
Kaplan-Meier estimate of median duration of SD, months (95% CI)			
Progression-free survival	2.76 (1.91–NR)	2.58 (2.04–NR)	2.58 (2.01–7.17)
Overall survival	15.26 (8.65–NR)	10.41 (2.83–NR)	12.99 (8.65–35.8)

CR, complete response; NE, not evaluable; NR, non-response; ORR, objective response rate; PD, progressive disease; PR, partial response; SD, stable disease.

### Correlative analysis

We explored the circulating immune response both at baseline and during combination treatment with sorafenib and nivolumab by performing scRNAseq with simultaneous surface proteome analysis (cellular indexing of transcriptomes and epitopes by sequencing; CITEseq) on PBMCs. For analysis of baseline immune profiles according to liver function status, we included five patients who had consented to the clinical trial but did not receive protocol therapy due to screen failure (Table S1) and one patient who was treated but only had PBMC samples available from baseline for a total baseline PBMC sample size of 20. For on-treatment immune responses, correlates of clinical benefit, and longitudinal sample analysis, we only evaluated samples from patients who had PBMCs available from at least the first two timepoints (n = 14). For purposes of the correlative data, given the small number of objective responses and recognizing that some patients who had prolonged disease stabilization with the combination regimen had clinical benefit, we defined disease control (‘DC’) as either a PR or stable disease with PFS of 6 months or longer (PFS6) (n = 2). Non-response (‘NR’) was defined as patients who had progressive disease as the best response or stable disease but did not meet PFS6 (n = 12).

**Fig. 3:**
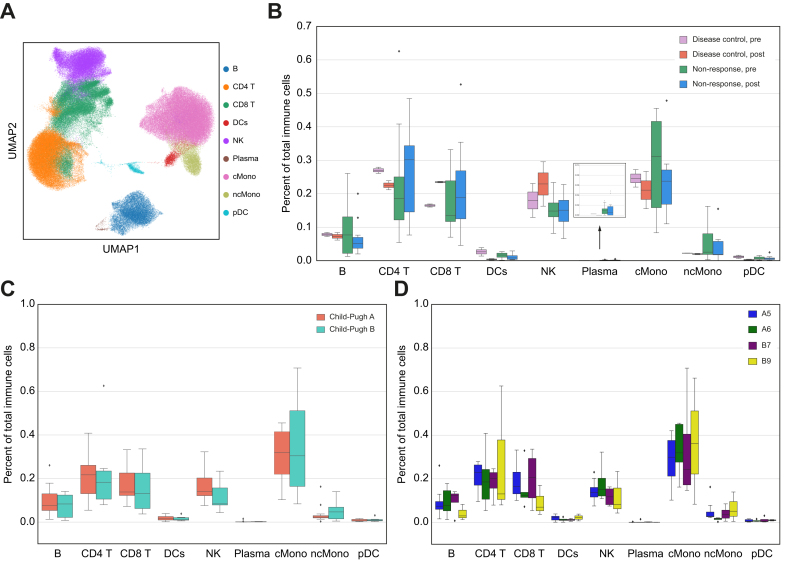
Circulating immune cell responses at baseline and following sorafenib/nivolumab. (A) UMAP plot of PBMCs colored by cell type, where B=B cells, CD4 T = CD4^+^ T cells, CD8 T = CD8^+^ T cells, and DCs = conventional dendritic cells. (B) Percentage of each cell type for patients with disease control (n = 2) or non-response (n = 12) before treatment (pre) and following one cycle of combination treatment (post). (C,D) Frequency of immune cell type at baseline (pre) for all patients (n = 20) by (C) Child-Pugh status (A [n = 13] *vs.* B [n = 7)]) and (D) Child-Pugh subscore (A5: n = 8, A6: n = 5, B7: n = 4, B9: n = 3). Boxes denote IQR, whereas bars denote 25% - 1.5xIQR and 75% + 1.5xIQR. Weighted least squares was used to account for different cell number per sample; multiple test adjustment with Benjamini-Hochberg for *p*-values. cMono, CD14^+^ classical monocytes; ncMono, CD16^+^ non-classical monocytes; NK, natural killer cells; plasma, plasma cells; PBMC, peripheral blood mononuclear cells; pDC, plasmacytoid dendritic cells; UMAP, Uniform Manifold Approximation and Projection.

Using the combined CITEseq protein and gene expression information, we identified the main immune cell types within the circulation of patients (Fig. 3A, Fig. S1A–C). We focused on immune changes 2–4 weeks after the first dose of combination therapy, based on previous studies suggesting that pharmacodynamic effects on immune cell profiles are evident within this timeframe^46^^,^^47^ and based upon the prevalence of evaluable samples at this timepoint. Comparing from the baseline timepoint before treatment (‘pre’) to following 2–5 weeks of treatment (‘post’), we examined changes in the frequency of circulating immune cell types (Fig. 3B). Although no formal statistical analysis was possible because of the small number of patients with DC, we observed that the patients with DC had an increase in CD8^+^ T cells and natural killer (NK) cells, and decreased CD4^+^ T cells, dendritic cells (both conventional [cDC] and plasmacytoid [pDC] dendritic cells), and classical monocytes following sorafenib/nivolumab treatment (effect sizes: 0.6, 1.11, 1.35, 1.54, and 1.84, respectively), whereas patients with NR appeared to have more static immune cell frequencies over time.

We next evaluated the immune response by liver disease status and etiology because these clinical factors have both been previously associated with the response to immunotherapy.^28^^,^^42^ Comparing at the baseline timepoint, there were no differences in the circulating immune cell frequencies between virally associated (n = 14) and non-viral (n = 6) HCC (Fig. S1D). Furthermore, within subgroups of virally associated HCC, there were no differences between HCV *vs.* HBV, HBV *vs.* non-viral, or HCV *vs.* non-viral, although this was an exploratory analysis because of the small number of patients with each disease association (Fig. S1E). There were also no differences in the proportions of general types of circulating immune cell at baseline by stage of liver disease (Fig. 3C,D).

We previously identified a population of novel circulating CD14^+^ monocytes with increased markers of chemotaxis (‘CD14_CTX_’) that could induce CD4^+^ T cell paralysis and was associated with ICI resistance in biliary tract cancer (BTC), another primary liver cancer,^46^ and hypothesized that these might also exist within the circulation of patients with HCC. In PBMC samples from patients enrolled in the current study, we subclustered on myeloid cells and identified CD14_CTX_ as well as other populations found in BTC (CD14_APC_: CD14^+^ monocytes characterized by antigen processing and presentation machinery and associated with ICI response; CD14_IFL:_ inflammatory CD14^+^ monocytes more frequently observed in healthy donors than patients with BTC; and CD14_ISG:_ CD14^+^ monocytes with an interferon response gene signature), as well as cDC, pDC, and non-classical CD16^+^ monocytes (Fig. S2A–D). We also identified a population of DC-like CD14^+^ monocytes that had gene signatures overlapping those of cDCs but expressed CD14, not previously identified in the peripheral blood of patients with BTC. Infrequent granulocytes and megakaryocytes were seen because of the processing of blood samples, which resulted in the removal of most granulocytic cells.

**Fig. 4:**
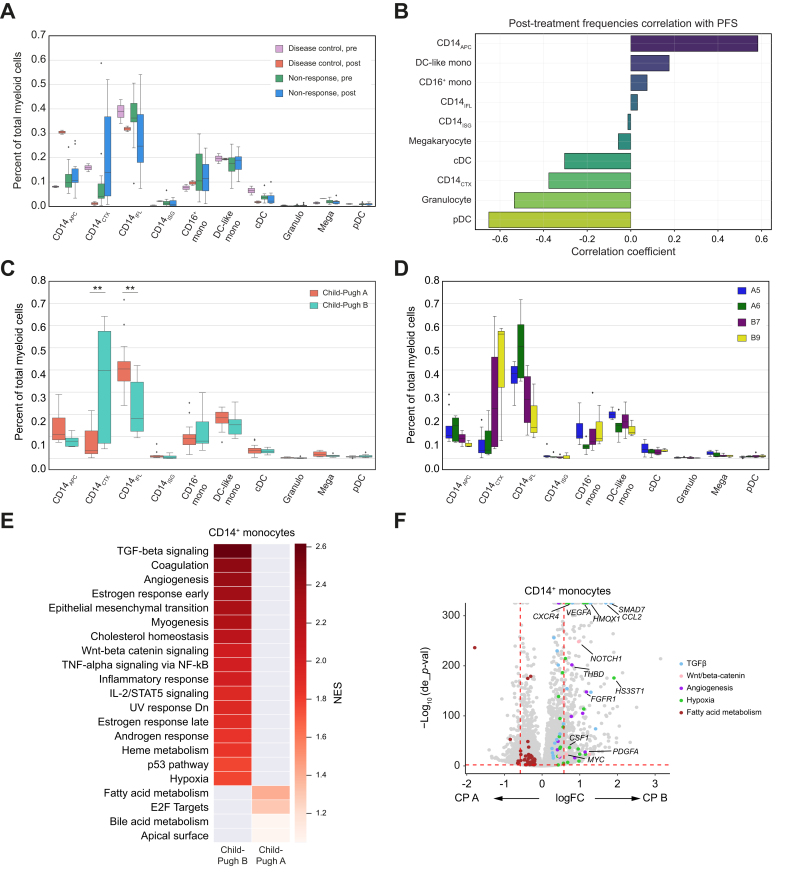
Circulating myeloid cells at baseline and following sorafenib/nivolumab. (A) Percentage of each cell type out of total myeloid cells in circulation for patients with disease control (n = 2) or non-response (n = 12) before treatment (pre) and following one cycle of combination treatment (post). (B) Correlation coefficients for the frequency of each myeloid cell subtype at the post-treatment timepoint and PFS in days. (C,D) Frequency of myeloid cell type out of total circulating myeloid cells at baseline (pre) for all patients (n = 20) by (C) Child-Pugh status (A [n = 13] *vs.* B (n = 7) and (D) Child-Pugh subscore (A5: n = 8, A6: n = 5, B7: n = 4, B9: n = 3). ∗Adjusted *p* <0.05, ∗∗adjusted *p* <0.01. Boxes denote IQR, whereas bars denote 25% - 1.5xIQR and 75% + 1.5xIQR. (E) Pathways enriched within all CD14^+^ monocytes (CD14_ISG_, CD14_CTX_, CD14_APC_, CD14_IFL_, and DC-like monocytes) in the Child-Pugh B compared with Child-Pugh A groups visualized for the top enriched pathways; heatmap corresponds to NES. (F) Differentially expressed genes in Child-Pugh B compared with Child-Pugh A disease, with genes of interest in the indicated pathways highlighted with the corresponding color dot. Weighted least squares was used to account for different cell number per sample; multiple testing adjustment with Benjamini-Hochberg for *p*-values. DC, dendritic cell; NES, normalized enrichment score; PFS, progression-free survival.

The frequencies of myeloid cell states appeared static with treatment in the NR group, whereas patients in the DC group had increased CD14_APC_ and CD14_ISG,_ and decreased CD14_CTX_ with treatment (effect sizes: 7.15, 2.69, and 1.3, respectively), resulting in a different myeloid cell composition compared with NR at the post-treatment timepoint (Fig. 4A). Acknowledging the small number of patients with DC, we also analyzed these findings by PFS status, which could be measured in a continuous fashion, performing a correlation analysis of the frequency of each cell population at the post-treatment timepoint with the length of PFS in months. Although this was also an exploratory analysis, we found a positive correlation of PFS with CD14_APC_ and a negative correlation with pDC, granulocytes, and CD14_CTX_ (Fig. 4B). Analysis of differential gene expression by response status revealed that the CD14^+^ monocytes in the DC group but not the NR group specifically upregulated IFNγ, IFNα, fatty acid metabolism, and glycolysis pathways, which might support productive immune responses, whereas the NR group upregulated IL-6/JAK/STAT3 signaling and oxidative phosphorylation, pathways characteristic of suppressive myeloid cell responses (Fig. S2E and F), and generally had fewer genes significantly modulated by combination treatment compared with the DC group (Fig. S2E).

In baseline blood samples from patients with Child-Pugh B liver function, there was a higher proportion of CD14_CTX_, compared with Child-Pugh A (Fig. 4C). Notably, a myeloid subpopulation seen more frequently in healthy donors than in patients with BTC,^46^ CD14_IFL_, was higher in patients with HCC and Child-Pugh A liver function, suggesting that, in Child-Pugh A, circulating monocytes retain normal function (Fig. 4C). Evaluating the frequency of CD14_CTX_ by Child-Pugh subscore (*i.e.* A5, A6, B7, or B9) before treatment (Fig. 4D) demonstrated a positive correlation between higher scores and the frequency of CD14_CTX_ pretreatment (R = 0.56, *p* = 0.011), whereas there was a non-significant inverse correlation between the numeric Child-Pugh score and the frequency of CD14_IFL_ (R = -0.4, *p* = 0.082).

We also evaluated a correlation by ALBI score (in which an increased score correlates with rising bilirubin, lower albumin, higher grade liver dysfunction, and worse prognosis^48^), finding a negative correlation with CD14_IFL_ (R = -0.49, *p* = 0.027) and a non-significant positive correlation with CD14_CTX_ (R = 0.34, *p* = 0.14), demonstrating that other measures of worsening liver disease correspond with a suppressive myeloid state. CD14^+^ monocytes from patients with Child-Pugh B liver function had upregulation of genes involved in TGFβ and Wnt/β-catenin signaling, angiogenesis, epithelial–mesenchymal transition, and hypoxia (Fig. 4E,F), consistent with the higher frequency of suppressive CD14^+^ states within Child-Pugh B compared with Child-Pugh A. Given the marked differences in myeloid cells by liver function score, we evaluated dynamics in myeloid cell frequencies with treatment within each Child-Pugh subset and found a trend toward increasing CD14_IFL_ and CD14_APC_ and decreasing CD14_CTX_ following combination treatment, suggesting that there could be partial reversal of suppressive myeloid phenotype with this treatment (Fig. S2G). By comparison, the frequency of CD14_CTX_ remained low in patients with Child-Pugh A liver function before and following treatment. There were no apparent differences in myeloid subpopulations between viral *vs.* nonviral HCC, or between HBV and HCV when compared individually (Fig. S2H and I).

Given the potential clinical significance of the myeloid cell populations in the blood, we evaluated for the presence of analogous myeloid cells within the tumor microenvironment of HCC. In prior parallel analyses in BTC, we previously found a macrophage correlate of the circulating CD14_CTX_ population in biliary tract tumors which expressed high SPP1.^46^ We performed scRNAseq on primary HCC tumors from resection specimens (n = 7, Table S2) obtained from a separate cohort of patients, some of which included adjacent liver tissue, and categorized the myeloid cell sub-populations (Fig. S3A–C). Intriguingly, we found many monocytic/macrophage populations that highly correlated with monocyte sub-types in the periphery (DC-like mono, CD14_APC_-like, CD14_IFL-_like, and CD14_ISG_-like), as well as dendritic cells, myeloid-derived suppressor cells (MDSC) and Kupffer cells. There were several tumor-associated macrophage populations (SPP1-Mac1-5); SPP1-Mac4 had both the highest expression of SPP1 and correlation with CD14_CTX_, and was largely found in tumor rather than adjacent liver tissue (Fig. S3D).

**Fig. 5:**
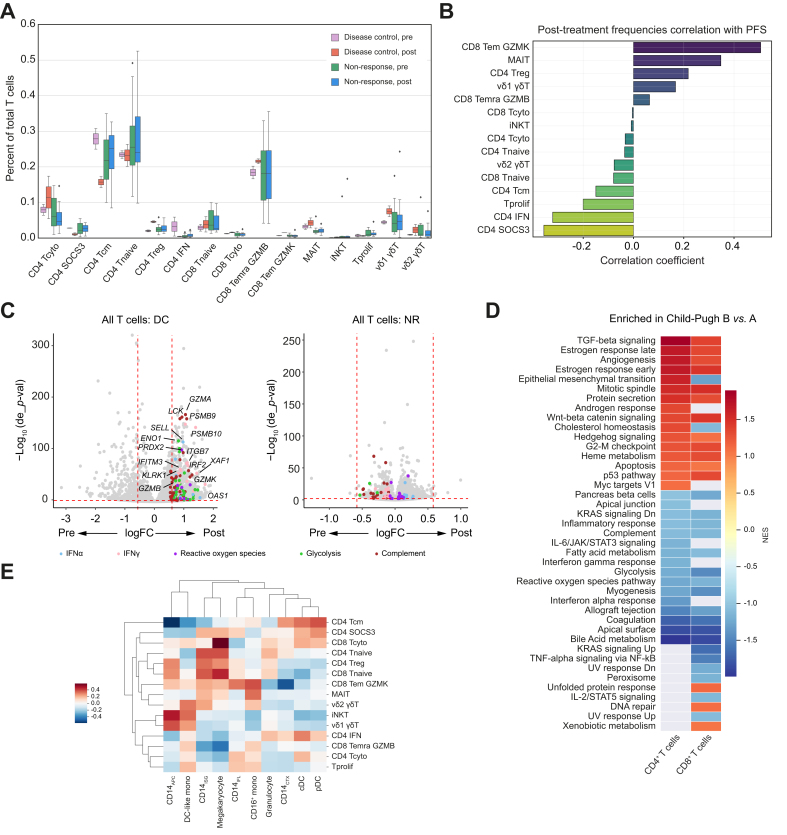
Circulating T cells at baseline and following sorafenib/nivolumab. (A) Percentage of each cell type out of total T cells in circulation for patients with disease control (n = 2) or non-response (n = 12) before treatment (pre) and following one cycle of combination treatment (post). (B) Correlation coefficients for the frequency of each T cell subtype at the post-treatment timepoint and PFS in days. (C) Differentially expressed genes in post-treatment *vs.* pretreatment timepoints for all T cells in the DC and NR groups are plotted, with genes of interest in the indicated pathways highlighted with the corresponding color dot. (D) Pathways enriched within all CD4^+^ or CD8^+^ T cells in the Child-Pugh B compared with Child-Pugh A groups visualized for the top enriched pathways; heatmap corresponds to NES. Positive NES scores are pathways enriched in Child-Pugh B and negative scores are those enriched within Child-Pugh A. **(E)** Correlation of T cell (y-axis) and myeloid cell (x-axis) frequencies across the entire patient cohort. Legend shows correlation values demonstrated in heatmap format. Weighted least squares was used to account for different cell number per sample; multiple testing adjustment with Benjamini-Hochberg for *p*-values. DC, dendritic cell; NES, normalized enrichment scores; NR, non-response; PFS, progression-free survival; Tcm, central memory; Tcyto, cytotoxic T cells; Tem, effector memory; Temra, terminally differentiated re-expressing CD45RA effector memory; Tprolif, proliferating T cells.

We next evaluated T cell subtypes beyond CD4^+^ and CD8^+^ T cells to determine whether there was a relationship between clinical factors and T cell populations, and between myeloid cell and T cell states. We identified 15 subpopulations of T cells, including several populations of CD4^+^ and CD8^+^ proliferating, effector, naïve, memory, and regulatory T (Treg) cells, and innate-like T cell populations, including γδT cells, mucosal-associated invariant T (MAIT) cells, and iNKT cells (Fig. S4A–C). We also specifically identified a CD4^+^ naïve-like population with high SOCS3 expression (CD4 SOCS3) and a CD4^+^ T cell population with high expression of IFN response genes (CD4 IFN) as we had found in BTC. As with the myeloid cells, we first examined whether there was a treatment effect on T cell subpopulations and found, in an exploratory analysis, that effector T cells, including a CD8^+^ Granzyme K^+^ T effector memory (CD45RA^–^CCR7^–^) subpopulation (CD8 Tem GZMK), CD8^+^ Granzyme B^+^ T terminally differentiated effector memory re-expressing CD45RA cells (CD45RA^+^CCR7^−^) (CD8 Temra GZMB), and MAIT cells increased with treatment in the DC group compared with the NR group, as did Tregs (effect sizes 2.57, 0.92, 1.44, and 2.95, respectively) (Fig. 5A). Within the DC group, there was a decrease in CD4^+^ central memory T cells (CD4 Tcm) and CD4 SOCS3 T cells (effect sizes 4.01 and 1.56, respectively). These findings aligned with the correlation analysis of PFS and cell frequencies, which showed a positive correlation with survival and CD8 Tem GZMK and MAIT cells and a negative correlation for survival and CD4 SOCS3 (Fig. 5B). Across all T cells in patients with DC, there was an upregulation of pathways and genes related to IFNα, IFNγ, reactive oxygen species, complement, and glycolysis, whereas in the NR group, there were few genes modulated with treatment (Fig. 5C). There were no apparent differences in T cell subtype frequency by viral status or Child-Pugh score (Fig. S4D–G). However, investigating gene expression profiles and pathway analysis revealed that CD4^+^ and CD8^+^ T cells in Child-Pugh B compared with Child-Pugh A had enhanced TGFβ signaling, angiogenesis, and Wnt/β-catenin signaling, and downregulated IFNα, IFNγ, and glycolysis pathways (Fig. 5D).

Finally, given the intriguing findings of myeloid and T cell dynamics induced by sorafenib and nivolumab, we evaluated correlations of cell frequencies between T cells and myeloid cells (Fig. 5E). CD8 Tem GZMK, the effector CD8^+^ T cell population that was increased in patients with DC and improved PFS, positively correlated with more favorable CD14^+^ populations, including significant direct correlations with CD14_IFL_ (R = 0.4, *p* = 0.008), CD14_APC_ (R = 0.2, *p* = 0.021), and CD14_ISG_ (R = 0.39, *p* = 0.01), and was inversely correlated with CD14_CTX_ (R = -0.52, *p* = 0.0004). Furthermore, pathways increased in T cells following combination immunotherapy were consistent with those seen in CD14^+^ monocytes in cases with DC (IFNα, IFNγ, and glycolysis), suggesting that there is crosstalk between myeloid and T cell populations.

IRAEs have been correlated with ICI responses and survival in patients with cancer.^49^^,^^50^ To test for a relationship in our cohort, we explored whether there was an immune signature characteristic of more severe IRAEs. We analyzed PBMCs at both the pretreatment timepoint for populations that might predict IRAE development, as well as on-treatment cell frequencies, which might indicate those immune cells involved in the emergence of IRAEs. The most highly correlated myeloid populations with IRAE severity were CD14_CTX_ and pDC at baseline and granulocytes post treatment. Perhaps counterintuitively, myeloid subtypes capable of antigen presentation, including CD14_APC_ (pretreatment), cDC (post treatment), and DC-like monocytes (pre- and post treatment) were negatively correlated with IRAE grade (Fig. S5A). We also evaluated whether there was a T cell signature that was characteristic of severe IRAEs (Fig. S5B). MAIT cells correlated with severity both at the pre- and post-treatment timepoints, whereas CD4 T cm were more predictive at the pretreatment stage and CD4 Tnaive correlated with post-treatment IRAE severity.

## Discussion

In this pilot study of the combination of sorafenib plus nivolumab in patients with advanced HCC, we aimed to determine the safety and MTD of this combination in patients with advanced HCC and varying degrees of liver dysfunction. The MTD was sorafenib 400 mg orally once daily in combination with nivolumab 240 mg intravenously every 2 weeks because of DLTs experienced at higher doses of sorafenib. TRAEs and IRAEs occurred at a similar frequency with combined sorafenib and nivolumab as historical rates for each drug as monotherapy.^33^^,^^36^ The rates of grade 3–4 TRAE of 70% in the Child-Pugh A cohort and 66.7% in the Child-Pugh B cohort were similar to the rates of grade 3 or higher TRAE in other studies of multikinase inhibitors combined with ICI in patients with Child-Pugh A disease, notably 81% in CARES-310, 55% in COSMIC-312, and 63% in LEAP-002.^21^^,^^22^^,^^51^ Likewise, the rate of TRAE in the Child-Pugh B subgroup was similar to that reported in other Child-Pugh B prospective studies. The rate of systemic steroid requirement for IRAEs was higher than expected for nivolumab as monotherapy in HCC but confounded by small sample size and overlapping attributions. Unfortunately, the ORR of 6% in this study did not meet the prespecified threshold for efficacy of 15% to warrant further investigation of the combination in patients with advanced HCC, regardless of liver function. Although the sample size was inadequately powered for efficacy assessment, the observed efficacy was insufficient to proceed with further study.

The expanding landscape of ICI-based combination therapies for advanced stages of HCC now includes the combination of atezolizumab plus bevacizumab, durvalumab plus a single dose of tremelimumab, and nivolumab plus ipilimumab, all of which prolong OS and achieve higher rates of objective response compared with sorafenib or lenvatinib as monotherapy.^7^^,^^20^^,^^23^^,^^52^ All of these regimens have been studied in patients with preserved Child-Pugh A liver function, whereas the optimal treatment regimen for patients with greater degrees of liver dysfunction, including the large population of patients with Child-Pugh B cirrhosis, has not been well established. An important aspect of our study was the inclusion of both patients with Child-Pugh A disease and those with Child-Pugh B disease. In this small cohort, the DCR, PFS, and OS were similar between Child-Pugh A and Child-Pugh B, although the small sample size limited comparisons. Historical data for sorafenib and other multikinase inhibitors or VEGF-directed therapies have demonstrated worse outcomes for patients with Child-Pugh B liver function.^35^^,^^36^^,^^53^^,^^54^ There are few prospective studies of ICI-based therapies in patients with Child-Pugh B HCC. In Cohort 5 of the Phase I/II CheckMate 040 study, nivolumab monotherapy achieved objective responses in 12% of patients, lower than the ORR of 20% in the Child-Pugh A cohort.^5^^,^^33^ A retrospective study of patients with Child-Pugh A and B liver function treated with atezolizumab and bevacizumab demonstrated similar response rates and TRAEs.^34^ ICI combination or monotherapy conferred a benefit in OS compared with best supportive care in patients with Child-Pugh B liver function (7.50 *vs.* 4.04 months).^55^ After completion of our study, a pooled analysis of 10 studies showed a higher ORR for ICI-based combination regimens than for ICI as monotherapy (19% *vs.* 12%) in patients with HCC with Child-Pugh B liver function,^28^ supporting the hypothesis that patients with Child-Pugh B liver function could also benefit from the combination approaches, although the absolute rates of objective response were lower than those observed in Child-Pugh A populations. Notably, the meta-analysis did not identify significant differences in TRAE rates, PFS, or OS according to Child-Pugh B subscore of 7 *vs.* 8/9, although sample sizes were limited.^28^ Others have found the Child-Pugh score insufficient to convey the severity of liver dysfunction and advocated for the incorporation of individual factors related to liver decompensation (*i.e.* ascites, hepatic encephalopathy).^56^ Given our small cohort, we were limited in our ability to ascertain the association of individual factors with outcomes and correlative data. In the context of these limited existing data, our trial of sorafenib combined with nivolumab supports that ICI combinations are feasible in carefully selected patients with Child-Pugh B HCC without excessive toxicity beyond that observed in Child-Pugh A populations.

Intriguingly, high-resolution scRNAseq of baseline peripheral blood samples obtained from patients enrolled on this trial showed a higher proportion of CD14_CTX_ in patients with HCC and worse liver function, suggesting a mechanism for the lower ORRs observed for combination ICI therapies in HCC with greater degrees of hepatic dysfunction. In BTC, CD14_CTX_ increased in frequency in the circulation of patients whose tumors were resistant to ICI and induced ‘immune-paralyzed’ CD4^+^ T cells characterized by *SOCS3* expression and low cytokine production and proliferative capacity.^46^ Although we observed the presence of these SOCS3^+^ CD4^+^ T cells and that they were negatively correlated with PFS, there was not an increased frequency of this specific CD4^+^ T cell subpopulation associated with Child-Pugh score in this HCC cohort; thus, there might be other mechanisms of suppression induced by these myeloid cells. Following treatment, we also observed a decrease in CD14_CTX_ in the Child-Pugh B subgroup, suggesting that there is a partial reversal of myeloid-mediated suppression following treatment with combined ICI/sorafenib, compatible with other reports of sorafenib-induced myeloid cell differentiation,^57^ and that perhaps other targeted therapies could improve on this reduction in suppressive monocytes.

In this small cohort, we did not observe meaningful differences in the baseline immune profile according to etiology of liver disease (viral *vs.* nonviral liver disease), and there were too few clinical responses to stratify by etiology. Although a meta-analysis suggested prolonged survival in virally associated compared with non-viral HCC,^58^ other studies of contemporary ICI-based combinations have shown no appreciable differences in outcome according to liver disease etiology in HCC.^7^^,^^52^^,^^59^^,^^60^ Given that metabolic dysfunction-associated steatotic liver disease is poised to become the most common cause of liver disease and HCC in the USA,^61^ further studies are needed to characterize both the peripheral blood and tumor immune microenvironments according to etiology of liver disease and identify candidate biomarkers as well as therapeutic targets that might be unique to a specific etiology.

Using serial samples collected in this study, we also investigated how combined sorafenib and nivolumab affected the circulating immune response with multi-omic scRNAseq as a paradigm for elucidating mechanisms of response or resistance to other immunotherapy combination regimens in HCC. Although this was an exploratory analysis because of the limited number of patients with clinical benefit, we found that patients who had disease control had increased CD8^+^ T cells following treatment; when we further evaluated T cell subsets, we found that the T cells most correlated with increased PFS were effector memory CD8^+^ T cells expressing Granzyme K and MAIT cells, two T cell subsets that might contribute to both antitumor responses and the response to ICI.^62^^,^^63^ Although the total frequency of circulating monocytes was not altered across the cohort, there were alterations in the frequency of monocyte subsets within the DC group, including a population previously associated with ICI response (CD14_APC_) and a concomitant decrease in suppressive CD14_CTX_ monocytes.^46^ This aligned with enrichment of immunostimulatory pathways within monocytes in the DC group (*i.e.* IFN signaling) and immunosuppressive pathways in monocytes in the NR group (*i.e.* IL-6/JAK/STAT3 signaling). Furthermore, the CD14^+^ monocytes in the DC group had upregulation of glycolysis (*vs.* oxidative phosphorylation in the NR group) with treatment, which has been demonstrated to be induced in the setting of productive immune responses.^64^^,^^65^ Lastly, the correlated frequencies of immunostimulatory monocytes and effector T cells, and the upregulated antitumor pathways in both cell types suggest that there is a parallel shift in T cells and monocytes to a favorable immune program following combination immunotherapy. However, given the limited benefit of this regimen clinically, these immune alterations might only be induced in a minority of patients. Therefore, our findings point to mechanisms of immunotherapy response and resistance that must be more fully addressed by HCC regimens to improve clinical efficacy.

We also performed an exploratory analysis of immune signatures associated with the severity of IRAEs with intriguing findings. First, a myeloid population negatively associated with ICI response in this cohort and in BTC, CD14_CTX_, was associated with worsening grade IRAEs. Given previous findings of IRAE association with response to ICI, this was surprising, although, in our cohort, we did not have a sufficient number to identify whether IRAEs correlated with response. Second, although antigen-presenting cells could initiate IRAEs by presenting host antigens, we found that myeloid populations with gene signatures related to antigen presentation (DC-like monocytes, cDCs, and CD14_APC_) were negatively correlated with IRAE severity. Finally, we found that MAIT cells were associated with worsening IRAEs. Given that MAIT cells have been previously identified in both ulcerative and ICI-related colitis and have a role in regulating mucosal immunity, this could explain both their predictive and mechanistic role in IRAEs.^66^^,^^67^

Our study has several limitations, including small sample size and the resulting exploratory nature of the single cell analysis. The small sample size and slower-than-expected enrollment resulted, in part, from the changing treatment landscape for HCC with publication of the IMbrave150 trial soon after inception of our study, as well as the COVID-19 pandemic. In addition, we were only able to study the circulating immune responses because biopsy samples were beyond the scope of this investigator-initiated clinical trial. Thus, we correlated our PBMC results with tumor resection specimens from a separate cohort of patients with single cell methods and identified highly correlated monocyte/macrophage populations, including an SPP1^+^ macrophage correlated with CD14_CTX._ In HCC, SPP1^+^ macrophages inhabit ‘tumor immune barriers’ that prohibit immune cell infiltration into tumors, decreasing the efficacy of immunotherapy.^68^ SPP1^+^ macrophages have been shown to be suppressive of immunotherapy responses and associated with worse prognosis in other tumor types, demonstrating the clinical relevance of this population.^44^^,^^69^^,^^70^

Regardless of the limitations, the insights here are hypothesis generating for future studies of other treatment regimens now being used in the first-line setting in HCC, for which immune-based mechanisms of response and primary resistance are poorly understood, both in the overall population and specific to greater degrees of hepatic dysfunction. The safety and efficacy of ICI combination therapies in the setting of greater degrees of hepatic dysfunction remains an active area of ongoing clinical research, with multiple trials exploring safety and efficacy outcomes in patients with HCC and more advanced liver dysfunction (NCT05883644 and NCT06096779). Patients with worse immune dysfunction caused by liver disease could stand to benefit more from combination regimens that can target multiple mechanisms of immunotherapy resistance, including myeloid cells. Thus, the intersection of specific treatment regimens, safety in the context of liver dysfunction, and immunotherapy response is an important area for future studies to optimize and personalize therapy in HCC.

## Conclusions

Although the combination of sorafenib and nivolumab demonstrated acceptable safety in both Child-Pugh A and B subgroups in this study, the limited efficacy in this small cohort does not warrant further investigation. However, a distinct population of immunosuppressive myeloid cells was identified at baseline in the circulation in patients with Child-Pugh B hepatic dysfunction and warrants further study as a potential contributor to therapeutic resistance. Systemic therapy options for patients with greater degrees of liver dysfunction remain a significant unmet need, requiring further understanding of the distinct mechanisms of immune response and resistance in this population.

## Abbreviations

ALBI, albumin–bilirubin; BCLC, Barcelona Clinic Liver Cancer; BTC, biliary tract cancer; cDC, conventional dendritic cells; CITEseq, cellular indexing of transcriptomes and epitopes by sequencing; cMono, CD14^+^ classical monocytes; CT, computed tomography; DC, disease control; DL, dose level; DLT, dose-limiting toxicity; DOR, duration of response; HCC, hepatocellular carcinoma; HR, hazard ration; ICI, Immune checkpoint inhibition; IRAE, immune-related adverse event; MAIT, mucosal-associated invariant T cells; MRI, magnetic resonance imaging; MTD, maximum-tolerated dose; ncMono, CD16^+^ non-classical monocytes; NE, not evaluable; NES, normalized enrichment score; NK, natural killer cells; NR, non-response; ORR, objective response rate; OS, overall survival; PBMC, peripheral blood mononuclear cells; PD-1, Programmed cell death protein 1; PD-L1, Programmed cell death ligand 1; PD, progressive disease; pDC = plasmacytoid dendritic cells; pDC, plasmacytoid dendritic cells; PFS, progression-free survival; PFS6, PFS of 6 months or longer; Plasma, plasma cells; PR, partial response; scRNAseq, single cell RNA sequencing; Tcm, central memory; Tcyto, cytotoxic T cells; Tem, effector memory; Temra, terminally differentiated re-expressing CD45RA effector memory; Tprolif, proliferating T cells; Treg, regulatory T cell; UMAP, Uniform Manifold Approximation and Projection; VEGF, vascular endothelial growth factor; VEGFR, vascular endothelial growth factor receptor.

## Authors’ contributions

Conceptualization, funding acquisition, writing – original draft: BPK, RKK. Data curation: BPK, ZF, BKL, QH, JC, MC, AL, AC, FL, EJK, RKK. Formal analysis: BPK, ZF, LZ, PB, SCB, RKK. Investigation: BPK, ZF, BKL, QH, JC, MC, AL, AC, FL, JDG, APV, EJK, RKK. Methodology: BPK, ZF, MC, AL, RKK. Project administration: BPK, AC, RKK. Resources: BPK, ZF, JDG, LF, APV, EJK, RKK. Software: BPK, ZF, LZ. Supervision: BPK, LF, RKK. Visualization: BPK, ZF, LZ, RKK. Writing – review & editing: BPK, ZF, JDG, LF, RKK.

## Data availability

scRNAseq data are available on NCBI GEO (GSE318420, Supplemental CTAT Table). Additional clinical trial data will be made available upon request.

## Financial support

We acknowledge financial support from 10.13039/100004326Bayer (to institution), 10.13039/100002491Bristol Myers Squibb (nivolumab drug supply), Bili Project Foundation, Inc., and the imCORE 10.13039/100022394Network through 10.13039/100004328Genentech Inc (correlative studies). This work was also funded, in part, by research grants 10.13039/100000002NIH K08CA290152, 10.13039/100000002NIH 1K12CA260225-01, 10.13039/100001021Damon Runyon Clinical Investigator Award, and UCSF Liver Center Pilot Award to BPK, and funding from the Shorenstein Family to BPK and APV.

## Conflicts of interests

BPK has received research funding (to university) from Apexigen/Pyxis Oncology, Affini-T Therapeutics, Antengene, Innovative Cellular Therapeutics, Wugen, Astra Zeneca, Takeda, Roche/Genentech, Regeneron, and Boehringer Ingelheim; consulting or advisory fees (to self) from Regeneron, Agenus Inc., Cartography, and Arcellx; and travel expenses from Roche/Genentech. LZ has received consulting or advisory fees (to self) from Motive Medical Intelligence, Smith-Kettlewell Eye Research Institute, and Serna Bio. SCB has received consulting or advisory fees (to self) from Novartis and RiboScience. LF has received research funding (to university) from Roche/Genentech, Abbvie, Bavarian Nordic, Bristol Myers Squibb, Dendreon, Janssen, Merck, and Partner Therapeutics; and consulting or advisory fees (to self) from Actym, Astra Zeneca, Bioatla, Bristol Myers Squibb, Daiichi Sankyo, Immunogenesis, Innovent, Merck, Sutro, and Roche/Genentech. APV has received consulting or advisory fees (to self) from Agenus Bio. EJK has received research funding (to institution) from Bayer, Merck, EpicentRx, Astellas, Erytech, Fibrogen, NGM Biopharmaceuticals, Eureka, Genentech, GSK, VCN Biosciences, Incyte, Medimmune, Tizona, Celgene, Exelixis, Inc., Bristol Myers Squibb, Astra Zeneca, and Beigene; and consulting or advisory fees (to self): Eisai, Astra Zeneca, Seagen, Pfizer, and Relay Therapeutics. RKK has received research funding (to institution) from Agios, Astra Zeneca, Bayer, Bristol Myers Squibb, Compass Therapeutics, Eli Lilly, EMD Serono, Exelixis, Genentech/Roche, Merck, Partner Therapeutics, QED, Relay Therapeutics, Servier, Surface Oncology, Taiho, and Tyra Biosciences; consulting or advisory fees (to institution) from Astra Zeneca and Merck; and consulting or advisory fees (to self) from Compass Therapeutics, CVS Caremark, Elevar, GenMab, GSK, Jazz, J Pharma, Moderna, Regeneron, and Tyra Biosciences. The remaining authors declare no conflicts of interests that pertain to this work.

Please refer to the accompanying ICMJE disclosure forms for further details.
