# Supplementary material for: Phase II trial with nivolumab and sorafenib in HCC identified enrichment of immunosuppressive monocytes in patients with Child-Pugh B liver dysfunction
Source: JHEP Rep. 2026 Mar 12;8(5):101817. doi: 10.1016/j.jhepr.2026.101817 (PMC13101793; doi:10.1016/j.jhepr.2026.101817)
Supplement: Multimedia component 1 [file mmc1.pdf]

# Phase II trial with nivolumab and sorafenib in HCC identified enrichment of immunosuppressive monocytes in patients with Child-Pugh B liver dysfunction

Bridget P. Keenan, Zenghua Fan, Bryan Khuong Le, Quincy Harris, Jocelin Chen, Matthew Clark, Averey Lea, Li Zhang, Alexander Cheung, Frances Lara, John D. Gordan, Paige Bracci, Spencer C. Behr, Lawrence Fong, Alan P. Venook, Edward J. Kim[5], Robin K. Kelley

Table of contents

Fig. S1……2

Fig. S2……3

Fig. S3……5

Fig. S4……6

Fig. S5……8

Table S1……9

Table S2……10

Table S3……11

Table S4……13

Table S5……15

Table S6……16

Table S7……17

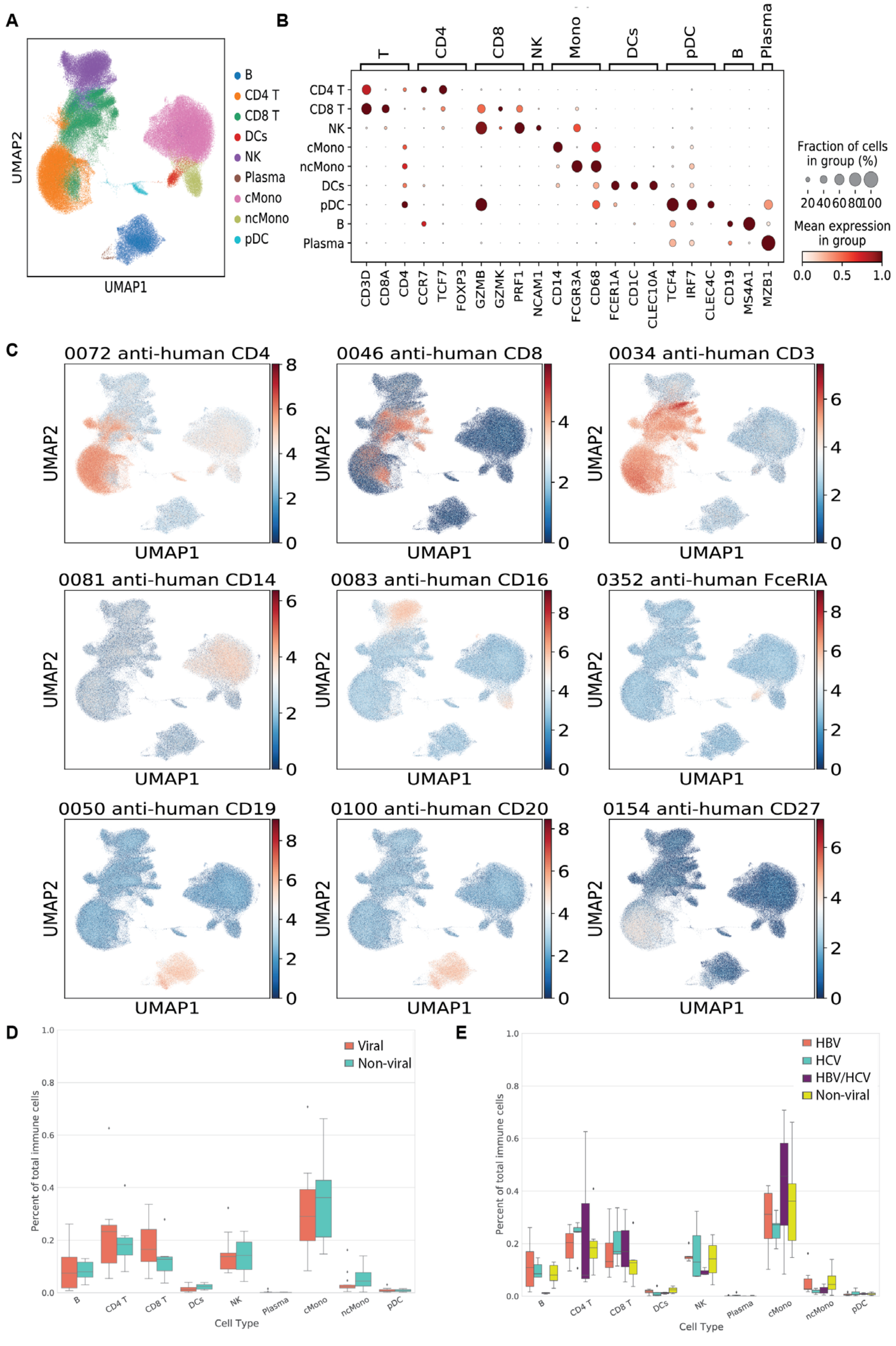


**Fig. S1. Characterization of circulating immune cells from patients with advanced HCC with CITEseq. A.** Uniform Manifold Approximation and Projection (UMAP) plot of single cell RNA sequencing results from PBMCs colored by immune cell type. **B.** Dotplot demonstrating expression level and the fraction of cells within each immune cell population (y-axis) expressing the RNA molecules indicated. The groupings on the x-axis correspond to the category of markers used to define populations. **C.** UMAP colored by protein expression as indicated by the CITEseq antibody at the top of each plot. **D-E.** Frequency of immune cell type at baseline (pre) for all patients (n=20) by viral status (viral (n=14) or non-viral (n=6) (**D**) and specific viral associations (HBV: n=6, dual HBV/HCV: n=3, HCV: n=5) (**E**)). Boxes denote inter-quartile range (IQR) while bars denote 25% - 1.5xIQR and 75% + 1.5xIQR.

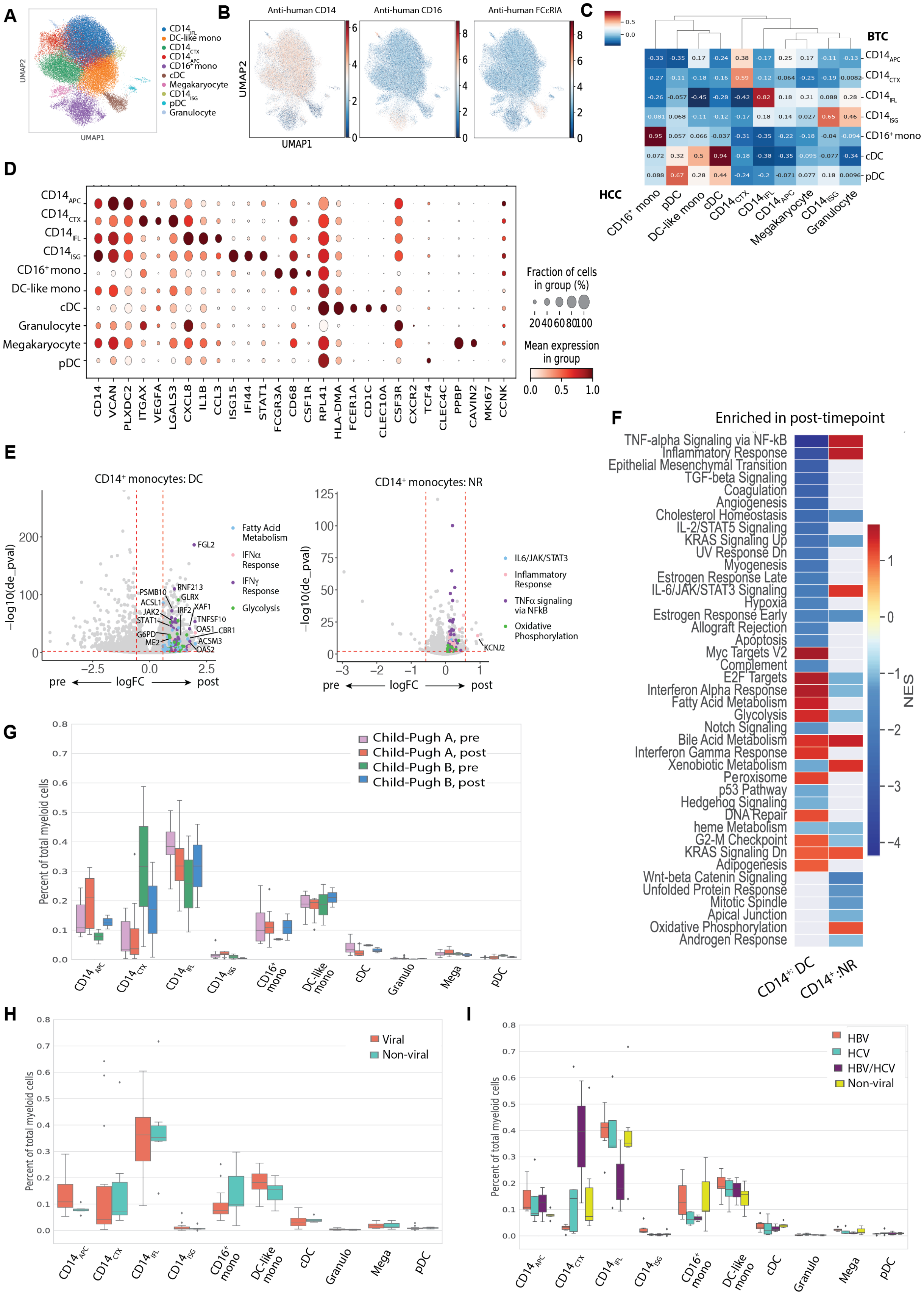
A
CD14 IFL
DC-like mono
CD14 CTX
CD14 APC
CD16+ mono
cDC
Megakaryocyte
CD14 ISG
pDC
Granulocyte
UMAP2
UMAP1
B
Anti-human CD14
Anti-human CD16
Anti-human FCεRIA
C
BTC
HCC
CD16+ mono
pDC
DC-like mono
cDC
CD14 CTX
CD14 IFL
CD14 APC
Megakaryocyte
CD14 ISG
Granulocyte
D
Fraction of cells in group (%)
Mean expression in group
CD14
VCAN
PLXDC2
ITGAX
VEGFA
LGALS3
CXCL8
IL1B
CCL3
ISG15
IFI44
STAT1
FCGR3A
CD68
CSF1R
RPL41
HLA-DMA
FCER1A
CD1C
CLEC10A
CSF3R
CXCR2
TCF4
CLEC4C
PPBP
CAVIN2
MKI67
CCNK
E
CD14+ monocytes: DC
CD14+ monocytes: NR
-log10(de_pval)
pre
logFC
post
Fatty Acid Metabolism
IFNα Response
IFNγ Response
Glycolysis
IL6/JAK/STAT3
Inflammatory Response
TNFα signaling via NFkB
Oxidative Phosphorylation
F
Enriched in post-timepoint
TNF-alpha Signaling via NF-kB
Inflammatory Response
Epithelial Mesenchymal Transition
TGF-beta Signaling
Coagulation
Angiogenesis
Cholesterol Homeostasis
IL-2/STAT5 Signaling
KRAS Signaling Up
UV Response Dn
Myogenesis
Estrogen Response Late
IL-6/JAK/STAT3 Signaling
Hypoxia
Estrogen Response Early
Allograft Rejection
Apoptosis
Myc Targets V2
Complement
E2F Targets
Interferon Alpha Response
Fatty Acid Metabolism
Glycolysis
Notch Signaling
Bile Acid Metabolism
Interferon Gamma Response
Xenobiotic Metabolism
Peroxisome
p53 Pathway
Hedgehog Signaling
DNA Repair
heme Metabolism
G2-M Checkpoint
KRAS Signaling Dn
Adipogenesis
Wnt-beta Catenin Signaling
Unfolded Protein Response
Mitotic Spindle
Apical Junction
Oxidative Phosphorylation
Androgen Response
NES
CD14+: DC
CD14+ :NR
G
Child-Pugh A, pre
Child-Pugh A, post
Child-Pugh B, pre
Child-Pugh B, post
Percent of total myeloid cells
H
Viral
Non-viral
I
HBV
HCV
HBV/HCV
Non-viral

**Fig. S2. Characterization of circulating myeloid cells from patients with advanced HCC and association with clinical response and liver disease characteristics. A.** Uniform Manifold Approximation and Projection (UMAP) plot of single cell RNA sequencing results from PBMCs colored by myeloid cell sub-type. **B.** UMAP colored by protein expression as indicated by the CITEseq antibody at the top of each plot. **C.** Comparison of gene signatures for each myeloid cell type in the HCC dataset (x-axis) with myeloid cells from the BTC dataset (y-axis) using pseudobulk gene expression. Legend shows correlation values demonstrated in heatmap format. **D.** Dotplot demonstrating expression level and the fraction of cells within each myeloid cell population (y-axis) expressing the RNA molecules indicated. **E.** Differentially expressed genes in post-treatment vs pre-treatment timepoints for all $CD14^{+}$ monocytes in the DC and NR groups are plotted, with genes of interest in the indicated pathways highlighted with the corresponding color dot. **F.** Pathways enriched within all $CD14^{+}$ monocytes ($CD14_{ISG}$, $CD14_{CTX}$, $CD14_{APC}$, $CD14_{IFL}$, and DC-like monocytes) (x-axis) in the post-treatment compared to pre-treatment timepoint are shown on the y-axis; heatmap corresponds to normalized enrichment score (NES). Columns correspond to the disease control (DC) or non-responder (NR) groups. **G.** Percent of each cell type out of total myeloid cells in circulation for patients with Child-Pugh A (n=10) or Child-Pugh B (n=4) prior to treatment (pre) and following one cycle of combination treatment (post). **H-I.** Frequency of myeloid cell type out of total circulating myeloid cells at baseline (pre) for all patients (n=20) by viral status (viral (n=14) or non-viral (n=6)) (**B**) and specific viral associations (HBV: n=6, dual HBV/HCV: n=3, HCV: n=5) (**C**). Boxes denote inter-quartile range (IQR) while bars denote 25% - 1.5xIQR and 75% + 1.5xIQR.

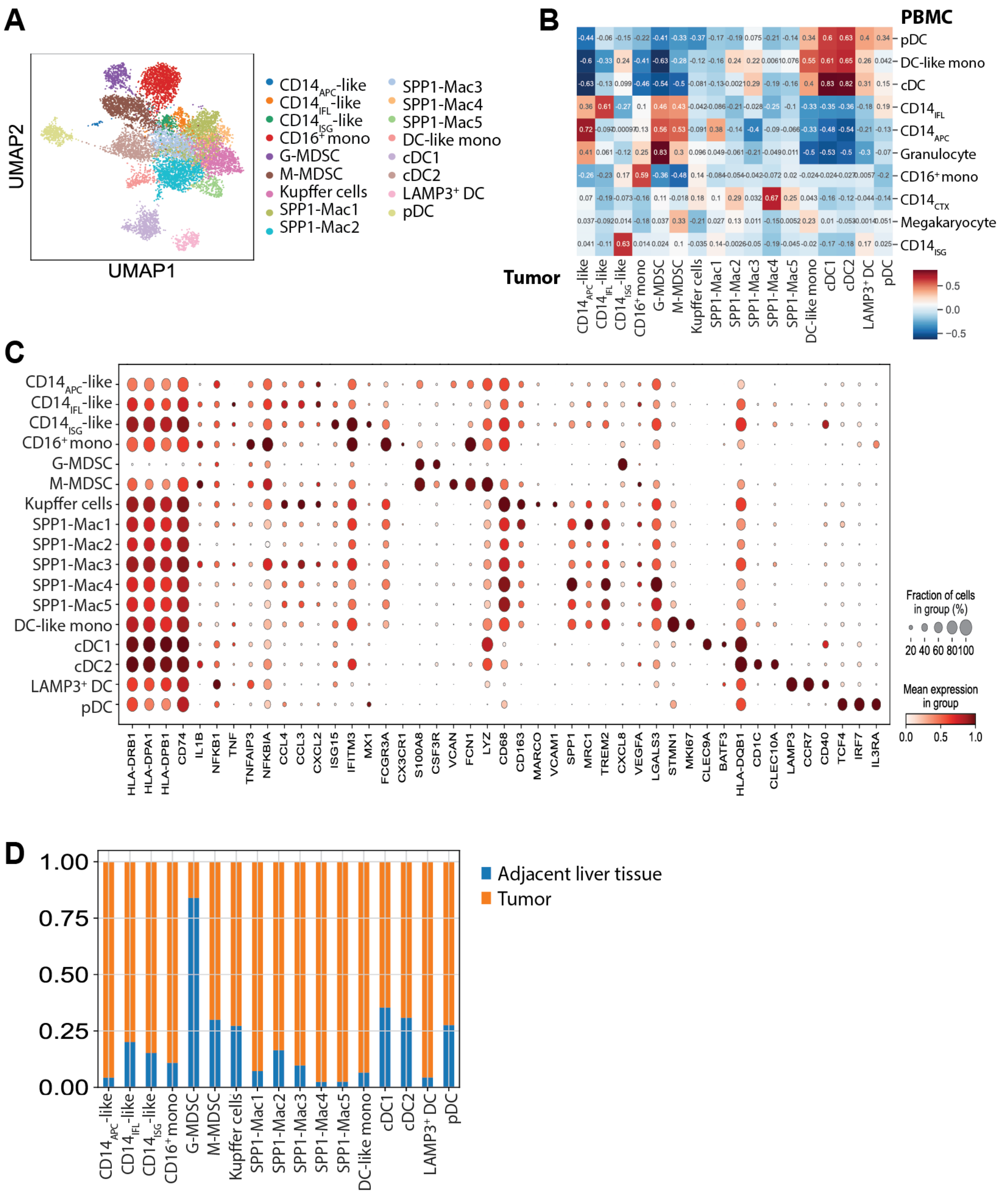


**Fig. S3. Intra-tumoral myeloid cells from HCC tumor tissues and comparison to circulating myeloid cell populations. A.** UMAP plot of single cell RNA sequencing results from tumor and adjacent liver tissue colored by myeloid cell sub-type. (MDSC = myeloid-derived suppressor cell, Mac = macrophage). **B.** Comparison of gene signatures for each myeloid cell type in the HCC tumor dataset (x-axis) with myeloid cells from the HCC PBMC dataset (y-axis) using pseudobulk gene expression. Legend shows correlation values demonstrated in heatmap format. **C.** Dotplot demonstrating expression level and the fraction of cells within each myeloid cell population (y-axis) expressing the RNA molecules indicated. **D.** Barplot showing the fraction of each population made up by the cell type from either the tumor specimens or adjacent liver tissue.

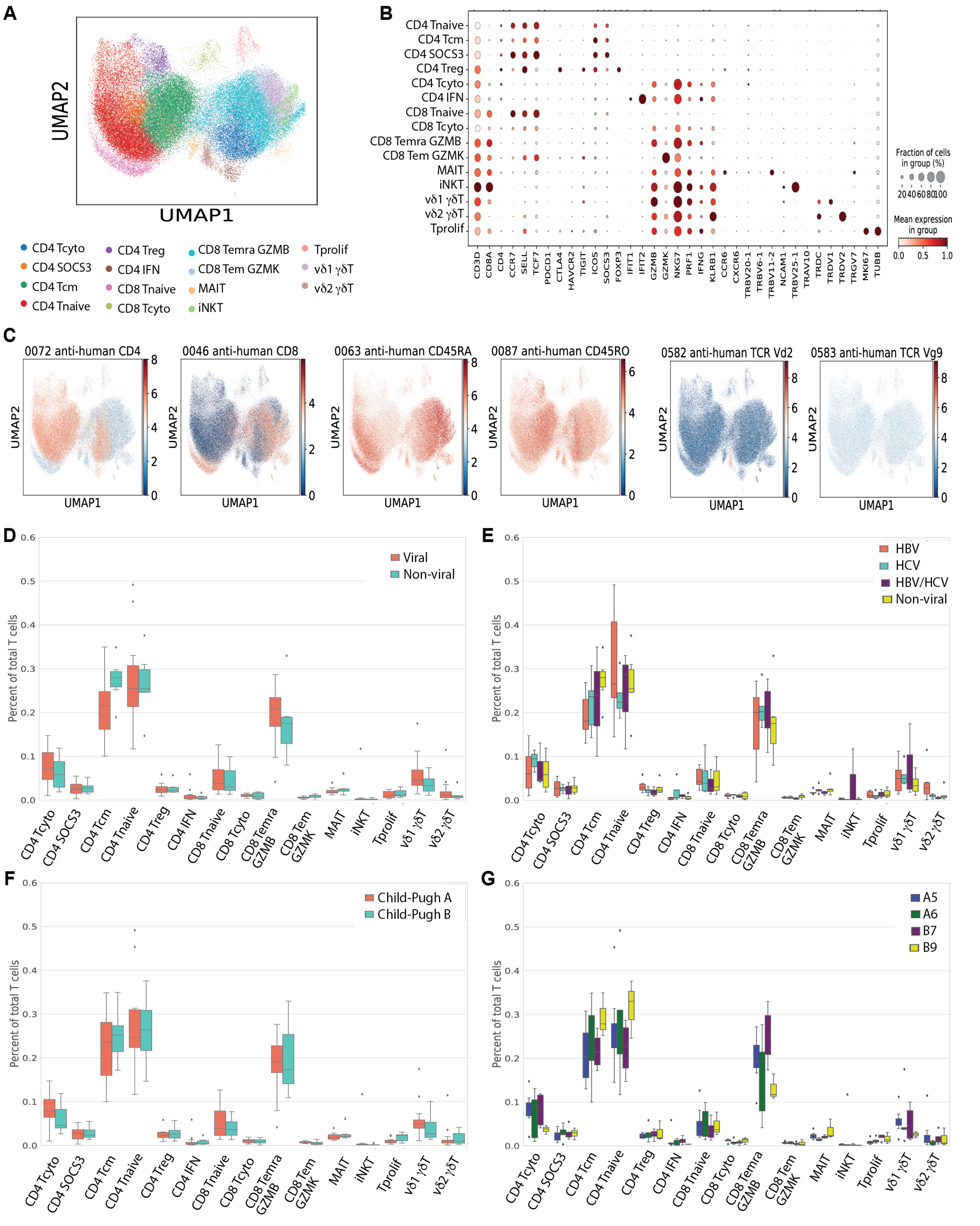


**Fig. S4. Characterization of circulating T cells from patients with advanced HCC with CITEseq. A.** Uniform Manifold Approximation and Projection (UMAP) plot of single cell RNA sequencing results from PBMCs colored by T cell sub-type. **B.** Dotplot demonstrating expression level and the fraction of cells within each T cell population (y-axis) expressing the RNA molecules indicated. The groupings on the x-axis correspond to the category of markers

used to define populations. **C.** UMAP colored by protein expression as indicated by the CITEseq antibody at the top of each plot. **D-G.** Frequency of T cell type out of total circulating T cells at baseline (pre) for all patients (n=20) by viral status (viral (n=14) or non-viral (n=6)) (**D**), specific viral associations (HBV: n=6, dual HBV/HCV: n=3, HCV: n=5) (**E**)) and by Child-Pugh status (A (n=13) vs B (n=7) (**F**), Child-Pugh sub-score (A5: n=8, A6: n= 5, B7: n=4, B9: n=3) (**G**)). Boxes denote inter-quartile range (IQR) while bars denote 25% - 1.5xIQR and 75% + 1.5xIQR.

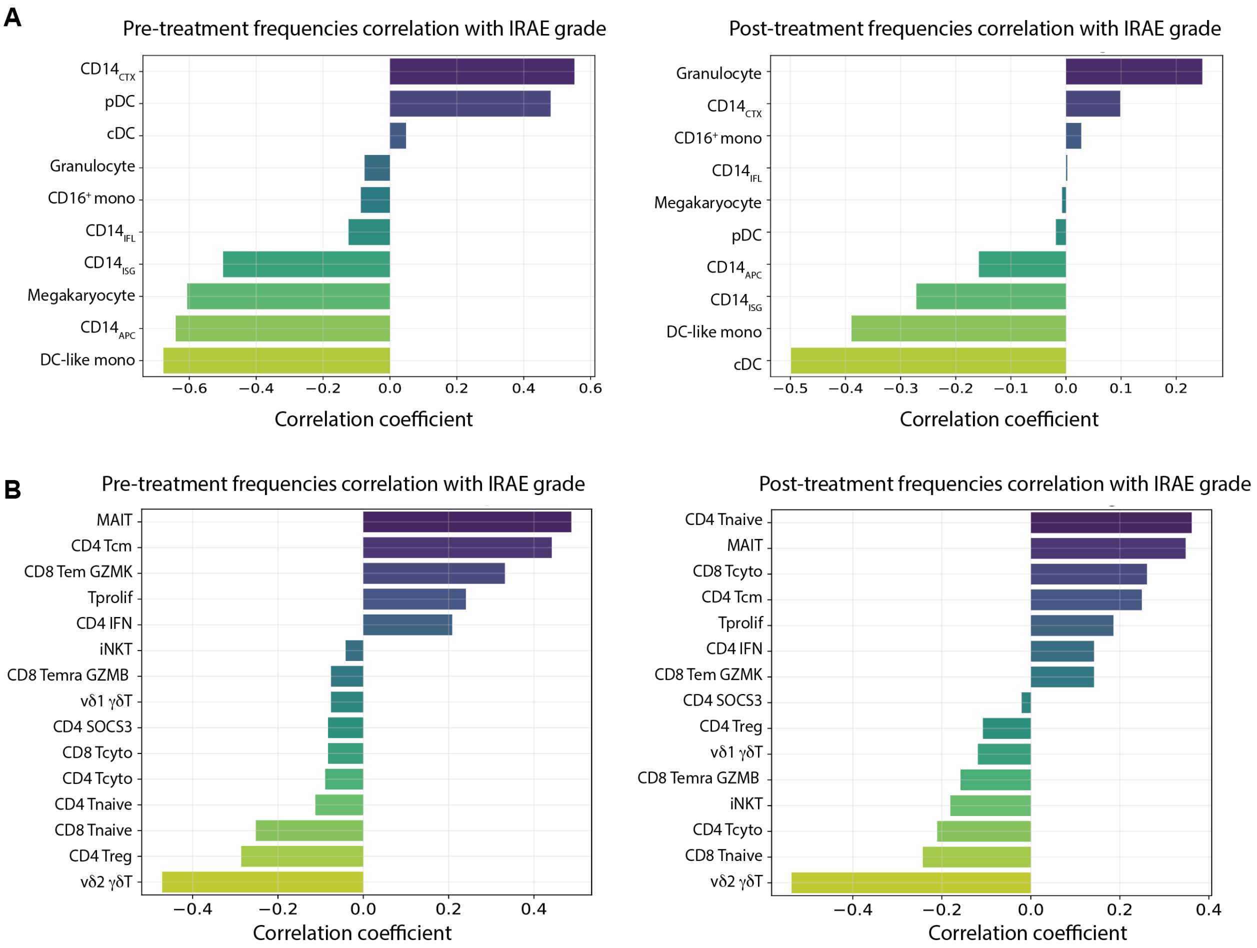


**Fig. S5. Association of circulating myeloid and T cells with immune-related adverse events (IRAEs). A.** Correlation coefficients for the frequency of each myeloid cell sub-type at the pre- and post-treatment timepoints and grade of IRAE in patients that experienced IRAEs. **B.** Correlation coefficients for the frequency of each T cell sub-type at the pre- and post-treatment timepoints and grade of IRAE in patients that experienced IRAEs.

**Table S1. Demographic characteristics of patients with collected PBMCs for analysis.**

| **Demographic** | | |
|---|---|---|
| | | **Screening only cohort** |
| | | **n=5** |
| Median age, years (SD) | | 65 (5.3) |
| Male, n (%) | | 4 (80) |
| Race, n (%) | White | 4 (80) |
| | Black or African American | 0 |
| | Asian | 1 (20) |
| | American Indian or Alaska Native | 0 |
| Ethnicity | Non-Hispanic/Latinx | 4 (80) |
| | Hispanic/Latinx | 1 (20) |
| Viral status | HBV+* | 1 (20) |
| | HCV+* | 2 (40) |
| | Non-viral | 2 (40) |
| Child-Pugh score at | A5 | 2 (40) |
| screening | A6 | 1 (20) |
| | B7 | 1 (20) |
| | B9 | 1 (20) |
| ALBI grade at enrollment | 1 | 0 |
| | 2 | 2 (40) |
| | 3 | 3 (60) |

Patients included in the table were screened and not enrolled in the Phase II clinical trial of combination sorafenib and nivolumab.
*Some patients had both HBVcAb+ and HCV antibody positive, but were HBsAg negative

**Table S2. Clinical characteristics of separate HCC tumor resection cohort.**

| Patient ID | Viral Status | Age at collection | Race | Ethnicity | TNM staging | BCLC stage | Child-Pugh stage |
|---|---|---|---|---|---|---|---|
| A-12 | non-viral | 64 | White | Non-Hispanic/Latinx | IA | 0 | A |
| A-18 | HBV+HCV+ | 58 | Asian | Non-Hispanic/Latinx | IB | A | A |
| A-19 | HBV+ | 66 | Asian | Non-Hispanic/Latinx | II | A | A |
| A-20 | HBV+ | 58 | Asian | Non-Hispanic/Latinx | IA | 0 | A |
| A-23 | non-viral | 81 | White | Non-Hispanic/Latinx | IIIA | A | A |
| A-24 | HBV+ | 64 | Asian | Non-Hispanic/Latinx | II | A | A |
| A-30 | non-viral | 67 | White | Non-Hispanic/Latinx | IIIA | A | A |

**Table S3. Treatment-related adverse event (TRAE) occurring in more than one patient by grade and dose level.**

| **Dose Level -1 (n=11)** | | | |
|---|---|---|---|
| **Common TRAEs (n>1 in total cohort)** | | | |
| **AE** | **Grade 1/2** | **Grade 3/4** | **Any grade** |
| Rash | 7 (63.6) | 1 (9.1) | 8 (72.7) |
| Hypertension | 5 (45.5) | 1 (9.1) | 6 (54.5) |
| Increased aspartate aminotransferase | 2 (18.2) | 3 (27.3) | 5 (45.5) |
| Diarrhea | 4 (36.4) | 0 | 4 (36.4) |
| Decreased appetite/anorexia | 3 (27.3) | 0 | 3 (27.3) |
| Emesis | 3 (27.3) | 0 | 3 (27.3) |
| Hoarseness | 3 (27.3) | 0 | 3 (27.3) |
| Increased alanine aminotransferase | 3 (27.3) | 0 | 3 (27.3) |
| Myalgia | 3 (27.3) | 0 | 3 (27.3) |
| Mucositis | 2 (18.2) | 1 (9.1) | 3 (27.3) |
| Nausea | 3 (27.3) | 0 | 3 (27.3) |
| Palmar-plantar erythrodysesthesia syndrome | 2 (18.2) | 1 (9.1) | 3 (27.3) |
| Pruritus | 3 (27.3) | 0 | 3 (27.3) |
| Dry skin | 2 (18.2) | 0 | 2 (18.2) |
| Fatigue | 2 (18.2) | 0 | 2 (18.2) |
| Weight loss | 2 (18.2) | 0 | 2 (18.2) |
| Constipation | 1 (9.1) | 0 | 1 (9.1) |
| Headache | 1 (9.1) | 0 | 1 (9.1) |
| Hyperbilirubinemia | 0 | 1 (9.1) | 1 (9.1) |
| Hypoalbuminemia | 1 (9.1) | 0 | 1 (9.1) |
| Dry eye | 0 | 0 | 0 |
| Dyspepsia | 0 | 0 | 0 |
| Hyponatremia | 0 | 0 | 0 |

| **Dose Level 1 (n=5)** | | | |
|---|---|---|---|
| **Common TRAEs (n>1 in total cohort)** | | | |
| **AE** | **Grade 1/2** | **Grade 3/4** | **Any grade** |
| Rash | 3 (60) | 0 | 3 (60) |
| Constipation | 2 (40) | 0 | 2 (40) |
| Diarrhea | 2 (40) | 0 | 2 (40) |
| Dry eye | 2 (40) | 0 | 2 (40) |
| Dry skin | 2 (40) | 0 | 2 (40) |

| | | | |
|---|---|---|---|
| Dyspepsia | 2 (40) | 0 | 2 (40) |
| Hyperbilirubinemia | 0 | 2 (40) | 2 (40) |
| Hyponatremia | 1 (20) | 1 (20) | 2 (40) |
| Increased alanine aminotransferase | 2 (40) | 0 | 2 (40) |
| Increased aspartate aminotransferase | 2 (40) | 0 | 2 (40) |
| Myalgia | 2 (40) | 0 | 2 (40) |
| Palmar-plantar erythrodysesthesia syndrome | 0 | 2 (40) | 2 (40) |
| Headache | 1 (20) | 0 | 1 (20%) |
| Hoarseness | 1 (20) | 0 | 1 (20%) |
| Hypoalbuminemia | 1 (20) | 0 | 1 (20%) |
| Fatigue | 0 | 1 (20%) | 1 (20%) |
| Mucositis | 1 (20%) | 0 | 1 (20%) |
| Decreased appetite/anorexia | 0 | 0 | 0 |
| Emesis | 0 | 0 | 0 |
| Hypertension | 0 | 0 | 0 |
| Nausea | 0 | 0 | 0 |
| Pruritus | 0 | 0 | 0 |
| Weight loss | 0 | 0 | 0 |

**Table S4. Treatment-related adverse event (TRAE) occurring in more than one patient by grade and Child-Pugh status.**

| | | | |
|---|---|---|---|
| **Child Pugh A (n=10)** | | | |
| **Common TRAEs (n>1 in total cohort)** | | | |
| **AE** | **Grade 1/2** | **Grade 3/4** | **Any grade** |
| Rash | 7 (70) | 1 (10) | 8 (80) |
| Increased alanine aminotransferase | 4 (40) | 0 | 4 (40) |
| Increased aspartate aminotransferase | 4 (40) | 0 | 4 (40) |
| Palmar-plantar erythrodysesthesia syndrome | 1 (10) | 3 (30) | 4 (40) |
| Decreased appetite/anorexia | 3 (30) | 0 | 3 (30) |
| Diarrhea | 3 (30) | 0 | 3 (30) |
| Fatigue | 2 (20) | 1 (10) | 3 (30) |
| Hoarseness | 3 (30) | 0 | 3 (30) |
| Hypertension | 2 (20) | 1 (10) | 3 (30) |
| Mucositis | 3 (30) | 0 | 3 (30) |
| Myalgia | 3 (30) | 0 | 3 (30) |
| Constipation | 2 (20) | 0 | 2 (20) |
| Dry eye | 2 (20) | 0 | 2 (20) |
| Dry skin | 2 (20) | 0 | 2 (20) |
| Dyspepsia | 2 (20) | 0 | 2 (20) |
| Emesis | 2 (20) | 0 | 2 (20) |
| Headache | 2 (20) | 0 | 2 (20) |
| Hypoalbuminemia | 2 (20) | 0 | 2 (20) |
| Hyponatremia | 1 (10) | 1 (10) | 2 (20) |
| Nausea | 2 (20) | 0 | 2 (20) |
| Pruritus | 2 (20) | 0 | 2 (20) |
| Weight loss | 2 (20) | 0 | 2 (20) |
| Hyperbilirubinemia | 0 | 1 (10) | 1 (10) |
| **Other Gr 3/4 TRAEs (n=1 in total cohort)** | | | |
| Keratoacanthoma/florid lichenoid skin reaction | | 1 (10) | |
| **Individual patients with Gr3/4 TRAE** | | **7 (70)** | |
| | | | |
| **Child Pugh B (n=6)** | | | |
| **Common TRAEs (n>1 in total cohort)** | | | |
| **AE** | **Grade 1/2** | **Grade 3/4** | **Any grade** |
| Diarrhea | 3 (50) | 0 | 3 (50) |
| Hypertension | 3 (50) | 0 | 3 (50) |

| | | | |
|---|---|---|---|
| Increased aspartate aminotransferase | 0 | 3 (50) | 3 (50) |
| Rash | 3 (50) | 0 | 3 (50) |
| Dry skin | 2 (33.3) | 0 | 2 (33.3) |
| Hyperbilirubinemia | 0 | 2 (33.3) | 2 (33.3) |
| Myalgia | 2 (33.3) | 0 | 2 (33.3) |
| Constipation | 1 (16.7) | 0 | 1 (16.7) |
| Emesis | 1 (16.7) | 0 | 1 (16.7) |
| Hoarseness | 1 (16.7) | 0 | 1 (16.7) |
| Increased alanine aminotransferase | 1 (16.7) | 0 | 1 (16.7) |
| Mucositis | 0 | 1 (16.7) | 1 (16.7) |
| Nausea | 1 (16.7) | 0 | 1 (16.7) |
| Palmar-plantar erythrodysesthesia syndrome | 1 (16.7) | 0 | 1 (16.7) |
| Pruritus | 1 (16.7) | 0 | 1 (16.7) |
| Decreased appetite/anorexia | 0 | 0 | 0 |
| Dry eye | 0 | 0 | 0 |
| Dyspepsia | 0 | 0 | 0 |
| Headache | 0 | 0 | 0 |
| Hypoalbuminemia | 0 | 0 | 0 |
| Hyponatremia | 0 | 0 | 0 |
| Fatigue | 0 | 0 | 0 |
| Weight loss | 0 | 0 | 0 |
| **Other Gr 3/4 TRAEs (n=1 in total cohort)** | | | |
| Immune related hepatitis | | 1 (16.7) | |
| **Individual patients with Gr3/4 TRAE** | | **4 (66.7)** | |

**Table S5. Immune-related adverse event (IRAE) occurring in more than one patient by grade.**

| Immune-related adverse event occurring in n>1 patient, n (%) | Grade 1 or 2 | Grade 3 or 4 | Any grade |
|---|---|---|---|
| Rash | 9 (56.3) | 0 | 9 (56.3) |
| Increased aspartate aminotransferase | 3 (18.8) | 2 (12.5) | 5 (31.3) |
| Diarrhea | 3 (18.8) | 0 | 3 (18.8) |
| Fatigue | 2 (12.5) | 1 (6.25) | 3 (18.8) |
| Mucositis | 2 (12.5) | 1 (6.25) | 3 (18.8) |
| Myalgia | 3 (18.8) | 0 | 3 (18.8) |
| Constipation | 2 (12.5) | 0 | 2 (12.5) |
| Decreased appetite | 2 (12.5) | 0 | 2 (12.5) |
| Dry eye | 2 (12.5) | 0 | 2 (12.5) |
| Dry skin | 2 (12.5) | 0 | 2 (12.5) |
| Emesis | 2 (12.5) | 0 | 2 (12.5) |
| Hyperbilirubinemia | 1 (6.25) | 1 (6.25) | 2 (12.5) |
| Hyponatremia | 1 (6.25) | 1 (6.25) | 2 (12.5) |
| Increased alanine aminotransferase | 2 (12.5) | 0 | 2 (12.5) |
| Nausea | 2 (12.5) | 0 | 2 (12.5) |

**Table S6. Immune-related adverse event (IRAE) occurring in more than one patient by grade and Child-Pugh status.**

| Child Pugh A (n=10) | | | | Child Pugh B (n=6) | | |
|---|---|---|---|---|---|---|
| **Common IRAEs (n>1 in total cohort)** | | | | | | |
| **Immune-related adverse event, n (%)** | **Grade 1 or 2** | **Grade 3 or 4** | **Any grade** | **Grade 1 or 2** | **Grade 3 or 4** | **Any grade** |
| Rash | 6 (60) | 0 | 6 (60) | 3 (50) | 0 | 3 (50) |
| Increased aspartate aminotransferase | 3 (30) | 0 | 3 (30) | 0 | 2 (33.3) | 2 (33.3) |
| Diarrhea | 2 (20) | 0 | 2 (20) | 1 (16.7) | 0 | 1 (16.7) |
| Fatigue | 2 (20) | 1 (10) | 3 (30) | 0 | 0 | 0 |
| Myalgia | 2 (20) | 0 | 2 (20) | 1 (16.7) | 0 | 1 (16.7) |
| Mucositis | 2 (20) | 0 | 2 (20) | 0 | 1 (16.7) | 1 (16.7) |
| Constipation | 1 (10) | 0 | 1 (10) | 1 (16.7) | 0 | 1 (16.7) |
| Decreased appetite | 2 (20) | 0 | 2 (20) | 0 | 0 | 0 |
| Dry eye | 2 (20) | 0 | 2 (20) | 0 | 0 | 0 |
| Dry skin | 0 | 0 | 0 | 2 (33.3) | 0 | 2 (33.3) |
| Emesis | 1 (10) | 0 | 1 (10) | 1 (16.7) | 0 | 1 (16.7) |
| Hyperbilirubinemia | 1 (10) | 0 | 1 (10) | 0 | 1 (16.7) | 1 (16.7) |
| Hyponatremia | 1 (10) | 1 (10) | 2 (20) | 0 | 0 | 0 |
| Increased alanine aminotransferase | 1 (10) | 0 | 1 (10) | 1 (16.7) | 0 | 1 (16.7) |
| Nausea | 1 (10) | 0 | 1 (10) | 1 (16.7) | 0 | 1 (16.7) |
| **Individual patients with Gr3/4 IRAEs** | | 2 (20) | | | 3 (50) | |

**Table S7. Clinical responses overall and by dose level (DL).**

| | DL –1 (n=11) | DL 1 (n=5) | Total (n=16) |
|---|---|---|---|
| Complete response (CR) | 0 | 0 | 0 |
| Partial response (PR) | 1 (9.1) | 0 | 1 (6.3) |
| Stable disease (SD) | 8 (72.7) | 0 | 8 (50) |
| Progressive disease (PD) | 2 (18.1) | 5 (100) | 7 (43.8) |
| Not evaluable (NE) | 0 | 0 | 0 |
| **Response rate, n (%)** | | | |
| Objective response rate (CR/PR) | 1 (9.1) | 0 | 1 (6.3) |
| Disease control rate (CR/PR/SD) | 9 (81.8) | 0 | 9 (56.3) |
| **Kaplan-Meier estimate of median duration of SD, months (95% confidence interval)** | | | |
| Progression-free survival (PFS) | 3.68 (2.83, NR) | 1.84 (1.58, NR) | 2.58 (2.01, 7.17) |
| Overall survival (OS) | 15.2 (10.03, NR) | 8.78 (5.95, NR) | 12.99 (8.65, 35.8) |
