# Supplementary material for: Phase II trial with nivolumab and sorafenib in HCC identified enrichment of immunosuppressive monocytes in patients with Child-Pugh B liver dysfunction
Source: JHEP Rep. 2026 Mar 12;8(5):101817. doi: 10.1016/j.jhepr.2026.101817 (PMC13101793; doi:10.1016/j.jhepr.2026.101817)
Supplement: Multimedia component 2 [file mmc2.docx]

**JHEP Reports**

**CTAT methods**

Tables for a “Complete, Transparent, Accurate and Timely account” (CTAT) are now mandatory for all revised submissions. The aim is to enhance the reproducibility of methods.

- Only include the parts relevant to your study
- Refer to the CTAT in the main text as ‘Supplementary CTAT Table’
- Do not add subheadings
- Add as many rows as needed to include all information
- Only include one item per row

**If the CTAT form is not relevant to your study, please outline the reasons why:**

|  |
| --- |

- 1. **Antibodies- see 1.4 for CITEseq antibodies used**

| **Name** | **Citation** | **Supplier** | **Cat no.** | **Clone no.** |
| --- | --- | --- | --- | --- |
|  |  |  |  |  |

- 1. **Cell lines – n/a**

| **Name** | **Citation** | **Supplier** | **Cat no.** | **Passage no.** | **Authentication test method** |
| --- | --- | --- | --- | --- | --- |
|  |  |  |  |  |  |

- 1. **Organisms – n/a**

| **Name** | **Citation** | **Supplier** | **Strain** | **Sex** | **Age** | **Overall n number** |
| --- | --- | --- | --- | --- | --- | --- |
|  |  |  |  |  |  |  |

- 1. **Sequence based reagents**

| **Name** | **Sequence** | **Supplier** |
| --- | --- | --- |
| 0005 anti-human CD80 | ACGAATCAATCTGTG | BioLegend |
| 0006 anti-human CD86 | GTCTTTGTCAGTGCA | BioLegend |
| 0007 anti-human CD274 (B7-H1, PD-L1) | GTTGTCCGACAATAC | BioLegend |
| 0008 anti-human CD273 (B7-DC, PD-L2) | TCAACGCTTGGCTAG | BioLegend |
| 0009 anti-human CD275 (B7-H2, ICOSL) | GTGCATTCAACAGTA | BioLegend |
| 0014 anti-mouse/human CD11b | TGAAGGCTCATTTGT | BioLegend |
| 0021 anti-human CD252 (OX40L) | TTTAGTGATCCGACT | BioLegend |
| 0022 anti-human CD137L (4-1BB Ligand) | ATTCGCCTTACGCAA | BioLegend |
| 0023 anti-human CD155 (PVR) | ATCACATCGTTGCCA | BioLegend |
| 0024 anti-human CD112 (Nectin-2) | AACCTTCCGTCTAAG | BioLegend |
| 0026 anti-human CD47 | GCATTCTGTCACCTA | BioLegend |
| 0027 anti-human CD70 | CGCGAACATAAGAAG | BioLegend |
| 0028 anti-human CD30 | TCAGGGTGTGCTGTA | BioLegend |
| 0031 anti-human CD40 | CTCAGATGGAGTATG | BioLegend |
| 0032 anti-human CD154 | GCTAGATAGATGCAA | BioLegend |
| 0033 anti-human CD52 | CTTTGTACGAGCAAA | BioLegend |
| 0034 anti-human CD3 | CTCATTGTAACTCCT | BioLegend |
| 0046 anti-human CD8 | GCGCAACTTGATGAT | BioLegend |
| 0047 anti-human CD56 | TCCTTTCCTGATAGG | BioLegend |
| 0050 anti-human CD19 | CTGGGCAATTACTCG | BioLegend |
| 0052 anti-human CD33 | TAACTCAGGGCCTAT | BioLegend |
| 0053 anti-human CD11c | TACGCCTATAACTTG | BioLegend |
| 0054 anti-human CD34 | GCAGAAATCTCCCTT | BioLegend |
| 0056 anti-human CD269 (BCMA) | CAGATGATCCACCAT | BioLegend |
| 0057 anti-human B2-microglobulin | CAGCCCGATTAAGGT | BioLegend |
| 0058 anti-human HLA-A,B,C | TATGCGAGGCTTATC | BioLegend |
| 0060 anti-human CD90 (Thy1) | GCATTGTACGATTCA | BioLegend |
| 0061 anti-human CD117 (c-kit) | AGACTAATAGCTGAC | BioLegend |
| 0062 anti-human CD10 | CAGCCATTCATTAGG | BioLegend |
| 0063 anti-human CD45RA | TCAATCCTTCCGCTT | BioLegend |
| 0064 anti-human CD123 | CTTCACTCTGTCAGG | BioLegend |
| 0066 anti-human CD7 | TGGATTCCCGGACTT | BioLegend |
| 0070 anti-human/mouse CD49f | TTCCGAGGATGATCT | BioLegend |
| 0071 anti-human CD194 (CCR4) | AGCTTACCTGCACGA | BioLegend |
| 0072 anti-human CD4 | TGTTCCCGCTCAACT | BioLegend |
| 0073 anti-mouse/human CD44 | TGGCTTCAGGTCCTA | BioLegend |
| 0081 anti-human CD14 | TCTCAGACCTCCGTA | BioLegend |
| 0083 anti-human CD16 | AAGTTCACTCTTTGC | BioLegend |
| 0085 anti-human CD25 | TTTGTCCTGTACGCC | BioLegend |
| 0087 anti-human CD45RO | CTCCGAATCATGTTG | BioLegend |
| 0088 anti-human CD279 (PD-1) | ACAGCGCCGTATTTA | BioLegend |
| 0089 anti-human TIGIT (VSTM3) | TTGCTTACCGCCAGA | BioLegend |
| 0090 Mouse IgG1, k isotype Ctrl | GCCGGACGACATTAA | BioLegend |
| 0091 Mouse IgG2a, k isotype Ctrl | CTCCTACCTAAACTG | BioLegend |
| 0092 Mouse IgG2b, k isotype Ctrl | ATATGTATCACGCGA | BioLegend |
| 0095 Rat IgG2b, k Isotype Ctrl | GATTCTTGACGACCT | BioLegend |
| 0100 anti-human CD20 | TTCTGGGTCCCTAGA | BioLegend |
| 0101 anti-human CD335 (NKp46) | ACAATTTGAACAGCG | BioLegend |
| 0102 anti-human CD294 (CRTH2) | TGTTTACGAGAGCCC | BioLegend |
| 0123 anti-human CD326 (Ep-CAM) | TTCCGAGCAAGTATC | BioLegend |
| 0124 anti-human CD31 | ACCTTTATGCCACGG | BioLegend |
| 0127 anti-Human Podoplanin | GGTTACTCGTTGTGT | BioLegend |
| 0129 anti-human CD140b (PDGFRb) | CAATGGTTCACTGCC | BioLegend |
| 0134 anti-human CD146 | CCTTGGATAACATCA | BioLegend |
| 0135 anti-human CD324 (E-Cadherin) | ATCCTTCTCCCTTTC | BioLegend |
| 0136 anti-human IgM | TAGCGAGCCCGTATA | BioLegend |
| 0138 anti-human CD5 | CATTAACGGGATGCC | BioLegend |
| 0139 anti-human TCR gd | CTTCCGATTCATTCA | BioLegend |
| 0140 anti-human CD183 (CXCR3) | GCGATGGTAGATTAT | BioLegend |
| 0141 anti-human CD195 (CCR5) | CCAAAGTAAGAGCCA | BioLegend |
| 0142 anti-human CD32 | GCTTCCGAATTACCG | BioLegend |
| 0143 anti-human CD196 (CCR6) | GATCCCTTTGTCACT | BioLegend |
| 0144 anti-human CD185 (CXCR5) | AATTCAACCGTCGCC | BioLegend |
| 0145 anti-human CD103 (Integrin alpha E) | GACCTCATTGTGAAT | BioLegend |
| 0146 anti-human CD69 | GTCTCTTGGCTTAAA | BioLegend |
| 0147 anti-human CD62L | GTCCCTGCAACTTGA | BioLegend |
| 0148 anti-human CD197 (CCR7) | AGTTCAGTCAACCGA | BioLegend |
| 0149 anti-human CD161 | GTACGCAGTCCTTCT | BioLegend |
| 0151 anti-human CD152 (CTLA-4) | ATGGTTCACGTAATC | BioLegend |
| 0152 anti-human CD223 (LAG-3) | CATTTGTCTGCCGGT | BioLegend |
| 0153 anti-human KLRG1 (MAFA) | CTTATTTCCTGCCCT | BioLegend |
| 0154 anti-human CD27 | GCACTCCTGCATGTA | BioLegend |
| 0155 anti-human CD107a (LAMP-1) | CAGCCCACTGCAATA | BioLegend |
| 0156 anti-human CD95 (Fas) | CCAGCTCATTAGAGC | BioLegend |
| 0158 anti-human CD134 (OX40) | AACCCACCGTTGTTA | BioLegend |
| 0159 anti-human HLA-DR | AATAGCGAGCAAGTA | BioLegend |
| 0160 anti-human CD1c | GAGCTACTTCACTCG | BioLegend |
| 0162 anti-human CD64 | AAGTATGCCCTACGA | BioLegend |
| 0163 anti-human CD141 (Thrombomodulin) | GGATAACCGCGCTTT | BioLegend |
| 0164 anti-human CD1d | TCGAGTCGCTTATCA | BioLegend |
| 0165 anti-human CD314 (NKG2D) | CGTGTTTGTTCCTCA | BioLegend |
| 0166 anti-human CD66b | AGCTGTAAGTTTCGG | BioLegend |
| 0167 anti-human CD35 | ACTTCCGTCGATCTT | BioLegend |
| 0168 anti-human CD57 | AACTCCCTATGGAGG | BioLegend |
| 0169 anti-human CD366 (Tim-3) | TGTCCTACCCAACTT | BioLegend |
| 0170 anti-human CD272 (BTLA) | GTTATTGGACTAAGG | BioLegend |
| 0171 anti-human/mouse/rat CD278 (ICOS) | CGCGCACCCATTAAA | BioLegend |
| 0174 anti-human CD58 (LFA-3) | GTTCCTATGGACGAC | BioLegend |
| 0175 anti-human CD96 (TACTILE) | TGGCCTATAAATGGT | BioLegend |
| 0176 anti-human CD39 | TTACCTGGTATCCGT | BioLegend |
| 0177 anti-human CD178 (Fas-L) | CCGGTCCTCTGTATT | BioLegend |
| 0179 anti-human CX3CR1 | AGTATCGTCTCTGGG | BioLegend |
| 0180 anti-human CD24 | AGATTCCTTCGTGTT | BioLegend |
| 0181 anti-human CD21 | AACCTAGTAGTTCGG | BioLegend |
| 0185 anti-human CD11a | TATATCCTTGTGAGC | BioLegend |
| 0186 anti-human IgA | AAGATGTCCGAGCAA | BioLegend |
| 0187 anti-human CD79b (Ig-beta) | ATTCTTCAACCGAAG | BioLegend |
| 0188 anti-human CD66a/c/e | GGGACAGTTCGTTTC | BioLegend |
| 0189 anti-human CD244 (2B4) | TCGCTTGGATGGTAG | BioLegend |
| 0196 anti-human CD235ab | GCTCCTTTACACGTA | BioLegend |
| 0205 anti-human CD206 (MMR) | TCAGAACGTCTAACT | BioLegend |
| 0206 anti-human CD169 (Sialoadhesin, Siglec-1) | TACTCAGCGTGTTTG | BioLegend |
| 0207 anti-human CD370 (CLEC9A/DNGR1) | CTGCATTTCAGTAAG | BioLegend |
| 0208 anti-human XCR1 | AAGACGCATGTCAAC | BioLegend |
| 0214 anti-human/mouse integrin beta7 | TCCTTGGATGTACCG | BioLegend |
| 0215 anti-human CD268 (BAFF-R) | CGAAGTCGATCCGTA | BioLegend |
| 0217 anti-human CD54 | CTGATAGACTTGAGT | BioLegend |
| 0218 anti-human CD62P (P-Selectin) | CCTTCCGTATCCCTT | BioLegend |
| 0224 anti-human TCR ab | CGTAACGTAGAGCGA | BioLegend |
| 0245 anti-human CD106 | TCACAGTTCCTTGGA | BioLegend |
| 0246 anti-human CD122 (IL-2RB | TCATTTCCTCCGATT | BioLegend |
| 0247 anti-human CD267 (TACI) | AGTGATGGAGCGAAC | BioLegend |
| 0352 anti-human FceRIA | CTCGTTTCCGTATCG | BioLegend |
| 0353 anti-human CD41 | ACGTTGTGGCCTTGT | BioLegend |
| 0355 anti-human CD137 (4-1BB) | CAGTAAGTTCGGGAC | BioLegend |
| 0356 anti-human CD254 (TRANCE, RANKL) | TCCGTGTTAGTTTGT | BioLegend |
| 0358 anti-human CD163 | GCTTCTCCTTCCTTA | BioLegend |
| 0359 anti-human CD83 | CCACTCATTTCCGGT | BioLegend |
| 0360 anti-human CD357 (GITR) | ACCTTTCGACACTCG | BioLegend |
| 0362 anti-human CD309 (VEGFR2) | TTCACGCAGTAAGAT | BioLegend |
| 0363 anti-human CD124 (IL-4Ra) | CCGTCCTGATAGATG | BioLegend |
| 0366 anti-human CD184 (CXCR4) | TCAGGTCCTTTCAAC | BioLegend |
| 0367 anti-human CD2 | TACGATTTGTCAGGG | BioLegend |
| 0368 anti-human CD226 (DNAM-1) | TCTCAGTGTTTGTGG | BioLegend |
| 0369 anti-human CD29 | GTATTCCCTCAGTCA | BioLegend |
| 0370 anti-human CD303 (BDCA-2) | GAGATGTCCGAATTT | BioLegend |
| 0371 anti-human CD49b | GCTTTCTTCAGTATG | BioLegend |
| 0373 anti-human CD81 (TAPA-1) | GTATCCTTCCTTGGC | BioLegend |
| 0374 anti-human CD98 | GCACCAACAGCCATT | BioLegend |
| 0375 anti-human IgG Fc | CTGGAGCGATTAGAA | BioLegend |
| 0384 anti-human IgD | CAGTCTCCGTAGAGT | BioLegend |
| 0385 anti-human CD18 | TATTGGGACACTTCT | BioLegend |
| 0386 anti-human CD28 | TGAGAACGACCCTAA | BioLegend |
| 0387 anti-human TSLPR (TSLP-R) | CAGTCCTCTCTGTCA | BioLegend |
| 0389 anti-human CD38 | TGTACCCGCTTGTGA | BioLegend |
| 0390 anti-human CD127 (IL-7Ra) | GTGTGTTGTCCTATG | BioLegend |
| 0391 anti-human CD45 | TGCAATTACCCGGAT | BioLegend |
| 0392 anti-human CD15 (SSEA-1) | TCACCAGTACCTAGT | BioLegend |
| 0393 anti-human CD22 | GGGTTGTTGTCTTTG | BioLegend |
| 0394 anti-human CD71 | CCGTGTTCCTCATTA | BioLegend |
| 0395 anti-human B7-H4 | TGTATGTCTGCCTTG | BioLegend |
| 0396 anti-human CD26 | GGTGGCTAGATAATG | BioLegend |
| 0397 anti-human CD193 (CCR3) | ACCAATCCTTTCGTC | BioLegend |
| 0399 anti-human CD204 | TAGCGAGCCAGATGT | BioLegend |
| 0400 anti-human CD144 (VE-Cadherin) | TCCACTCATTCTGTA | BioLegend |
| 0402 anti-human CD1a | GATCGTGTTGTGTTA | BioLegend |
| 0406 anti-human CD304 (Neuropilin-1) | GGACTAAGTTTCGTT | BioLegend |
| 0407 anti-human CD36 | TTCTTTGCCTTGCCA | BioLegend |
| 0420 anti-human CD158 (KIR2DL1/S1/S3/S5) | TATCAACCAACGCTT | BioLegend |
| 0437 anti-mouse/human CD207 | CGATTTGTATTCCCT | BioLegend |
| 0576 anti-human CD49d | CCATTCAACTTCCGG | BioLegend |
| 0577 anti-human CD73 (Ecto-5'-nucleotidase) | CAGTTCCTCAGTTCG | BioLegend |
| 0581 anti-human TCR Va7.2 | TACGAGCAGTATTCA | BioLegend |
| 0582 anti-human TCR Vd2 | TCAGTCAGATGGTAT | BioLegend |
| 0583 anti-human TCR Vg9 | AAGTGATGGTATCTG | BioLegend |
| 0584 anti-human TCR Va4-Ja18 (iNKT cell) | AACTTCTGTGGTAGC | BioLegend |
| 0590 anti-human CD305 (LAIR1) | ATTTCCATTCCCTGT | BioLegend |
| 0591 anti-human LOX-1 | ACCCTTTACCGAATA | BioLegend |
| 0592 anti-human CD158b (KIR2DL2/L3, NKAT2) | GACCCGTAGTTTGAT | BioLegend |
| 0594 anti-human CD133 | GTAAGACGCCTATGC | BioLegend |
| 0597 anti-human CD209 (DC-SIGN) | TCACTGGACACTTAA | BioLegend |
| 0599 anti-human CD158e1 (KIR3DL1, NKB1) | GGACGCTTTCCTTGA | BioLegend |
| 0600 anti-human CD158f (KIR2DL5) | AAAGTGATGCCACTG | BioLegend |
| 0801 anti-human CD337 (NKp30) | AAAGTCACTCTGCCG | BioLegend |
| 0802 anti-human CD336 (NKp44) | GGGCAATTAGCGAGT | BioLegend |
| 0828 anti-human CD307d (FcRL4) | CGATTTGATCTGCCT | BioLegend |
| 0829 anti-human CD307e (FcRL5) | TCACGCAGTCCTCAA | BioLegend |
| 0830 anti-human CD319 (CRACC) | AGTATGCCATGTCTT | BioLegend |
| 0831 anti-human CD138 (Syndecan-1) | GTATAGACCAAAGCC | BioLegend |
| 0845 anti-human CD99 | ACCCGTCCCTAAGAA | BioLegend |
| 0853 anti-human CLEC12A | CATTAGAGTCTGCCA | BioLegend |
| 0856 anti-Tau Phospho (Thr181) | CTCGTTTGTAGCAAT | BioLegend |
| 0863 anti-human CD257 (BAFF, BLYS) | CAGAGCACCCATTAA | BioLegend |
| 0867 anti-human CD94 | CTTTCCGGTCCTACA | BioLegend |
| 0870 anti-human CD150 (SLAM) | GTCATTGTATGTCTG | BioLegend |
| 0894 anti-human Ig light chain k | AGCTCAGCCAGTATG | BioLegend |
| 0895 anti-mouse/human Mac-2 (Galectin-3) | GATGCAATTAGCCGG | BioLegend |
| 0896 anti-human CD85j (ILT2) | CCTTGTGAGGCTATG | BioLegend |
| 0897 anti-human CD23 | TCTGTATAACCGTCT | BioLegend |
| 0898 anti-human Ig light chain lambda | CAGCCAGTAAGTCAC | BioLegend |
| 0899 anti-human HLA-A2 | GAACATTTCCGACAA | BioLegend |
| 0900 anti-human CD198 (CCR8) | AGCCCGGATGTATTT | BioLegend |
| 0901 anti-human GARP (LRRC32) | AGGTATGGTAGAGTA | BioLegend |
| 0902 anti-human CD328 (Siglec-7) | CTTAGCATTTCACTG | BioLegend |
| 0908 anti-human TCR Vb13.1 | TTATGGACGTATGGT | BioLegend |
| 0920 anti-human CD82 | TCCCACTTCCGCTTT | BioLegend |
| 0944 anti-human CD101 (BB27) | CTACTTCCCTGTCAA | BioLegend |
| 0985 anti-human CD360 (IL-21R) | GAGGATGATGCCATG | BioLegend |
| 1046 anti-human CD88 (C5aR) | GCCGCATGAGAAACA | BioLegend |
| 1047 anti-human HLA-F | GCAACTCTCCTACCT | BioLegend |
| 1048 anti-human NLRP2 | ACGCTTGTGTTAGTT | BioLegend |
| 1049 anti-human CD289 (TLR9) | CTATGATGCTGCATG | BioLegend |
| 1051 anti-human Podocalyxin | GAGCCGGTATAATGC | BioLegend |
| 1052 anti-human CD224 | CTGATGAGATGTCAG | BioLegend |
| 1055 anti-c-Met | GCTGCTGCGATTTGA | BioLegend |
| 1056 anti-human CD258 (LIGHT) | ACTTCCCTGTAGAAA | BioLegend |
| 1057 anti-human DR3 (TRAMP) | GAGTTCCCTCAGTTC | BioLegend |

- 1. **Biological samples**

| **Description** | **Source** | **Identifier** |
| --- | --- | --- |
| Peripheral blood mononuclear cells | University of California San Francisco | B-01, B-02, B-03, B-04, B-05, B-06, B-07, B-08, B-09, B-10, B-11, B-12, B-13, B-14, B-15, B-17, B-18, B-21, B-22, B-23 |
| Resected Surgical Tissue | University of California San Francisco | A-12, A-18, A-19, A-20, A-23, A-24, A-30 |

- 1. **Deposited data**

| **Name of repository** | **Identifier** | **Link** |
| --- | --- | --- |
| NCBI GEO | GSE318420 | https://www.ncbi.nlm.nih.gov/geo/query/acc.cgi?acc=GSE318420 |

- 1. **Software**

| **Software name** | **Manufacturer** | **Version** |
| --- | --- | --- |
| CellRanger | 10x Genomics | 7.0.0 |
| Freemuxlet | https://github.com/statgen/popscle | 0.1 |
| Scrublet | https://github.com/AllonKleinLab/scrublet | 0.2.1 |
| Scanpy | https://github.com/theislab/Scanpy | 1.6.0 |
| Harmony | https://github.com/immunogenomics/harmony | 0.0.10 |
| Memento | https://github.com/yelabucsf/scrna-parameter-estimation | 0.1.2 |
| GSEApy | https://github.com/zqfang/GSEApy | 1.1.7 |

- 1. **Other (*e.g*. drugs, proteins, vectors etc.) – n/a**

|  |  |  |
| --- | --- | --- |
|  |  |  |

- 1. **Please provide the details of the corresponding methods author for the manuscript:**

| Bridget Keenan, address: 550 16th St., San Francisco, CA 94143, email: bridget.keenan@ucsf.edu; Robin K. Kelley, address: 550 16th St., San Francisco, CA 94143, email: katie.kelley@ucsf.edu. |
| --- |

**2.0 Please confirm for randomised controlled trials all versions of the clinical protocol are included in the submission. These will be published online as supplementary information.**

| All protocol and informed consent versions are included in the supplement. |
| --- |
