# Supplementary material for: Phase II trial with nivolumab and sorafenib in HCC identified enrichment of immunosuppressive monocytes in patients with Child-Pugh B liver dysfunction
Source: JHEP Rep. 2026 Mar 12;8(5):101817. doi: 10.1016/j.jhepr.2026.101817 (PMC13101793; doi:10.1016/j.jhepr.2026.101817)
Supplement: Multimedia component 4 [file mmc4.pdf]

Research article

Phase II trial with nivolumab and sorafenib in HCC identified enrichment of immunosuppressive monocytes in patients with Child-Pugh B liver dysfunction

Authors

Bridget P. Keenan, Zenghua Fan, Bryan Khuong Le, …, Alan P. Venook, Edward J. Kim, Robin K. Kelley

Correspondence

bridget.keenan@ucsf.edu (B.P. Keenan), katie.kelley@ucsf.edu (R.K. Kelley).

Graphical abstract

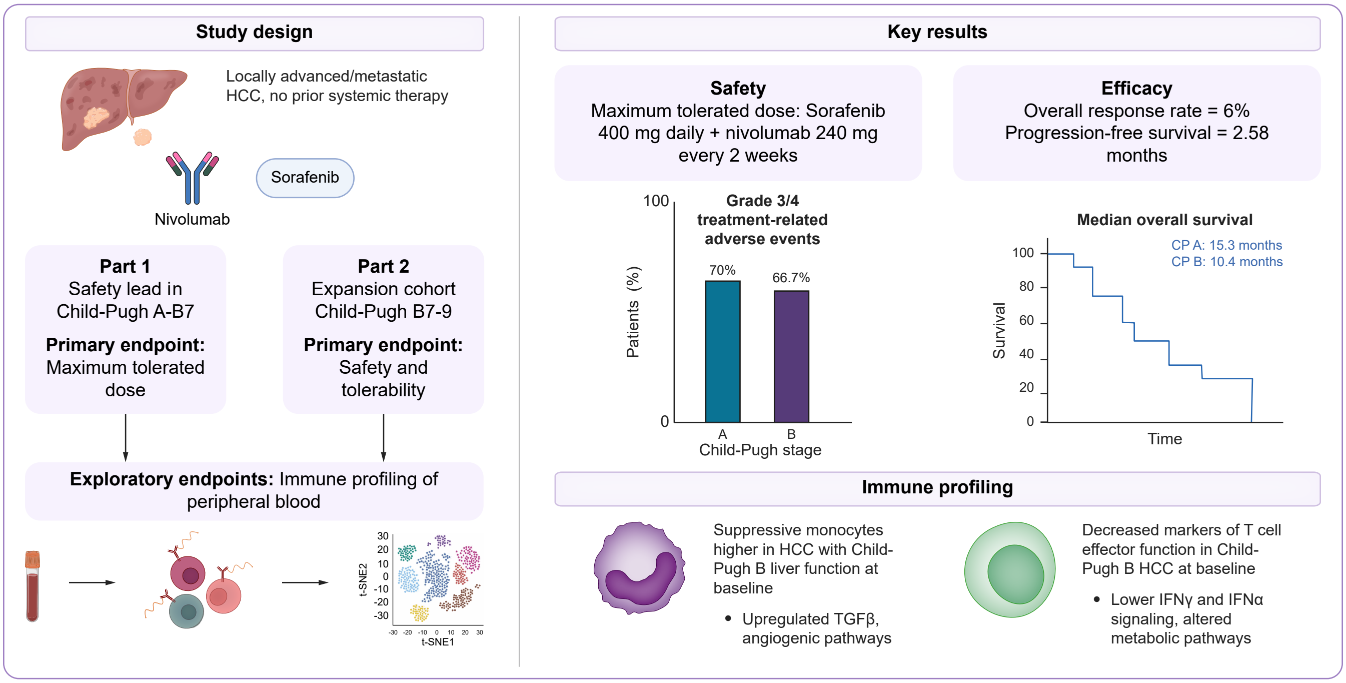

Highlights:

- The combination of sorafenib and nivolumab showed acceptable safety in Child-Pugh A/B HCC.
- Grade ≥3 TRAEs occurred in 69% of enrolled patients.
- The objective response rate of 6% did not meet the threshold for further exploration.
- Patients with Child-Pugh B disease had more suppressive $CD14^+$ monocytes at baseline.
- Myeloid cells should be targeted in advanced liver disease to overcome resistance.

Impact and implications:

This study addresses the crucial need for effective therapies for patients with advanced HCC and compromised liver function, a population historically excluded from many clinical trials. The findings suggest that, although the combination of sorafenib and nivolumab demonstrated acceptable safety across different liver function subgroups, there was a low response rate overall. Importantly, the discovery of distinct immune profiles, particularly an increase in suppressive immune cells in patients with worse liver function, provides insights into potential mechanisms of resistance to immunotherapy. These results are crucial for understanding how we can improve the treatment of advanced HCC, especially in patients with impaired liver function.

https://doi.org/10.1016/j.jhepr.2026.101817

© 2026 The Authors. Published by Elsevier B.V. on behalf of European Association for the Study of the Liver (EASL). This is an open access article under the CC BY license (http://creativecommons.org/licenses/by/4.0/). JHEP Reports, 2026, 8, 1–15

JHEP|Reports

Phase II trial with nivolumab and sorafenib in HCC identified enrichment of immunosuppressive monocytes in patients with Child-Pugh B liver dysfunction

Bridget P. Keenan[1,2,3,*], Zenghua Fan[1,2,3], Bryan Khuong Le[1], Quincy Harris[1], Jocelin Chen[1], Matthew Clark[1,2,3], Averey Lea[1,2,3], Li Zhang[1,2,3,4], Alexander Cheung[1,2,3], Frances Lara[5], John D. Gordan[1,2], Paige Bracci[1,4], Spencer C. Behr[1,6], Lawrence Fong[1,2,3,7], Alan P. Venook[1,2], Edward J. Kim[5], Robin K. Kelley[1,2,*]

JHEP Reports **2026**. vol. 8 | 1–15

Check for updates

Background & Aims: Immune checkpoint inhibition (ICI) and anti-angiogenic therapies are active in hepatocellular carcinoma (HCC), although patients with impaired hepatic function have worse outcomes.

Methods: We conducted an open-label phase II clinical trial to assess the safety and efficacy of the multikinase inhibitor, sorafenib, combined with nivolumab, in patients with advanced HCC and varying liver function. In a Part 1 safety lead-in, we investigated the maximum-tolerated dose (MTD) of the combination in patients with Child-Pugh A or B7. In Part 2, we enrolled patients with Child-Pugh B7-9 HCC with the primary endpoint of grade ≥3 treatment-related adverse event (TRAE) incidence. Exploratory endpoints included immunological biomarkers.

Results: Overall, 25 patients were consented and 16 eligible patients enrolled. In Part 1, dose-limiting toxicity occurred in one of six patients in Dose Level -1, and two of five patients in Dose Level 1; Dose Level -1 was determined to be the MTD. In total, 69% of patients experienced a grade ≥3 TRAE, with similar distribution for patients with Child-Pugh A and B (70%, 95% CI: 0.35–0.93 *vs.* 66.7%, 95% CI: 0.22–0.96). The objective response rate was 6% and median overall survival was 12.99 months (Child-Pugh A: 15.26 and Child-Pugh B: 10.41). We found that patients with Child-Pugh B liver disease harbored more circulating suppressive $CD14^+$ monocytes at baseline compared with those with Child-Pugh A disease.

Conclusions: Although combination sorafenib and nivolumab demonstrated acceptable safety at the MTD in both Child-Pugh subgroups, the objective response rate was below the prespecified threshold to be declared worthy of further exploration. A distinct immune cell profile in patients with Child-Pugh B disease might define mechanisms of resistance and potential therapeutic targets in this population with unmet clinical need.

Clinical trial: NCT03439891.

© 2026 The Authors. Published by Elsevier B.V. on behalf of European Association for the Study of the Liver (EASL). This is an open access article under the CC BY license (http://creativecommons.org/licenses/by/4.0/).

Introduction

Hepatocellular carcinoma (HCC) is the third leading cause of cancer death worldwide and is increasing in incidence in the USA.[1–3] Most patients are diagnosed with, or progress to, advanced stages of disease requiring systemic therapies, although only a subset of patients experience durable benefit. Antiangiogenic therapies, including multikinase inhibition with agents such as sorafenib, lenvatinib, cabozantinib, and regorafenib, have been a mainstay of therapy for advanced HCC since the pivotal SHARP trial demonstrated improvement in overall survival (OS) with sorafenib compared with placebo.[4] Immune checkpoint inhibition (ICI) with monoclonal antibodies targeting programmed cell death protein 1 (PD-1) and programmed cell death ligand 1 (PD-L1), such as nivolumab, pembrolizumab, atezolizumab, and durvalumab, can achieve durable objective responses in 15–18% of patients but low rates of disease control overall.[5–8]

Combining antiangiogenic therapy with ICI holds the potential to augment the immune response through modulation of both the tumor immune microenvironment and microvasculature.[9–11] Multikinase inhibitors, the targets of which include vascular endothelial growth factor (VEGF) receptor isoforms, have been shown to differentially inhibit immunosuppressive immune cell populations, including regulatory T cells and myeloid cells, as well as inducing PD-L1 expression on immune cells, factors that might favor immune responses on ICI.[12–17] Inhibition of VEGF or its receptors could also foster an immune responsive microenvironment through normalization of microvasculature.[18,19]

* Corresponding authors. Address: University of California, San Francisco (UCSF), 550 16th Street, San Francisco, CA 94143, USA.
E-mail addresses: bridget.keenan@ucsf.edu (B.P. Keenan), katie.kelley@ucsf.edu (R.K. Kelley).
https://doi.org/10.1016/j.jhepr.2026.101817

The benefit of combining ICI with VEGF-targeted therapies in HCC has been established in multiple phase III clinical trials.[20–22] The combination of atezolizumab plus bevacizumab was demonstrated to be superior to sorafenib in the IMbrave150 trial, with median OS of 19.2 months for atezolizumab plus bevacizumab *vs.* 13.4 months for sorafenib (hazard ratio [HR] 0.66).[20,23] Likewise, the combination of VEGR receptor (VEGFR)-2 inhibition with rivoceranib and the anti-PD-1 ICI, camrelizumab, showed significant prolongation of OS and progression-free survival (PFS) in the CARES-310 trial, but was accompanied by higher rates of grade 3 and 4 toxicity.[21] In the COSMIC-312 trial, a regimen of cabozantinib plus atezolizumab also achieved significant prolongation in PFS over standard sorafenib, but failed to improve OS, with higher rates of toxicity.[22] Collectively, these studies validate the activity of ICI combined with antiangiogenic agents in HCC but underscore the potential for higher rates of toxicity.

Despite these recent advances in treatment options, an ongoing challenge remains to characterize the safety and efficacy of systemic therapies in patients with greater degrees of underlying liver dysfunction. Patients with more severe underlying liver dysfunction represent a substantial proportion of advanced HCC (40–50%) but historically have been excluded from prospective clinical trials because of poor prognosis.[24,25] Retrospective data and small prospective studies suggest that patients with worse degrees of liver dysfunction, including patients with Child-Pugh scores of B or albumin–bilirubin (ALBI) grade 2 or higher, have lower rates of objective response and shorter median OS on systemic therapies, including ICI, compared with patients with preserved liver function, although rates of immune-related adverse events (IRAE) are similar.[5,26–32] Although there are poorer overall outcomes compared with Child-Pugh A, a subset of patients with Child-Pugh B cirrhosis achieve robust responses on ICI-based therapy that exceed the rate and duration of response for sorafenib monotherapy, underscoring the importance of identifying mechanisms of response and resistance in this vulnerable population with poor prognosis.[28,33–36]

We developed this pilot study to examine the safety and preliminary efficacy of the combination of sorafenib with nivolumab in patients with advanced stages of HCC and varying degrees of liver dysfunction, along with exploring changes in peripheral blood immune cell profiles on treatment. At the inception of this study, there was no established first-line combination immunotherapy regimen for advanced HCC. With mid-study regulatory approvals of combination immunotherapy regimens in patients with preserved liver function, the focus of the study was amended to advanced HCC with Child-Pugh B liver dysfunction, an area of ongoing unmet clinical need. Beyond clinical endpoints of safety and preliminary efficacy, we explored blood samples using high-dimensional single cell transcriptomics and proteomics to understand immune responses to the treatment combination, along with the contributions of liver disease etiology and severity.

Patients and methods

Trial design

This was a single-arm, multicenter, investigator-initiated phase II trial with safety lead-in to evaluate the safety and efficacy of sorafenib and nivolumab for the treatment of patients with advanced unresectable HCC, without prior systemic therapy, with Child-Pugh A or B liver function. Participants were enrolled at two sites: University of California, San Francisco (UCSF) in San Francisco, CA, USA and University of California, Davis (UCD) in Davis, CA, USA. This study was conducted in accordance with the Declaration of Helsinki and after approval by the UCSF Cancer Center and UCD Institutional Review Boards. Both study sites were monitored by the UCSF Data and Safety Monitoring Committee (DSMC) according to the NCI-approved Data and Safety Monitoring Plan for multicenter trials. All patients provided written informed consent. The study was registered at Clinicaltrials.gov as NCT03439891. Part 1 of the pilot study was designed to first determine the maximum-tolerated dose (MTD) of sorafenib in combination with nivolumab in an Escalation Cohort with 3 + 3 design. Given the mid-study regulatory approvals of other combination immunotherapy regimens for patients with advanced HCC and Child-Pugh A liver function,[7,20] the Part 2 Expansion Cohort of this study was amended to restrict eligibility to Child-Pugh B liver function to address the unmet clinical need in this population. The study was discontinued early in December 2022 because of the changing treatment landscape resulting in slow accrual.

Patients

Patients with advanced or unresectable HCC,[37] measurable by RECIST 1.1, who had not been treated with previous systemic therapy were eligible. In Part 1, patients with a Child-Pugh score of A or B7 were eligible; in Part 2, eligibility required a Child-Pugh score of B7–9 liver function. Patients were required to be age 18 years or older and have ECOG performance status of 0–1 at the time of enrollment. Patients with HBV infection were allowed with appropriate antiviral prophylaxis, but patients with both active HBV and HCV or HDV were excluded. Patients with uncontrolled ascites, hepatic encephalopathy within the past 6 months, or upper gastrointestinal bleeding within the past 12 months were excluded. Treatment with systemic steroids or other immunosuppression, active autoimmune disease, and uncontrolled high blood pressure (>140/90 mmHg at study entry) were also exclusions. Treatment with previous chemoembolization, radioembolization, local ablative therapies, or hepatic radiation was not allowed within 4 weeks of enrollment, and hepatic resection was not permitted within 6 weeks of enrollment.

Treatment

In the Part 1 dose escalation portion of the study, patients were treated with sorafenib at a starting dose of 400 mg orally once daily (Dose Level -1) or 400 mg orally twice daily (Dose Level 1) plus nivolumab 240 mg intravenously every 2 weeks. In the Part 2 dose expansion portion of the study, patients were treated at the MTD, which was sorafenib 400 mg orally once daily and nivolumab 240 mg intravenously every 2 weeks. Treatment was continued until progression, unacceptable toxicity, withdrawal of consent, or the study end.

Assessments

Adverse events were graded and recorded throughout the study according to NCI CTCAE Version 4.03. For Part 1 of the

study, the dose-limiting toxicity (DLT) window was 28 days (1 cycle). Subjects had to receive two doses of nivolumab and at least 75% of sorafenib doses within 28 days (1 cycle), or experience a qualifying DLT event, to be evaluable for DLT. DLTs were defined as clinically significant toxicities that were at least possibly treatment related and met the criteria listed in the study protocol. Dose escalation was determined in a standard 3 + 3 design. Cross-sectional imaging with multiphase computed tomography (CT) or magnetic resonance imaging (MRI) of abdomen plus pelvis and CT scan of chest with/without or without contrast was performed at screening, then every 8 weeks until end of treatment. Tumor responses were measured using RECIST 1.1. The evaluable population for the secondary efficacy endpoints included all patients who had received at least one complete cycle of therapy (4 weeks) and had restaging imaging for response assessment or were removed for clinical progression.

Endpoints

In the Part 1 safety lead-in, the primary endpoint was establishing the MTD of the combination of sorafenib and nivolumab in patients with Child-Pugh A–B7 liver function. In Part 2, the primary endpoint was the safety of the combination in patients with Child-Pugh B liver function. Secondary endpoints included the following: safety and tolerability of the combination overall; rate of IRAEs for the combination overall and in patients with Child-Pugh B liver function; objective response rate (ORR) by RECIST 1.1; and duration of response (DOR); PFS, and OS for the overall study and in patients with Child-Pugh B liver function. An ORR of 15% was prespecified as the target threshold for efficacy to consider further study of the combination.

Research biospecimens

Peripheral blood mononuclear cells (PBMCs) were obtained from patients pre- and on-treatment for the subset of patients treated at the UCSF study site (Table 1; Table S1). Blood samples were processed using Ficoll (Cytiva, Marlborough, MA, USA); after centrifugation, the PBMC layer was isolated and cryopreserved in cell media with human serum and DMSO. Previously frozen PBMCs were thawed using media containing RPMI, heat-inactivated sterile filtered FBS, penicillin–streptomycin, non-essential amino acids, sodium pyruvate, and L-glutamine. For tissue correlation with PBMC findings, tumor samples were collected from a separate cohort of patients undergoing resection and consented under the UCSF Hepatobiliary Tissue Bank and Registry (IRB #12-09576) (n = 7 patients total; Table S2). For some resections, adjacent liver tissue was also available and processed in parallel. Samples were digested in RPMI containing Collagenase I & II

Table 1. Demographic characteristics of patients enrolled in the Phase II clinical trial of combination sorafenib and nivolumab.

	Part and sorafenib starting DL			
	DL -1 (Part 1)	DL 1 (Part 1)	DL -1 (Part 2)	Overall
Demographic	n = 6	n = 5	n = 5	n = 16
Median age, years (SD)	64 (4.1)	67 (10.3)	66 (6.6)	66 (7.3)
Male, n (%)	5 (83.3)	3 (60)	5 (100)	13 (81.3)
Race, n (%)				
White	3 (50)	1 (20)	4 (80)	8 (50)
Black or African-American	1 (16.7)	0	0	1 (6.3)
Asian	2 (33.3)	4 (80)	1 (20)	7 (43.8)
American Indian or Alaska Native	0	0	0	0
Ethnicity				
Non-Hispanic/Latinx	6 (100)	4 (80)	3 (60)	13 (81.3)
Hispanic/Latinx	0	1 (20)	2 (40)	3 (18.8)
Viral status				
HBV+*	3 (50)	4 (80)	2 (40)	9 (56.3)
HCV+*	3 (50)	1 (20)	2 (40)	6 (37.5)
Non-viral	1 (16.7)	1 (20)	2 (40)	4 (25)
ECOG at enrollment				
0	5 (83.3)	1 (20)	2 (40)	8 (50)
1	1 (16.7)	4 (80)	3 (60)	8 (50)
Child-Pugh score at enrollment				
A5	4 (66.7)	2 (40)	0	6 (37.5)
A6	2 (33.3)	2 (40)	0	4 (25)
B7	0	1 (20)	3 (60)	4 (25)
B9	0	0	2 (40)	2 (12.5)
ALBI grade at enrollment				
1	2 (33.3)	0	0	2 (12.5)
2	4 (66.7)	4 (80)	2 (40)	10 (62.5)
3	0	1 (20)	3 (60)	4 (25)
BCLC				
B	1 (16.7)	1 (20)	2 (40)	4 (25)
C	5 (83.3)	4 (80)	3 (60)	12 (75)
Alpha-fetoprotein ≥400 ng/ml, n (%)	1 (16.7)	2 (40)	2 (40)	5 (31.3)
Extrahepatic disease	5 (83.3)	5 (100)	2 (40)	12 (75)
Vascular invasion	3 (50)	1 (20)	4 (80)	8 (50)

ALBI, albumin–bilirubin; BCLC, Barcelona Clinic Liver Cancer; DL, dose level.
*Some patients were both HBVcAb+ and HCV antibody positive, but were HBsAg negative.

(0.1 mg/ml, Sigma-Aldrich, St. Louis, MO, USA) and DNAse I, minced, and digested for 1 h using the GentleMACS system (Miltenyi Biotec, Bergisch Gladbach, Germany). Isolation of live cells (and, in some cases, $CD45^+$ cells following sorting for live cells) was performed using MACS LS columns (Miltenyi Biotec).

Single cell RNA sequencing/CITEseq

We then combined 1.1×10^5 cells from each PBMC sample into staining buffer (1 mmol/L EDTA and 2% heat-inactivated FBS in calcium/magnesium-free Dulbecco PBS; Thermo Fisher Scientific, Waltham, MA, USA) and stained with one pooled cocktail containing a custom panel of 197 Total-Seq C antibody–oligonucleotide conjugates (BioLegend, San Diego, CA, USA) as per standard protocols (Supplementary CTAT Table) following pre-incubation with TruStain FcX (Fc Receptor Blocking Solution, BioLegend). Droplet-based single cell RNA sequencing (scRNAseq) was performed using the 10X Genomics Chromium Single Cell 5' Reagent Kits v2, according to the manufacturer's instructions. 10X Genomics 5′ v2 Library Construction kits for feature barcoding (protein antibodies) and TCR amplification were used to generate libraries as per the manufacturer's instructions. scRNAseq of tumor samples was completed on fresh material with 10X 5’ v1 kits. All sequencing was performed on an Illumina NovaSeq S4 sequencer with paired end 200 bp read length and 25,000 reads per droplet for gene expression libraries and 5,000 reads per cell for feature barcode and TCR libraries.

Bioinformatics

CellRanger (10X Genomics, Genome Build: GRCh38 3.0.0, Supplementary CTAT Table for all software versions) was used to align the raw sequencing data. The feature barcoding library sequences were aligned to a customized reference genome provided by BioLegend containing the oligonucleotide sequences corresponding to each antibody. Pooled samples were demultiplexed by a genotyping-free pipeline Freemuxlet to assign cells to each individual patient.[38] Doublet detection was run by Scrublet[39] using default parameters on each sample. We used the Scanpy[40] data analysis pipeline for preprocessing and analysis of scRNAseq data. We applied the following cutoffs for filtering high quality cells: <10% mitochondrial genes, >100 and <2,500 genes expressed per cell, and excluded platelets, red blood cells, and doublets. We filtered out ribosomal genes and genes detected in fewer than three cells. Following sequencing alignment, preprocessing, quality control, and doublet removal, we recovered 177,000 cells from all samples combined, corresponding to >3,000 cells per sample. We $\log_2$ plus one transformed and normalized the data to 10,000 counts per cell, regressed out the percentage of mitochondrial genes and number of gene counts, and scaled genes to unit variance. We performed batch correction using Harmony[41] and then performed k-nearest neighbor graph construction and clustering on gene expression data; for analysis of all immune cells, we clustered cells with a resolution of 1.0. We reclustered on myeloid or T cells individually, removing any contaminating cells (non-myeloid or non-T cell); for myeloid cells, we used a resolution of 0.6, whereas, for T cells, we used resolution 1.3. We processed protein data by normalization and $\log_2$ plus one transformation as for RNA. Differential gene expression analysis was performed by the package Memento,[42] where significant genes (p <0.01) were selected for pathway enrichment analysis by GSEA implemented by the prerank module from package GSEApy[43] and normalized enrichment scores <1 or >1 were selected for visualization. Similarity comparison of cell clusters between two datasets was performed by the pseudobulk method described previously,[44] in which gene expression was aggregated from the cell level to the sample level. Shared genes in both datasets were selected for the correlation calculation, while, in each dataset, the union of top 100 genes in each cell cluster was selected.

Statistical analysis

Part 1 of the study was designed as a safety lead-in. A standard 3 + 3 dose escalation was used to determine the MTD. In Part 2 of the study, patients with Child-Pugh B disease were enrolled at the MTD with the primary endpoint of safety as measured by rate of grade ≥3 TRAEs (CTCAE v. 4.03) assessed as at least possibly related to sorafenib, nivolumab, or the combination of therapies. An acceptable rate of grade ≥3 TRAEs was predefined as no more than 50% based upon the previously reported rate of grade ≥3 TRAE of 24.5% among patients with Child-Pugh B7–8 HCC treated with nivolumab in the CheckMate 040 Child-Pugh B cohort[33] and 28% of patients with Child-Pugh B7–9 HCC treated with nivolumab in a retrospective institutional cohort,[26] along with rates of grade 3–4 TRAE of 30–36% in patients with Child-Pugh B7–9 HCC treated with sorafenib in the GIDEON registry.[35] With an expected sample size of 12 patients with Child-Pugh B7–9 HCC treated in Part 2, the 95% CI for determining the rate of grade ≥3 TRAE if the actual rate was 50% would be (0.22–0.78). With an abbreviated sample size of six patients in the Child-Pugh B cohort in Part 2, the 95% CI would be (0.10–0.90) if the actual rate was 50%.

To annotate cells, we used the embedded SCANPY function to identify differentially expressed genes in each cluster compared with the union of the rest of the clusters, which uses the Benjamini–Hochberg method[45] to control the false discovery rate. For frequency proportions, weighted least squares was used to adjust for the number of cells sequenced in each individual and Benjamini–Hochberg method was used to adjust p values for multiple comparisons. To assess the correlations of the frequency of cell types with other cell types, ALBI score, Child-Pugh score, PFS, and IRAEs, we used Spearman's rank correlation coefficient. For the IRAE correlation analysis, each patient was categorized by the highest grade IRAE if they had multiple occurrences.

Results

Study population

Between March 2018 and May 2022, a total of 25 patients were screened and 16 patients were enrolled at the two study sites: UCSF (n = 15), UC Davis (n = 1) (Table 1 and Fig. 1). Eleven patients were enrolled in Part 1, and 5 in Part 2 before study discontinuation. The median age was 66 years (range: 50–82 years), and 81% of patients were men. The study population was representative of the patient population seen at the enrolling sites and was 50% White, 43.8% Asian and 6.3%

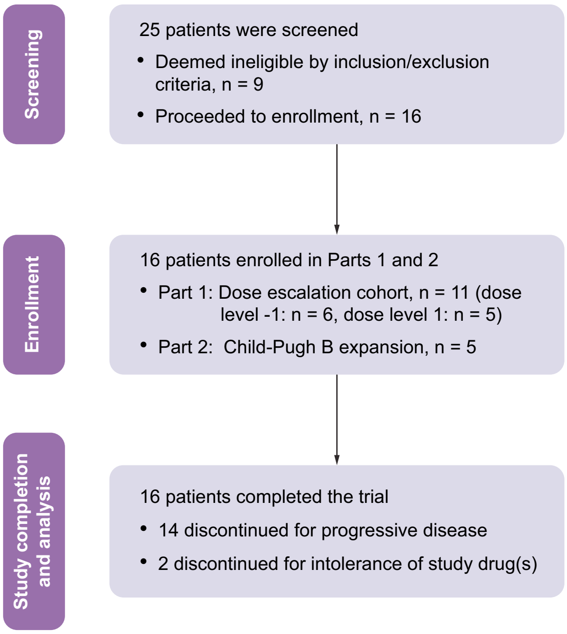

Fig. 1. CONSORT diagram outlining trial design and number of patients in each part.

Table 2. Treatment-related adverse events occurring in more than one patient by Grade.

Treatment-related adverse events occurring in n >1 patient, n (%)	Grade 1 or 2	Grade 3 or 4	Any grade
Rash	10 (62.5)	1 (6.3)	11 (68.8)
Increased aspartate aminotransferase	4 (36.4)	3 (18.8)	7 (43.8)
Diarrhea	6 (37.5)	0	6 (37.5)
Hypertension	5 (31.3)	1 (6.3)	6 (37.5)
Increased alanine aminotransferase	5 (31.3)	0	5 (31.3)
Myalgia	5 (31.3)	0	5 (31.3)
Palmar-plantar erythrodysesthesia syndrome	2 (12.5)	3 (18.8)	5 (31.3)
Dry skin	4 (36.4)	0	4 (36.4)
Hoarseness	4 (36.4)	0	4 (36.4)
Mucositis	3 (18.8)	1 (6.3)	4 (36.4)
Constipation	3 (18.8)	0	3 (18.8)
Decreased appetite/anorexia	3 (18.8)	0	3 (18.8)
Emesis	3 (18.8)	0	3 (18.8)
Fatigue	2 (12.5)	1 (6.3)	3 (18.8)
Hyperbilirubinemia	0	3 (18.8)	3 (18.8)
Nausea	3 (18.8)	0	3 (18.8)
Pruritus	3 (18.8)	0	3 (18.8)
Dyspepsia	2 (12.5)	0	2 (12.5)
Dry eye	2 (12.5)	0	2 (12.5)
Headache	2 (12.5)	0	2 (12.5)
Hypoalbuminemia	2 (12.5)	0	2 (12.5)
Hyponatremia	1 (6.3)	1 (6.3)	2 (12.5)
Weight loss	2 (12.5)	0	2 (12.5)

Black; 18.8% of patients identified as Hispanic/Latinx. Of the patients, 56.3% were HBV-positive, as defined by detectable HBsAg or HBV DNA and/or positive HBcAg, whereas 37.5% of patients had positive HCV antibody, and 25% had nonviral etiology. Liver function was characterized as a Child-Pugh score of A5 in 37.5%, A6 in 25%, B7 in 25%, and B9 in 12.5%, and ALBI grade 1 in 12.5%, 2 in 62.5%, and 3 in 25%. The Barcelona Clinic Liver Cancer (BCLC) stage was C in 75% and B in 25%. Other baseline patient and disease characteristics are summarized in Table 1.

Safety

In Part 1, we assessed the primary endpoint of MTD of sorafenib in combination with nivolumab in DLT-evaluable patients (n = 11). At the starting dose of sorafenib 400 mg once daily in combination with nivolumab 240 mg intravenously every 2 weeks (Dose Level -1), there was one patient with a DLT (grade 3 rash) and, therefore, the cohort was expanded to three more patients who did not experience DLTs. The dose was escalated to Dose Level 1 (sorafenib 400 mg twice a day in combination with nivolumab); two out of five patients treated experienced DLTs (one with grade 3 hyperbilirubinemia and ascites and another with grade 3 fatigue). Dose reductions or delays were required in one (20%) patient in Dose Level 1, and four (36.4%) in Dose Level -1. The MTD was determined to be Dose Level -1, with sorafenib 400 mg daily and nivolumab 240 mg intravenously every 2 weeks. Part 2 patients were treated at the MTD with the combination from Part 1.

We next evaluated TRAEs that occurred in more than one patient by grade for Parts 1 and 2 combined (n = 16; Table 2), as well as according to dose level (Table S3) and Child-Pugh status (Table S4). The rate of occurrence of any grade 3 or 4 TRAE in Child-Pugh A was 70% (7/10 patients; 95% CI: 0.35–0.93) and in Child-Pugh B, 66.7% (4/6 patients; 95% CI: 0.22–0.96). The most common grade 3–4 TRAEs were palmar-plantar erythrodysesthesia, elevated aspartate aminotransferase, and elevated bilirubin, each occurring in 3/16 patients (18.8%). IRAEs possibly or definitely related to nivolumab were evaluated by grade for Parts 1 and 2 combined (n = 16) (Table S5). The rate of occurrence of any grade 3 or 4 IRAE in Child-Pugh A was 20% (2/10 patients; 95% CI: 0.03–0.56), and in Child-Pugh B, 50% (3/6 patients; 95% CI: 0.12–0.88) (Table S6). Systemic steroids were initiated for treatment of possible IRAEs in 38% (6/16) patients and in all but one case, the steroids were initiated after the first cycle of therapy was completed (Cycle 2 and beyond). Two patients with elevated transaminases initially attributed as IRAEs and treated with steroids were subsequently adjudicated as being more likely to result from disease progression with extensive tumor burden rather than being immune related. Another case of grade 3 rash treated with steroids was adjudicated as being more likely to be related to sorafenib than to nivolumab, based on response to dose reduction in sorafenib and enabling rechallenge with nivolumab after discontinuation of steroids. In total, three of 16 patients (19%) had confirmed IRAE requiring steroids, with one event each of grade 3 keratoacanthoma, grade 2 mucositis, and grade 4 immune-related hepatitis requiring prolonged courses of steroids. The patient with grade 4 immune-related hepatitis later developed grade 5 sepsis because of bacteremia.

Efficacy

We evaluated the efficacy of the treatment combination across both dose levels due to the small sample size. Patients received a median of 4 cycles, with a range of 2-28 cycles administered. The ORR per RECIST 1.1 was 6.3%, with one patient with Child-Pugh A liver disease experiencing a partial response (PR) and no patients with Child-Pugh B liver disease with objective responses (Table 3). The disease control rate was 56.3% (nine of 16 patients), with four patients each with Child-Pugh A and B liver disease experiencing stable disease

Table 3. Clinical responses overall and by Child-Pugh status.

Characteristic	Child-Pugh A (n = 10)	Child-Pugh B (n = 6)	Total (n = 16)
CR	0	0	0
PR	1	0	1
SD	4	4	8
PD	5	2	7
NE	0	0	0
Response rate, n (%)			
ORR (CR/PR)	1 (10)	0	1 (6.3)
Disease control rate (CR/PR/SD)	5 (50)	4 (66.7)	9 (56.3)
Kaplan-Meier estimate of median duration of SD, months (95% CI)			
Progression-free survival	2.76 (1.91–NR)	2.58 (2.04–NR)	2.58 (2.01–7.17)
Overall survival	15.26 (8.65–NR)	10.41 (2.83–NR)	12.99 (8.65–35.8)

CR, complete response; NE, not evaluable; NR, non-response; ORR, objective response rate; PD, progressive disease; PR, partial response; SD, stable disease.

in addition to the patient with PR. For the one PR, the DOR was 9.73 months. Overall, the median PFS was 2.58 months (95% CI: 2.01–7.17), compared with 2.76 (95% CI: 1.91–NR) for Child-Pugh A and 2.58 (95% CI: 2.04–NR) for Child-Pugh B (Fig. 2A). The median OS was 12.99 months (95% CI: 8.65–35.8), compared with 15.26 (95% CI: 8.65–NR) for Child-Pugh A and 10.41 (95% CI: 2.83–NR) for Child-Pugh B (Fig. 2B). When clinical responses were analyzed by dose of sorafenib, patients treated with Dose Level -1 had longer PFS compared with patients in Dose Level 1 (3.68, 95% CI: 2.83–NR *vs.* 1.84, 95% CI: 1.58–NR months, p <0.001), and there was no significant difference in median OS (15.2, 95% CI: 10.03–NR *vs.* 8.78, 95% CI: 5.95–NR months; p = 0.932) (Table S7).

Correlative analysis

We explored the circulating immune response both at baseline and during combination treatment with sorafenib and nivolumab by performing scRNAseq with simultaneous surface proteome analysis (cellular indexing of transcriptomes and epitopes by sequencing; CITEseq) on PBMCs. For analysis of baseline immune profiles according to liver function status, we included five patients who had consented to the clinical trial but did not receive protocol therapy due to screen failure (Table S1) and one patient who was treated but only had PBMC samples available from baseline for a total baseline PBMC sample size of 20. For on-treatment immune responses, correlates of clinical benefit, and longitudinal sample analysis, we only evaluated samples from patients who had PBMCs available from at least the first two timepoints (n = 14). For purposes of the correlative data, given the small number of objective responses and recognizing that some patients who had prolonged disease stabilization with the combination regimen had clinical benefit, we defined disease control ('DC') as either a PR or stable disease with PFS of 6 months or longer (PFS6) (n = 2). Non-response ('NR') was defined as patients who had progressive disease as the best response or stable disease but did not meet PFS6 (n = 12).

Using the combined CITEseq protein and gene expression information, we identified the main immune cell types within the circulation of patients (Fig. 3A, Fig. S1A–C). We focused on immune changes 2–4 weeks after the first dose of combination therapy, based on previous studies suggesting that pharmacodynamic effects on immune cell profiles are evident within this timeframe[46,47] and based upon the prevalence of evaluable samples at this timepoint. Comparing from the baseline timepoint before treatment ('pre') to following 2–5 weeks of treatment ('post'), we examined changes in the frequency of circulating immune cell types (Fig. 3B). Although no formal statistical analysis was possible because of the small number

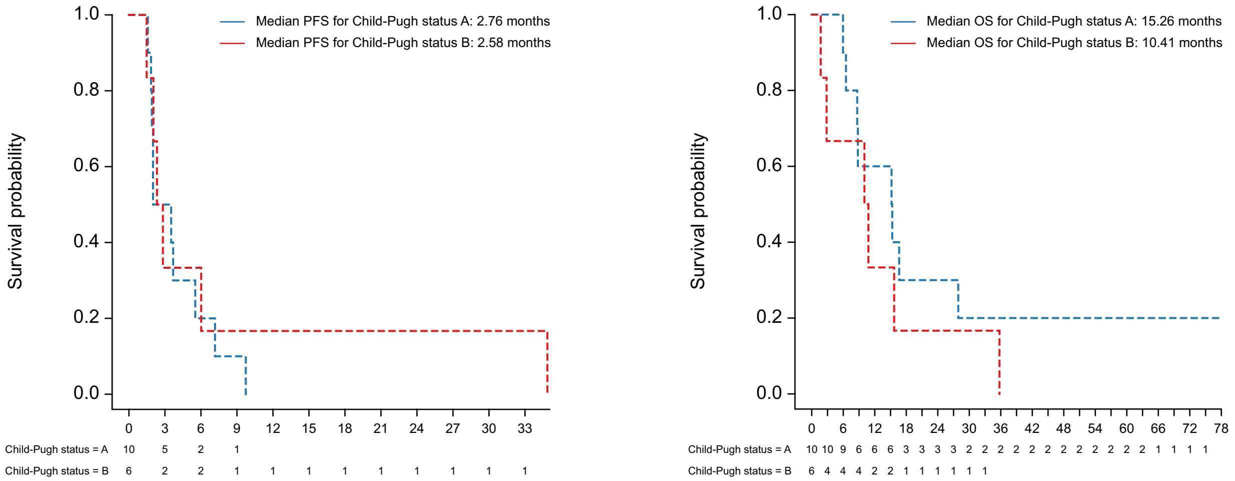

Fig. 2. PFS and OS by Child-Pugh liver function status. (A) PFS in months for patients with Child-Pugh A (n = 10) and B (n = 6) liver disease. (B) OS in months for patients with Child-Pugh A (n = 10) and B (n = 6) liver disease. OS, overall survival; PFS, progression-free survival.

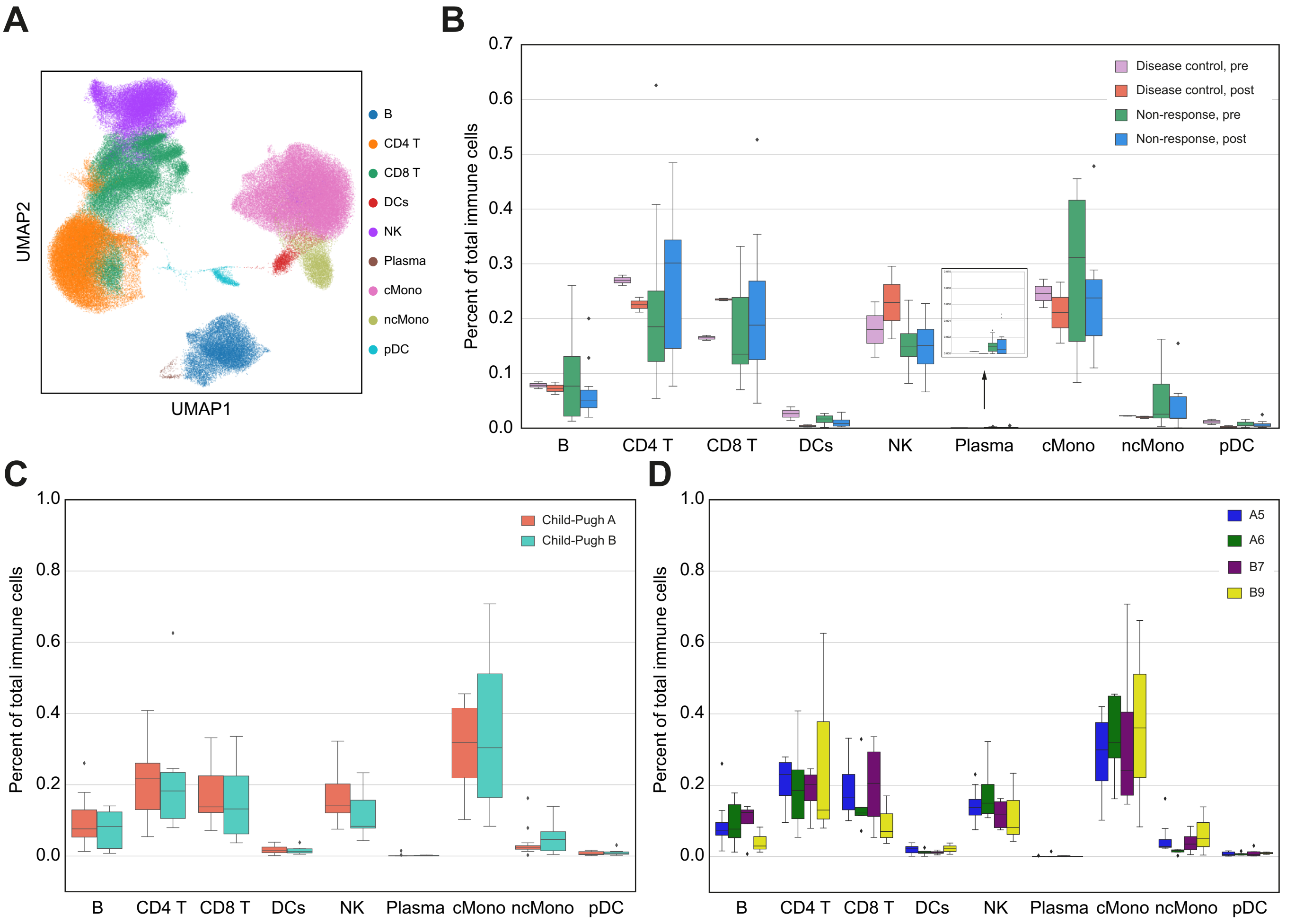

Fig. 3. Circulating immune cell responses at baseline and following sorafenib/nivolumab. (A) UMAP plot of PBMCs colored by cell type, where BB cells, CD4 T = CD4$^+$ T cells, CD8 T = CD8$^+$ T cells, and DCs = conventional dendritic cells. (B) Percentage of each cell type for patients with disease control (n = 2) or non-response (n = 12) before treatment (pre) and following one cycle of combination treatment (post). (C,D) Frequency of immune cell type at baseline (pre) for all patients (n = 20) by (C) Child-Pugh status (A [n = 13] *vs.* B [n = 7)]) and (D) Child-Pugh subscore (A5: n = 8, A6: n = 5, B7: n = 4, B9: n = 3). Boxes denote IQR, whereas bars denote 25% - 1.5xIQR and 75% + 1.5xIQR. Weighted least squares was used to account for different cell number per sample; multiple test adjustment with Benjamini-Hochberg for *p*-values. cMono, CD14$^+$ classical monocytes; ncMono, CD16$^+$ non-classical monocytes; NK, natural killer cells; plasma, plasma cells; PBMC, peripheral blood mononuclear cells; pDC, plasmacytoid dendritic cells; UMAP, Uniform Manifold Approximation and Projection.

of patients with DC, we observed that the patients with DC had an increase in CD8$^+$ T cells and natural killer (NK) cells, and decreased CD4$^+$ T cells, dendritic cells (both conventional [cDC] and plasmacytoid [pDC] dendritic cells), and classical monocytes following sorafenib/nivolumab treatment (effect sizes: 0.6, 1.11, 1.35, 1.54, and 1.84, respectively), whereas patients with NR appeared to have more static immune cell frequencies over time.

We next evaluated the immune response by liver disease status and etiology because these clinical factors have both been previously associated with the response to immunotherapy.[28,42] Comparing at the baseline timepoint, there were no differences in the circulating immune cell frequencies between virally associated (n = 14) and non-viral (n = 6) HCC (Fig. S1D). Furthermore, within subgroups of virally associated HCC, there were no differences between HCV *vs.* HBV, HBV *vs.* non-viral, or HCV *vs.* non-viral, although this was an exploratory analysis because of the small number of patients with each disease association (Fig. S1E). There were also no differences in the proportions of general types of circulating immune cell at baseline by stage of liver disease (Fig. 3C,D).

We previously identified a population of novel circulating CD14$^+$ monocytes with increased markers of chemotaxis ('CD14$_{CTX}$') that could induce CD4$^+$ T cell paralysis and was associated with ICI resistance in biliary tract cancer (BTC), another primary liver cancer,[46] and hypothesized that these might also exist within the circulation of patients with HCC. In PBMC samples from patients enrolled in the current study, we subclustered on myeloid cells and identified CD14$_{CTX}$ as well as other populations found in BTC (CD14$_{APC}$: CD14$^+$ monocytes characterized by antigen processing and presentation machinery and associated with ICI response; CD14$_{IFL:}$ inflammatory CD14$^+$ monocytes more frequently observed in healthy donors than patients with BTC; and CD14$_{ISG:}$ CD14$^+$ monocytes with an interferon response gene signature), as well as cDC, pDC, and non-classical CD16$^+$ monocytes (Fig. S2A–D). We also identified a population of DC-like CD14$^+$ monocytes that had gene signatures overlapping those of cDCs but expressed CD14, not

previously identified in the peripheral blood of patients with BTC. Infrequent granulocytes and megakaryocytes were seen because of the processing of blood samples, which resulted in the removal of most granulocytic cells.

The frequencies of myeloid cell states appeared static with treatment in the NR group, whereas patients in the DC group had increased $CD14_{APC}$ and $CD14_{ISG,}$ and decreased $CD14_{CTX}$ with treatment (effect sizes: 7.15, 2.69, and 1.3, respectively), resulting in a different myeloid cell composition compared with NR at the post-treatment timepoint (Fig. 4A). Acknowledging the small number of patients with DC, we also analyzed these findings by PFS status, which could be measured in a continuous fashion, performing a correlation analysis of the frequency of each cell population at the post-treatment timepoint with the length of PFS in months. Although this was also an exploratory analysis, we found a positive correlation of PFS with $CD14_{APC}$ and a negative correlation with pDC, granulocytes, and $CD14_{CTX}$ (Fig. 4B). Analysis of differential gene expression by response status revealed that the $CD14^{+}$ monocytes in the DC group but not the NR group specifically upregulated IFNγ, IFNα, fatty acid metabolism, and glycolysis pathways, which might support productive immune responses, whereas the NR group upregulated IL-6/JAK/STAT3 signaling and oxidative phosphorylation, pathways characteristic of suppressive myeloid cell responses (Fig. S2E and F), and generally had fewer genes significantly modulated by combination treatment compared with the DC group (Fig. S2E).

In baseline blood samples from patients with Child-Pugh B liver function, there was a higher proportion of $CD14_{CTX}$, compared with Child-Pugh A (Fig. 4C). Notably, a myeloid subpopulation seen more frequently in healthy donors than in patients with BTC,[46] $CD14_{IFL}$, was higher in patients with HCC and Child-Pugh A liver function, suggesting that, in Child-Pugh A, circulating monocytes retain normal function (Fig. 4C). Evaluating the frequency of $CD14_{CTX}$ by Child-Pugh subscore (*i.e.* A5, A6, B7, or B9) before treatment (Fig. 4D) demonstrated a positive correlation between higher scores and the frequency of $CD14_{CTX}$ pretreatment ($R = 0.56$, $p = 0.011$), whereas there was a non-significant inverse correlation between the numeric Child-Pugh score and the frequency of $CD14_{IFL}$ ($R = -0.4$, $p = 0.082$).

We also evaluated a correlation by ALBI score (in which an increased score correlates with rising bilirubin, lower albumin, higher grade liver dysfunction, and worse prognosis[48]), finding a negative correlation with $CD14_{IFL}$ ($R = -0.49$, $p = 0.027$) and a non-significant positive correlation with $CD14_{CTX}$ ($R = 0.34$, $p = 0.14$), demonstrating that other measures of worsening liver disease correspond with a suppressive myeloid state. $CD14^{+}$ monocytes from patients with Child-Pugh B liver function had upregulation of genes involved in TGFβ and Wnt/β-catenin signaling, angiogenesis, epithelial–mesenchymal transition, and hypoxia (Fig. 4E,F), consistent with the higher frequency of suppressive $CD14^{+}$ states within Child-Pugh B compared with Child-Pugh A. Given the marked differences in myeloid cells by liver function score, we evaluated dynamics in myeloid cell frequencies with treatment within each Child-Pugh subset and found a trend toward increasing $CD14_{IFL}$ and $CD14_{APC}$ and decreasing $CD14_{CTX}$ following combination treatment, suggesting that there could be partial reversal of suppressive myeloid phenotype with this treatment (Fig. S2G). By comparison, the frequency of $CD14_{CTX}$ remained low in patients with Child-Pugh A liver function before and following treatment. There were no apparent differences in myeloid subpopulations between viral *vs.* nonviral HCC, or between HBV and HCV when compared individually (Fig. S2H and I).

Given the potential clinical significance of the myeloid cell populations in the blood, we evaluated for the presence of analogous myeloid cells within the tumor microenvironment of HCC. In prior parallel analyses in BTC, we previously found a macrophage correlate of the circulating $CD14_{CTX}$ population in biliary tract tumors which expressed high SPP1.[46] We performed scRNAseq on primary HCC tumors from resection specimens (n = 7, Table S2) obtained from a separate cohort of patients, some of which included adjacent liver tissue, and categorized the myeloid cell sub-populations (Fig. S3A–C). Intriguingly, we found many monocytic/macrophage populations that highly correlated with monocyte sub-types in the periphery (DC-like mono, $CD14_{APC}$-like, $CD14_{IFL}$-like, and $CD14_{ISG}$-like), as well as dendritic cells, myeloid-derived suppressor cells (MDSC) and Kupffer cells. There were several tumor-associated macrophage populations (SPP1-Mac1-5); SPP1-Mac4 had both the highest expression of SPP1 and correlation with $CD14_{CTX}$, and was largely found in tumor rather than adjacent liver tissue (Fig. S3D).

We next evaluated T cell subtypes beyond $CD4^{+}$ and $CD8^{+}$ T cells to determine whether there was a relationship between clinical factors and T cell populations, and between myeloid cell and T cell states. We identified 15 subpopulations of T cells, including several populations of $CD4^{+}$ and $CD8^{+}$ proliferating, effector, naïve, memory, and regulatory T (Treg) cells, and innate-like T cell populations, including $\gamma\delta$T cells, mucosal-associated invariant T (MAIT) cells, and iNKT cells (Fig. S4A–C). We also specifically identified a $CD4^{+}$ naïve-like population with high SOCS3 expression (CD4 SOCS3) and a $CD4^{+}$ T cell population with high expression of IFN response genes (CD4 IFN) as we had found in BTC. As with the myeloid cells, we first examined whether there was a treatment effect on T cell subpopulations and found, in an exploratory analysis, that effector T cells, including a $CD8^{+}$ Granzyme K^{+} T effector memory ($CD45RA^{-}CCR7^{-}$) subpopulation (CD8 Tem GZMK), $CD8^{+}$ Granzyme B^{+} T terminally differentiated effector memory re-expressing CD45RA cells ($CD45RA^{+}CCR7^{-}$) (CD8 Temra GZMB), and MAIT cells increased with treatment in the DC group compared with the NR group, as did Tregs (effect sizes 2.57, 0.92, 1.44, and 2.95, respectively) (Fig. 5A). Within the DC group, there was a decrease in $CD4^{+}$ central memory T cells (CD4 Tcm) and CD4 SOCS3 T cells (effect sizes 4.01 and 1.56, respectively). These findings aligned with the correlation analysis of PFS and cell frequencies, which showed a positive correlation with survival and CD8 Tem GZMK and MAIT cells and a negative correlation for survival and CD4 SOCS3 (Fig. 5B). Across all T cells in patients with DC, there was an upregulation of pathways and genes related to IFNα, IFNγ, reactive oxygen species, complement, and glycolysis, whereas in the NR group, there were few genes modulated with treatment (Fig. 5C). There were no apparent differences in T cell subtype frequency by viral status or Child-Pugh score (Fig. S4D–G). However, investigating gene expression profiles and pathway analysis revealed that $CD4^{+}$ and $CD8^{+}$ T cells in Child-Pugh B compared with Child-Pugh A had enhanced TGFβ signaling, angiogenesis, and Wnt/β-catenin signaling,

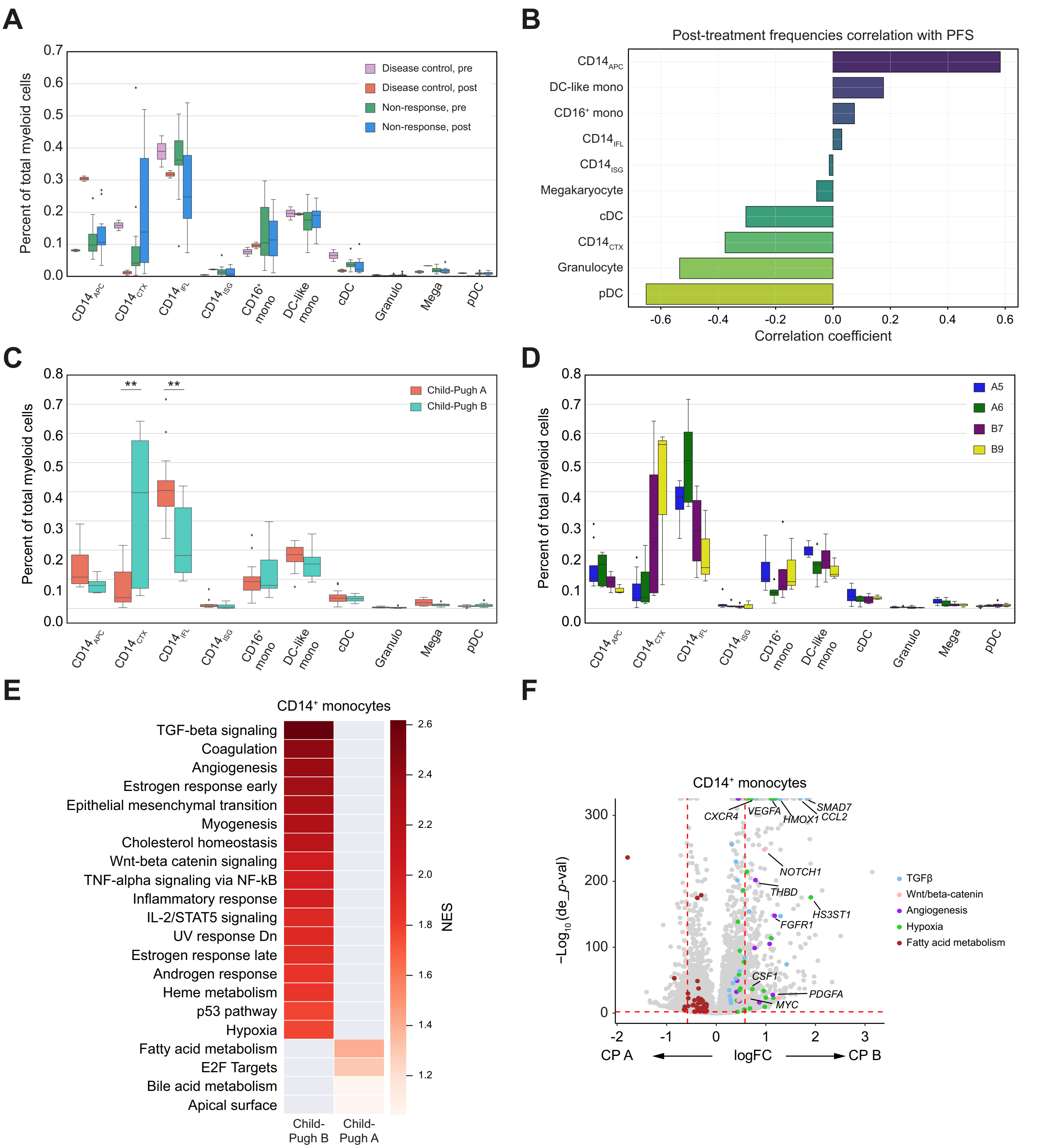

Fig. 4. Circulating myeloid cells at baseline and following sorafenib/nivolumab. (A) Percentage of each cell type out of total myeloid cells in circulation for patients with disease control (n = 2) or non-response (n = 12) before treatment (pre) and following one cycle of combination treatment (post). (B) Correlation coefficients for the frequency of each myeloid cell subtype at the post-treatment timepoint and PFS in days. (C,D) Frequency of myeloid cell type out of total circulating myeloid cells at baseline (pre) for all patients (n = 20) by (C) Child-Pugh status (A [n = 13] *vs.* B (n = 7) and (D) Child-Pugh subscore (A5: n = 8, A6: n = 5, B7: n = 4, B9: n = 3). *Adjusted p <0.05, **adjusted p <0.01. Boxes denote IQR, whereas bars denote 25% - 1.5xIQR and 75% + 1.5xIQR. (E) Pathways enriched within all $CD14^+$ monocytes ($CD14_{ISG}$, $CD14_{CTX}$, $CD14_{APC}$, $CD14_{IFL}$, and DC-like monocytes) in the Child-Pugh B compared with Child-Pugh A groups visualized for the top enriched pathways; heatmap corresponds to NES. (F) Differentially expressed genes in Child-Pugh B compared with Child-Pugh A disease, with genes of interest in the indicated pathways highlighted with the corresponding color dot. Weighted least squares was used to account for different cell number per sample; multiple testing adjustment with Benjamini-Hochberg for p-values. DC, dendritic cell; NES, normalized enrichment score; PFS, progression-free survival.

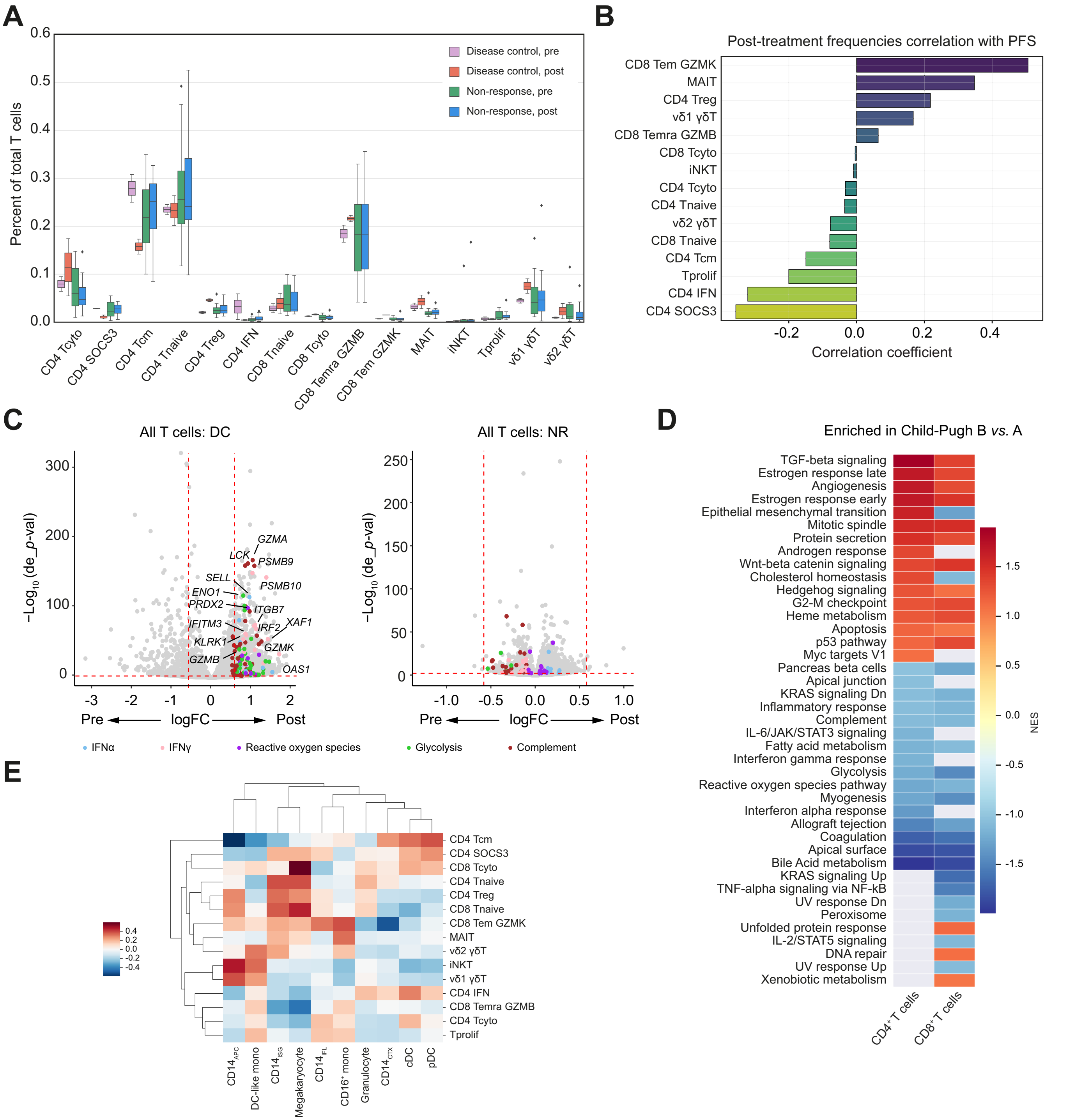

Fig. 5. Circulating T cells at baseline and following sorafenib/nivolumab. (A) Percentage of each cell type out of total T cells in circulation for patients with disease control (n = 2) or non-response (n = 12) before treatment (pre) and following one cycle of combination treatment (post). (B) Correlation coefficients for the frequency of each T cell subtype at the post-treatment timepoint and PFS in days. (C) Differentially expressed genes in post-treatment *vs.* pretreatment timepoints for all T cells in the DC and NR groups are plotted, with genes of interest in the indicated pathways highlighted with the corresponding color dot. (D) Pathways enriched within all CD4+ or CD8+ T cells in the Child-Pugh B compared with Child-Pugh A groups visualized for the top enriched pathways; heatmap corresponds to NES. Positive NES scores are pathways enriched in Child-Pugh B and negative scores are those enriched within Child-Pugh A. **(E)** Correlation of T cell (y-axis) and myeloid cell (x-axis) frequencies across the entire patient cohort. Legend shows correlation values demonstrated in heatmap format. Weighted least squares was used to account for different cell number per sample; multiple testing adjustment with Benjamini-Hochberg for *p*-values. DC, dendritic cell; NES, normalized enrichment scores; NR, non-response; PFS, progression-free survival; Tcm, central memory; Tcyto, cytotoxic T cells; Tem, effector memory; Temra, terminally differentiated re-expressing CD45RA effector memory; Tprolif, proliferating T cells.

and downregulated IFNα, IFNγ, and glycolysis pathways (Fig. 5D).

Finally, given the intriguing findings of myeloid and T cell dynamics induced by sorafenib and nivolumab, we evaluated correlations of cell frequencies between T cells and myeloid cells (Fig. 5E). CD8 Tem GZMK, the effector $CD8^+$ T cell population that was increased in patients with DC and improved PFS, positively correlated with more favorable $CD14^+$ populations, including significant direct correlations with $CD14_{IFL}$ (R = 0.4, p = 0.008), $CD14_{APC}$ (R = 0.2, p = 0.021), and $CD14_{ISG}$ (R = 0.39, p = 0.01), and was inversely correlated with $CD14_{CTX}$ (R = -0.52, p = 0.0004). Furthermore, pathways increased in T cells following combination immunotherapy were consistent with those seen in $CD14^+$ monocytes in cases with DC (IFNα, IFNγ, and glycolysis), suggesting that there is crosstalk between myeloid and T cell populations.

IRAEs have been correlated with ICI responses and survival in patients with cancer.[49,50] To test for a relationship in our cohort, we explored whether there was an immune signature characteristic of more severe IRAEs. We analyzed PBMCs at both the pretreatment timepoint for populations that might predict IRAE development, as well as on-treatment cell frequencies, which might indicate those immune cells involved in the emergence of IRAEs. The most highly correlated myeloid populations with IRAE severity were $CD14_{CTX}$ and pDC at baseline and granulocytes post treatment. Perhaps counter-intuitively, myeloid subtypes capable of antigen presentation, including $CD14_{APC}$ (pretreatment), cDC (post treatment), and DC-like monocytes (pre- and post treatment) were negatively correlated with IRAE grade (Fig. S5A). We also evaluated whether there was a T cell signature that was characteristic of severe IRAEs (Fig. S5B). MAIT cells correlated with severity both at the pre- and post-treatment timepoints, whereas CD4 T cm were more predictive at the pretreatment stage and CD4 Tnaive correlated with post-treatment IRAE severity.

Discussion

In this pilot study of the combination of sorafenib plus nivolumab in patients with advanced HCC, we aimed to determine the safety and MTD of this combination in patients with advanced HCC and varying degrees of liver dysfunction. The MTD was sorafenib 400 mg orally once daily in combination with nivolumab 240 mg intravenously every 2 weeks because of DLTs experienced at higher doses of sorafenib. TRAEs and IRAEs occurred at a similar frequency with combined sorafenib and nivolumab as historical rates for each drug as monotherapy.[33,36] The rates of grade 3–4 TRAE of 70% in the Child-Pugh A cohort and 66.7% in the Child-Pugh B cohort were similar to the rates of grade 3 or higher TRAE in other studies of multikinase inhibitors combined with ICI in patients with Child-Pugh A disease, notably 81% in CARES-310, 55% in COSMIC-312, and 63% in LEAP-002.[21,22,51] Likewise, the rate of TRAE in the Child-Pugh B subgroup was similar to that reported in other Child-Pugh B prospective studies. The rate of systemic steroid requirement for IRAEs was higher than expected for nivolumab as monotherapy in HCC but confounded by small sample size and overlapping attributions. Unfortunately, the ORR of 6% in this study did not meet the prespecified threshold for efficacy of 15% to warrant further investigation of the combination in patients with advanced HCC, regardless of liver function. Although the sample size was inadequately powered for efficacy assessment, the observed efficacy was insufficient to proceed with further study.

The expanding landscape of ICI-based combination therapies for advanced stages of HCC now includes the combination of atezolizumab plus bevacizumab, durvalumab plus a single dose of tremelimumab, and nivolumab plus ipilimumab, all of which prolong OS and achieve higher rates of objective response compared with sorafenib or lenvatinib as monotherapy.[7,20,23,52] All of these regimens have been studied in patients with preserved Child-Pugh A liver function, whereas the optimal treatment regimen for patients with greater degrees of liver dysfunction, including the large population of patients with Child-Pugh B cirrhosis, has not been well established. An important aspect of our study was the inclusion of both patients with Child-Pugh A disease and those with Child-Pugh B disease. In this small cohort, the DCR, PFS, and OS were similar between Child-Pugh A and Child-Pugh B, although the small sample size limited comparisons. Historical data for sorafenib and other multikinase inhibitors or VEGF-directed therapies have demonstrated worse outcomes for patients with Child-Pugh B liver function.[35,36,53,54] There are few prospective studies of ICI-based therapies in patients with Child-Pugh B HCC. In Cohort 5 of the Phase I/II CheckMate 040 study, nivolumab monotherapy achieved objective responses in 12% of patients, lower than the ORR of 20% in the Child-Pugh A cohort.[5,33] A retrospective study of patients with Child-Pugh A and B liver function treated with atezolizumab and bevacizumab demonstrated similar response rates and TRAEs.[34] ICI combination or monotherapy conferred a benefit in OS compared with best supportive care in patients with Child-Pugh B liver function (7.50 *vs.* 4.04 months).[55] After completion of our study, a pooled analysis of 10 studies showed a higher ORR for ICI-based combination regimens than for ICI as monotherapy (19% *vs.* 12%) in patients with HCC with Child-Pugh B liver function,[28] supporting the hypothesis that patients with Child-Pugh B liver function could also benefit from the combination approaches, although the absolute rates of objective response were lower than those observed in Child-Pugh A populations. Notably, the meta-analysis did not identify significant differences in TRAE rates, PFS, or OS according to Child-Pugh B subscore of 7 *vs.* 8/9, although sample sizes were limited.[28] Others have found the Child-Pugh score insufficient to convey the severity of liver dysfunction and advocated for the incorporation of individual factors related to liver decompensation (*i.e.* ascites, hepatic encephalopathy).[56] Given our small cohort, we were limited in our ability to ascertain the association of individual factors with outcomes and correlative data. In the context of these limited existing data, our trial of sorafenib combined with nivolumab supports that ICI combinations are feasible in carefully selected patients with Child-Pugh B HCC without excessive toxicity beyond that observed in Child-Pugh A populations.

Intriguingly, high-resolution scRNAseq of baseline peripheral blood samples obtained from patients enrolled on this trial showed a higher proportion of $CD14_{CTX}$ in patients with HCC and worse liver function, suggesting a mechanism for the lower ORRs observed for combination ICI therapies in HCC with greater degrees of hepatic dysfunction. In BTC, $CD14_{CTX}$ increased in frequency in the circulation of patients whose

tumors were resistant to ICI and induced 'immune-paralyzed' $CD4^+$ T cells characterized by *SOCS3* expression and low cytokine production and proliferative capacity.[46] Although we observed the presence of these $SOCS3^+$ $CD4^+$ T cells and that they were negatively correlated with PFS, there was not an increased frequency of this specific $CD4^+$ T cell subpopulation associated with Child-Pugh score in this HCC cohort; thus, there might be other mechanisms of suppression induced by these myeloid cells. Following treatment, we also observed a decrease in $CD14_{CTX}$ in the Child-Pugh B subgroup, suggesting that there is a partial reversal of myeloid-mediated suppression following treatment with combined ICI/sorafenib, compatible with other reports of sorafenib-induced myeloid cell differentiation,[57] and that perhaps other targeted therapies could improve on this reduction in suppressive monocytes.

In this small cohort, we did not observe meaningful differences in the baseline immune profile according to etiology of liver disease (viral *vs.* nonviral liver disease), and there were too few clinical responses to stratify by etiology. Although a meta-analysis suggested prolonged survival in virally associated compared with non-viral HCC,[58] other studies of contemporary ICI-based combinations have shown no appreciable differences in outcome according to liver disease etiology in HCC.[7,52,59,60] Given that metabolic dysfunction-associated steatotic liver disease is poised to become the most common cause of liver disease and HCC in the USA,[61] further studies are needed to characterize both the peripheral blood and tumor immune microenvironments according to etiology of liver disease and identify candidate biomarkers as well as therapeutic targets that might be unique to a specific etiology.

Using serial samples collected in this study, we also investigated how combined sorafenib and nivolumab affected the circulating immune response with multi-omic scRNAseq as a paradigm for elucidating mechanisms of response or resistance to other immunotherapy combination regimens in HCC. Although this was an exploratory analysis because of the limited number of patients with clinical benefit, we found that patients who had disease control had increased $CD8^+$ T cells following treatment; when we further evaluated T cell subsets, we found that the T cells most correlated with increased PFS were effector memory $CD8^+$ T cells expressing Granzyme K and MAIT cells, two T cell subsets that might contribute to both antitumor responses and the response to ICI.[62,63] Although the total frequency of circulating monocytes was not altered across the cohort, there were alterations in the frequency of monocyte subsets within the DC group, including a population previously associated with ICI response ($CD14_{APC}$) and a concomitant decrease in suppressive $CD14_{CTX}$ monocytes.[46] This aligned with enrichment of immunostimulatory pathways within monocytes in the DC group (*i.e.* IFN signaling) and immunosuppressive pathways in monocytes in the NR group (*i.e.* IL-6/JAK/STAT3 signaling). Furthermore, the $CD14^+$ monocytes in the DC group had upregulation of glycolysis (*vs.* oxidative phosphorylation in the NR group) with treatment, which has been demonstrated to be induced in the setting of productive immune responses.[64,65] Lastly, the correlated frequencies of immunostimulatory monocytes and effector T cells, and the upregulated antitumor pathways in both cell types suggest that there is a parallel shift in T cells and monocytes to a favorable immune program following combination immunotherapy. However, given the limited benefit of this regimen clinically, these immune alterations might only be induced in a minority of patients. Therefore, our findings point to mechanisms of immunotherapy response and resistance that must be more fully addressed by HCC regimens to improve clinical efficacy.

We also performed an exploratory analysis of immune signatures associated with the severity of IRAEs with intriguing findings. First, a myeloid population negatively associated with ICI response in this cohort and in BTC, $CD14_{CTX}$, was associated with worsening grade IRAEs. Given previous findings of IRAE association with response to ICI, this was surprising, although, in our cohort, we did not have a sufficient number to identify whether IRAEs correlated with response. Second, although antigen-presenting cells could initiate IRAEs by presenting host antigens, we found that myeloid populations with gene signatures related to antigen presentation (DC-like monocytes, cDCs, and $CD14_{APC}$) were negatively correlated with IRAE severity. Finally, we found that MAIT cells were associated with worsening IRAEs. Given that MAIT cells have been previously identified in both ulcerative and ICI-related colitis and have a role in regulating mucosal immunity, this could explain both their predictive and mechanistic role in IRAEs.[66,67]

Our study has several limitations, including small sample size and the resulting exploratory nature of the single cell analysis. The small sample size and slower-than-expected enrollment resulted, in part, from the changing treatment landscape for HCC with publication of the IMbrave150 trial soon after inception of our study, as well as the COVID-19 pandemic. In addition, we were only able to study the circulating immune responses because biopsy samples were beyond the scope of this investigator-initiated clinical trial. Thus, we correlated our PBMC results with tumor resection specimens from a separate cohort of patients with single cell methods and identified highly correlated monocyte/macrophage populations, including an $SPP1^+$ macrophage correlated with $CD14_{CTX.}$ In HCC, $SPP1^+$ macrophages inhabit 'tumor immune barriers' that prohibit immune cell infiltration into tumors, decreasing the efficacy of immunotherapy.[68] $SPP1^+$ macrophages have been shown to be suppressive of immunotherapy responses and associated with worse prognosis in other tumor types, demonstrating the clinical relevance of this population.[44,69,70]

Regardless of the limitations, the insights here are hypothesis generating for future studies of other treatment regimens now being used in the first-line setting in HCC, for which immune-based mechanisms of response and primary resistance are poorly understood, both in the overall population and specific to greater degrees of hepatic dysfunction. The safety and efficacy of ICI combination therapies in the setting of greater degrees of hepatic dysfunction remains an active area of ongoing clinical research, with multiple trials exploring safety and efficacy outcomes in patients with HCC and more advanced liver dysfunction (NCT05883644 and NCT06096779). Patients with worse immune dysfunction caused by liver disease could stand to benefit more from combination regimens that can target multiple mechanisms of immunotherapy resistance, including myeloid cells. Thus, the intersection of specific treatment regimens, safety in the context of liver dysfunction, and immunotherapy response is

an important area for future studies to optimize and personalize therapy in HCC.

Conclusions

Although the combination of sorafenib and nivolumab demonstrated acceptable safety in both Child-Pugh A and B subgroups in this study, the limited efficacy in this small cohort does not warrant further investigation. However, a distinct population of immunosuppressive myeloid cells was identified at baseline in the circulation in patients with Child-Pugh B hepatic dysfunction and warrants further study as a potential contributor to therapeutic resistance. Systemic therapy options for patients with greater degrees of liver dysfunction remain a significant unmet need, requiring further understanding of the distinct mechanisms of immune response and resistance in this population.

Affiliations

[1]*Helen Diller Family Comprehensive Cancer Center, University of California, San Francisco (UCSF), San Francisco, CA, USA;* [2]*Division of Hematology/Oncology, UCSF, San Francisco, CA, USA;* [3]*Cancer Immunotherapy Program, UCSF, San Francisco, CA, USA;* [4]*Department of Epidemiology and Biostatistics, UCSF, San Francisco, CA, USA;* [5]*UC Davis Comprehensive Cancer Center, Sacramento, CA, USA;* [6]*Department of Radiology & Biomedical Imaging, UCSF, San Francisco, CA, USA;* [7]*Immunotherapy Integrated Research Center, Fred Hutchinson Cancer Center, Seattle, WA, USA*

Abbreviations

ALBI, albumin–bilirubin; BCLC, Barcelona Clinic Liver Cancer; BTC, biliary tract cancer; cDC, conventional dendritic cells; CITEseq, cellular indexing of transcriptomes and epitopes by sequencing; cMono, $CD14^+$ classical monocytes; CT, computed tomography; DC, disease control; DL, dose level; DLT, dose-limiting toxicity; DOR, duration of response; HCC, hepatocellular carcinoma; HR, hazard ration; ICI, Immune checkpoint inhibition; IRAE, immune-related adverse event; MAIT, mucosal-associated invariant T cells; MRI, magnetic resonance imaging; MTD, maximum-tolerated dose; ncMono, $CD16^+$ non-classical monocytes; NE, not evaluable; NES, normalized enrichment score; NK, natural killer cells; NR, non-response; ORR, objective response rate; OS, overall survival; PBMC, peripheral blood mononuclear cells; PD-1, Programmed cell death protein 1; PD-L1, Programmed cell death ligand 1; PD, progressive disease; pDC = plasmacytoid dendritic cells; pDC, plasmacytoid dendritic cells; PFS, progression-free survival; PFS6, PFS of 6 months or longer; Plasma, plasma cells; PR, partial response; scRNAseq, single cell RNA sequencing; Tcm, central memory; Tcyto, cytotoxic T cells; Tem, effector memory; Temra, terminally differentiated re-expressing CD45RA effector memory; Tprolif, proliferating T cells; Treg, regulatory T cell; UMAP, Uniform Manifold Approximation and Projection; VEGF, vascular endothelial growth factor; VEGFR, vascular endothelial growth factor receptor.

Financial support

We acknowledge financial support from Bayer (to institution), Bristol Myers Squibb (nivolumab drug supply), Bili Project Foundation, Inc., and the imCORE Network through Genentech Inc (correlative studies). This work was also funded, in part, by research grants NIH K08CA290152, NIH 1K12CA260225-01, Damon Runyon Clinical Investigator Award, and UCSF Liver Center Pilot Award to BPK, and funding from the Shorenstein Family to BPK and APV.

Conflicts of interests

BPK has received research funding (to university) from Apexigen/Pyxis Oncology, Affini-T Therapeutics, Antengene, Innovative Cellular Therapeutics, Wugen, Astra Zeneca, Takeda, Roche/Genentech, Regeneron, and Boehringer Ingelheim; consulting or advisory fees (to self) from Regeneron, Agenus Inc., Cartography, and Arcellx; and travel expenses from Roche/Genentech. LZ has received consulting or advisory fees (to self) from Motive Medical Intelligence, Smith-Kettlewell Eye Research Institute, and Serna Bio. SCB has received consulting or advisory fees (to self) from Novartis and RiboScience. LF has received research funding (to university) from Roche/Genentech, Abbvie, Bavarian Nordic, Bristol Myers Squibb, Dendreon, Janssen, Merck, and Partner Therapeutics; and consulting or advisory fees (to self) from Actym, Astra Zeneca, Bioatla, Bristol Myers Squibb, Daiichi Sankyo, Immunogenesis, Innovent, Merck, Sutro, and Roche/Genentech. APV has received consulting or advisory fees (to self) from Agenus Bio. EJK has received research funding (to institution) from Bayer, Merck, EpicentRx, Astellas, Erytech, Fibrogen, NGM Biopharmaceuticals, Eureka, Genentech, GSK, VCN Biosciences, Incyte, Medimmune, Tizona, Celgene, Exelixis, Inc., Bristol Myers Squibb, Astra Zeneca, and Beigene; and consulting or advisory fees (to self): Eisai, Astra Zeneca, Seagen, Pfizer, and Relay Therapeutics. RKK has received research funding (to institution) from Agios, Astra Zeneca, Bayer, Bristol Myers Squibb, Compass Therapeutics, Eli Lilly, EMD Serono, Exelixis, Genentech/Roche, Merck, Partner Therapeutics, QED, Relay Therapeutics, Servier, Surface Oncology, Taiho, and Tyra Biosciences; consulting or advisory fees (to institution) from Astra Zeneca and Merck; and consulting or advisory fees (to self) from Compass Therapeutics, CVS Caremark, Elevar, GenMab, GSK, Jazz, J Pharma, Moderna, Regeneron, and Tyra Biosciences. The remaining authors declare no conflicts of interests that pertain to this work.

Please refer to the accompanying ICMJE disclosure forms for further details.

Authors' contributions

Conceptualization, funding acquisition, writing – original draft: BPK, RKK. Data curation: BPK, ZF, BKL, QH, JC, MC, AL, AC, FL, EJK, RKK. Formal analysis: BPK, ZF, LZ, PB, SCB, RKK. Investigation: BPK, ZF, BKL, QH, JC, MC, AL, AC, FL, JDG, APV, EJK, RKK. Methodology: BPK, ZF, MC, AL, RKK. Project administration: BPK, AC, RKK. Resources: BPK, ZF, JDG, LF, APV, EJK, RKK. Software: BPK, ZF, LZ. Supervision: BPK, LF, RKK. Visualization: BPK, ZF, LZ, RKK. Writing – review & editing: BPK, ZF, JDG, LF, RKK.

Data availability

scRNAseq data are available on NCBI GEO (GSE318420, Supplemental CTAT Table). Additional clinical trial data will be made available upon request.

Acknowledgments

We thank patients and caregivers for their participation; UCSF Gastrointestinal Medical Oncology providers involved in the screening, enrollment, and clinical care of these patients; the UCSF Biospecimen Resources Program for help with tissue acquisition; and the Institute for Human Genetics Core and the Center for Advanced Technologies at UCSF for help with sequencing. Figure 1 and the graphical abstract were created using BioRender (biorender.com).

Supplementary data

Supplementary data to this article can be found online at https://doi.org/10.1016/j.jhepr.2026.101817.

References

Author names in bold designate shared co-first authorship

[1] Petrick JL, Florio AA, Znaor A, et al. International trends in hepatocellular carcinoma incidence, 1978-2012. Int J Cancer 2020;147:317–330.

[2] Ryerson AB, Eheman CR, Altekruse SF, et al. Annual Report to the Nation on the Status of Cancer, 1975-2012, featuring the increasing incidence of liver cancer. Cancer 2016;122:1312–1337.

[3] Sung H, Ferlay J, Siegel RL, et al. Global Cancer Statistics 2020: GLOBOCAN estimates of incidence and mortality worldwide for 36 cancers in 185 countries. CA Cancer J Clin 2021;71:209–249.

[4] Llovet JM, Ricci S, Mazzaferro V, et al. Sorafenib in advanced hepatocellular carcinoma. New Engl J Med 2008;359:378–390.

[5] El-Khoueiry AB, Sangro B, Yau T, et al. Nivolumab in patients with advanced hepatocellular carcinoma (CheckMate 040): an open-label, non-comparative, phase 1/2 dose escalation and expansion trial. Lancet 2017;389:2492–2502.

[6] Finn RS, Ryoo BY, Merle P, et al. Pembrolizumab as second-line therapy in patients with advanced hepatocellular carcinoma in

KEYNOTE-240: a randomized, double-blind, phase III trial. J Clin Oncol 2020;38:193–202.
[7] Abou-Alfa GK, Lau G, Kudo M, et al. Tremelimumab plus durvalumab in unresectable hepatocellular carcinoma. NEJM Evid 2022;1. EVIDoa2100070.
[8] Lee MS, Ryoo BY, Hsu CH, et al. Atezolizumab with or without bevacizumab in unresectable hepatocellular carcinoma (GO30140): an open-label, multicentre, phase 1b study. Lancet Oncol 2020;21:808–820.
[9] Fukumura D, Kloepper J, Amoozgar Z, et al. Enhancing cancer immunotherapy using antiangiogenics: opportunities and challenges. Nat Rev Clin Oncol 2018;15:325–340.
[10] Yi M, Jiao D, Qin S, et al. Synergistic effect of immune checkpoint blockade and anti-angiogenesis in cancer treatment. Mol Cancer 2019;18:60.
[11] **Zhou Y**, **Liu Z**, Yu A, et al. Immune checkpoint inhibitor combined with antiangiogenic agent synergistically improving the treatment efficacy for solid tumors. Immunotargets Ther 2024;13:813–829.
[12] Cabrera R, Ararat M, Xu Y, et al. Immune modulation of effector CD4+ and regulatory T cell function by sorafenib in patients with hepatocellular carcinoma. Cancer Immunol Immunother 2013;62:737–746.
[13] Heine A, Schilling J, Grunwald B, et al. The induction of human myeloid derived suppressor cells through hepatic stellate cells is dose-dependently inhibited by the tyrosine kinase inhibitors nilotinib, dasatinib and sorafenib, but not sunitinib. Cancer Immunol Immunother 2016;65:273–282.
[14] Kalathil SG, Hutson A, Barbi J, et al. Augmentation of IFN-gamma+ CD8+ T cell responses correlates with survival of HCC patients on sorafenib therapy. JCI Insight 2019;4:e130116.
[15] Kalathil SG, Lugade AA, Miller A, et al. PD-1(+) and Foxp3(+) T cell reduction correlates with survival of HCC patients after sorafenib therapy. JCI Insight 2016;1:e86182.
[16] Lu LC, Lee YH, Chang CJ, et al. Increased expression of programmed death-ligand 1 in infiltrating immune cells in hepatocellular carcinoma tissues after sorafenib treatment. Liver Cancer 2019;8:110–120.
[17] Yakes FM, Chen J, Tan J, et al. Cabozantinib (XL184), a novel MET and VEGFR2 inhibitor, simultaneously suppresses metastasis, angiogenesis, and tumor growth. Mol Cancer Ther 2011;10:2298–2308.
[18] Llovet JM, Pinyol R, Kelley RK, et al. Molecular pathogenesis and systemic therapies for hepatocellular carcinoma. Nat Cancer 2022;3:386–401.
[19] Huang Y, Yuan J, Righi E, et al. Vascular normalizing doses of anti-angiogenic treatment reprogram the immunosuppressive tumor microenvironment and enhance immunotherapy. Proc Natl Acad Sci U S A 2012;109:17561–17566.
[20] Finn RS, Qin S, Ikeda M, et al. Atezolizumab plus bevacizumab in unresectable hepatocellular carcinoma. New Engl J Med 2020;382:1894–1905.
[21] Qin S, Chan SL, Gu S, et al. Camrelizumab plus rivoceranib *vs.* sorafenib as first-line therapy for unresectable hepatocellular carcinoma (CARES-310): a randomised, open-label, international phase 3 study. Lancet 2023;402:1133–1146.
[22] Kelley RK, Rimassa L, Cheng AL, et al. Cabozantinib plus atezolizumab *vs.* sorafenib for advanced hepatocellular carcinoma (COSMIC-312): a multicentre, open-label, randomised, phase 3 trial. Lancet Oncol 2022;23:995–1008.
[23] Cheng AL, Qin S, Ikeda M, et al. Updated efficacy and safety data from IMbrave150: atezolizumab plus bevacizumab vs. sorafenib for unresectable hepatocellular carcinoma. J Hepatol 2022;76:862–873.
[24] Aly A, Fulcher N, Seal B, et al. Clinical outcomes by Child-Pugh Class in patients with advanced hepatocellular carcinoma in a community oncology setting. Hepat Oncol 2023;10:HEP47.
[25] Costa F, Wiedenmann B, Roderburg C, et al. Systemic treatment in patients with Child-Pugh B liver dysfunction and advanced hepatocellular carcinoma. Cancer Med 2023;12:13978–13990.
[26] Kambhampati S, Bauer KE, Bracci PM, et al. Nivolumab in patients with advanced hepatocellular carcinoma and Child-Pugh class B cirrhosis: safety and clinical outcomes in a retrospective case series. Cancer 2019;125:3234–3241.
[27] Wong JSL, Kwok GW, Tang V, et al. Nivolumab/pembrolizumab in Child-Pugh grade B/C patients with advanced HCC. J Clin Oncol 2021;39:e16184.
[28] Xie E, Yeo YH, Scheiner B, et al. Immune checkpoint inhibitors for Child-Pugh Class B advanced hepatocellular carcinoma: a systematic review and meta-analysis. JAMA Oncol 2023;9:1423–1431.
[29] Chapin WJ, Hwang WT, Karasic TB, et al. Comparison of nivolumab and sorafenib for first systemic therapy in patients with hepatocellular carcinoma and Child-Pugh B cirrhosis. Cancer Med 2023;12:189–199.
[30] Kelley RK, Miksad R, Cicin I, et al. Efficacy and safety of cabozantinib for patients with advanced hepatocellular carcinoma based on albumin-bilirubin grade. Br J Cancer 2022;126:569–575.
[31] Kudo M, Finn RS, Cheng AL, et al. Albumin-bilirubin grade analyses of atezolizumab plus bevacizumab *vs.* sorafenib in patients with unresectable hepatocellular carcinoma: a post hoc analysis of the Phase III IMbrave150 Study. Liver Cancer 2023;12:479–493.
[32] Pinato DJ, Kaneko T, Saeed A, et al. Immunotherapy in hepatocellular cancer patients with mild to severe liver dysfunction: adjunctive role of the ALBI grade. Cancers 2020;12:1862.
[33] Kudo M, Matilla A, Santoro A, et al. CheckMate 040 cohort 5: a phase I/II study of nivolumab in patients with advanced hepatocellular carcinoma and Child-Pugh B cirrhosis. J Hepatol 2021;75:600–609.
[34] D'Alessio A, Fulgenzi CAM, Nishida N, et al. Preliminary evidence of safety and tolerability of atezolizumab plus bevacizumab in patients with hepatocellular carcinoma and Child-Pugh A and B cirrhosis: a real-world study. Hepatology 2022;76:1000–1012.
[35] Marrero JA, Kudo M, Venook AP, et al. Observational registry of sorafenib use in clinical practice across Child-Pugh subgroups: the GIDEON study. J Hepatol 2016;65:1140–1147.
[36] Abou-Alfa GK, Amadori D, Santoro A, et al. Safety and efficacy of sorafenib in patients with hepatocellular carcinoma (HCC) and Child-Pugh A versus B cirrhosis. Gastrointest Cancer Res 2011;4:40–44.
[37] Reig M, Forner A, Rimola J, et al. BCLC strategy for prognosis prediction and treatment recommendation: the 2022 update. J Hepatol 2022;76:681–693.
[38] Hartoularos GC, Si Y, Zhang F, et al. Reference-free multiplexed single-cell sequencing identifies genetic modifiers of the human immune response. bioRxiv 2023. https://doi.org/10.1101/2023.05.29.542756. Published online June 3.
[39] Wolock SL, Lopez R, Klein AM. Scrublet: computational identification of cell doublets in single-cell transcriptomic data. Cell Syst 2019;8:281–291.
[40] Wolf FA, Angerer P, Theis FJ. SCANPY: large-scale single-cell gene expression data analysis. Genome Biol 2018;19:15.
[41] Johnson WE, Li C, Rabinovic A. Adjusting batch effects in microarray expression data using empirical Bayes methods. Biostatistics 2007;8:118–127.
[42] Kim MC, Gate R, Lee DS, et al. Method of moments framework for differential expression analysis of single-cell RNA sequencing data. Cell 2024;187:6393–6410.
[43] Fang Z, Liu X, Peltz G. GSEApy: a comprehensive package for performing gene set enrichment analysis in Python. Bioinformatics 2023;39. btac757.
[44] **Lyu A**, **Fan Z**, Clark M, et al. Evolution of myeloid-mediated immunotherapy resistance in prostate cancer. Nature 2025;637:1207–1217.
[45] Hochberg Y, Benjamini Y. More powerful procedures for multiple significance testing. Stat Med 1990;9:811–818.
[46] Keenan BP, McCarthy EE, Ilano A, et al. Circulating monocytes associated with anti-PD-1 resistance in human biliary cancer induce T cell paralysis. Cell Rep 2022;40:111384.
[47] Huang AC, Postow MA, Orlowski RJ, et al. T-cell invigoration to tumour burden ratio associated with anti-PD-1 response. Nature 2017;545:60–65.
[48] Johnson PJ, Berhane S, Kagebayashi C, et al. Assessment of liver function in patients with hepatocellular carcinoma: a new evidence-based approach-the ALBI grade. J Clin Oncol 2015;33:550–558.
[49] Xing P, Zhang F, Wang G, et al. Incidence rates of immune-related adverse events and their correlation with response in advanced solid tumours treated with NIVO or NIVO+IPI: a systematic review and meta-analysis. J Immunother Cancer 2019;7:341.
[50] Cook S, Samuel V, Meyers DE, et al. Immune-related adverse events and survival among patients with metastatic NSCLC treated with immune checkpoint inhibitors. JAMA Netw Open 2024;7:e2352302.
[51] Llovet JM, Kudo M, Merle P, et al. Lenvatinib plus pembrolizumab *vs.* lenvatinib plus placebo for advanced hepatocellular carcinoma (LEAP-002): a randomised, double-blind, phase 3 trial. Lancet Oncol 2023;24:1399–1410.
[52] Yau T, Galle PR, Decaens T, et al. Nivolumab plus ipilimumab *vs.* lenvatinib or sorafenib as first-line treatment for unresectable hepatocellular carcinoma (CheckMate 9DW): an open-label, randomised, phase 3 trial. Lancet 2025;405:1851–1864.
[53] Mok K, Chen O, Yau J, et al. Systemic therapy for Child-Pugh B patients with hepatocellular carcinoma. Expert Opin Emerg Drugs 2025;405:1851–1864.

[54] Zhu AX, Baron AD, Malfertheiner P, et al. Ramucirumab as second-line treatment in patients with advanced hepatocellular carcinoma: analysis of REACH trial results by Child-Pugh Score. JAMA Oncol 2017;3:235–243.
[55] Fulgenzi CAM, Scheiner B, D'Alessio A, et al. Immunotherapy versus best supportive care for patients with hepatocellular cancer with Child-Pugh B dysfunction. JAMA Oncol 2024;10:1253–1258.
[56] El Hajra I, Sanduzzi-Zamparelli M, Sapena V, et al. Outcome of patients with HCC and liver dysfunction under immunotherapy: a systematic review and meta-analysis. Hepatology 2023;77:1139–1149.
[57] Sarker D, Plummer R, Meyer T, et al. MTL-CEBPA, a small activating RNA therapeutic upregulating C/EBP-alpha, in patients with advanced liver cancer: a first-in-human, multicenter, open-label, Phase I trial. Clin Cancer Res 2020;26:3936–3946.
[58] Pfister D, Nunez NG, Pinyol R, et al. NASH limits anti-tumour surveillance in immunotherapy-treated HCC. Nature 2021;592:450–456.
[59] Ho WJ, Danilova L, Lim SJ, et al. Viral status, immune microenvironment and immunological response to checkpoint inhibitors in hepatocellular carcinoma. J Immunother Cancer 2020;8:e000394.
[60] Kelley RK, Greten TF. Hepatocellular carcinoma - origins and outcomes. New Engl J Med 2021;385:280–282.
[61] Kulik L, El-Serag HB. Epidemiology and management of hepatocellular carcinoma. Gastroenterology 2019;156:477–491.
[62] Shi L, Lu J, Zhong D, et al. Clinicopathological and predictive value of MAIT cells in non-small cell lung cancer for immunotherapy. J ImmunoTherapy Cancer 2023;11:e005902.
[63] Guo X, Nie H, Zhang W, et al. Contrasting cytotoxic and regulatory T cell responses underlying distinct clinical outcomes to anti-PD-1 plus lenvatinib therapy in cancer. Cancer Cell 2025;43:248–268.
[64] Soto M, Filbert EL, Yang H, et al. Neoadjuvant CD40 agonism remodels the tumor immune microenvironment in locally advanced esophageal/gastroesophageal junction cancer. Cancer Res Commun 2024;4:200–212.
[65] Rodriguez-Prados JC, Traves PG, Cuenca J, et al. Substrate fate in activated macrophages: a comparison between innate, classic, and alternative activation. J Immunol 2010;185:605–614.
[66] Sasson SC, Zaunders JJ, Nahar K, et al. Mucosal-associated invariant T (MAIT) cells are activated in the gastrointestinal tissue of patients with combination ipilimumab and nivolumab therapy-related colitis in a pathology distinct from ulcerative colitis. Clin Exp Immunol 2020;202:335–352.
[67] Haga K, Chiba A, Shibuya T, et al. MAIT cells are activated and accumulated in the inflamed mucosa of ulcerative colitis. J Gastroenterol Hepatol 2016;31:965–972.
[68] Liu Y, Xun Z, Ma K, et al. Identification of a tumour immune barrier in the HCC microenvironment that determines the efficacy of immunotherapy. J/hepatology 2023;78:770–782.
[69] Zhang L, Li Z, Skrzypczynska KM, et al. Single-cell analyses inform mechanisms of myeloid-targeted therapies in colon cancer. Cell 2020;181:442–459.
[70] Sulpice L, Rayar M, Desille M, et al. Molecular profiling of stroma identifies osteopontin as an independent predictor of poor prognosis in intrahepatic cholangiocarcinoma. Hepatology 2013;58:1992–2000.

Keywords: Immunotherapy; Hepatocellular carcinoma; Child-Pugh score; Monocytes; Cirrhosis.
Received 3 August 2025; received in revised form 10 February 2026; accepted 3 March 2026; Available online 12 March 2026

Journal of Hepatology, Volume *8*

Supplemental information

Phase II trial with nivolumab and sorafenib in HCC identified enrichment of immunosuppressive monocytes in patients with Child-Pugh B liver dysfunction

Bridget P. Keenan, Zenghua Fan, Bryan Khuong Le, Quincy Harris, Jocelin Chen, Matthew Clark, Averey Lea, Li Zhang, Alexander Cheung, Frances Lara, John D. Gordan, Paige Bracci, Spencer C. Behr, Lawrence Fong, Alan P. Venook, Edward J. Kim, and Robin K. Kelley

Phase II trial with nivolumab and sorafenib in HCC identified enrichment of immunosuppressive monocytes in patients with Child-Pugh B liver dysfunction

Bridget P. Keenan, Zenghua Fan, Bryan Khuong Le, Quincy Harris, Jocelin Chen, Matthew Clark, Averey Lea, Li Zhang, Alexander Cheung, Frances Lara, John D. Gordan, Paige Bracci, Spencer C. Behr, Lawrence Fong, Alan P. Venook, Edward J. Kim[5], Robin K. Kelley

Table of contents

Fig. S1……………………………………………………………………………………2

Fig. S2……………………………………………………………………………………3

Fig. S3……………………………………………………………………………………5

Fig. S4……………………………………………………………………………………6

Fig. S5……………………………………………………………………………………8

Table S1…………………………………………………………………………………..9

Table S2…………………………………………………………………………………..10

Table S3…………………………………………………………………………………..11

Table S4…………………………………………………………………………………..13

Table S5…………………………………………………………………………………..15

Table S6…………………………………………………………………………………..16

Table S7…………………………………………………………………………………..17

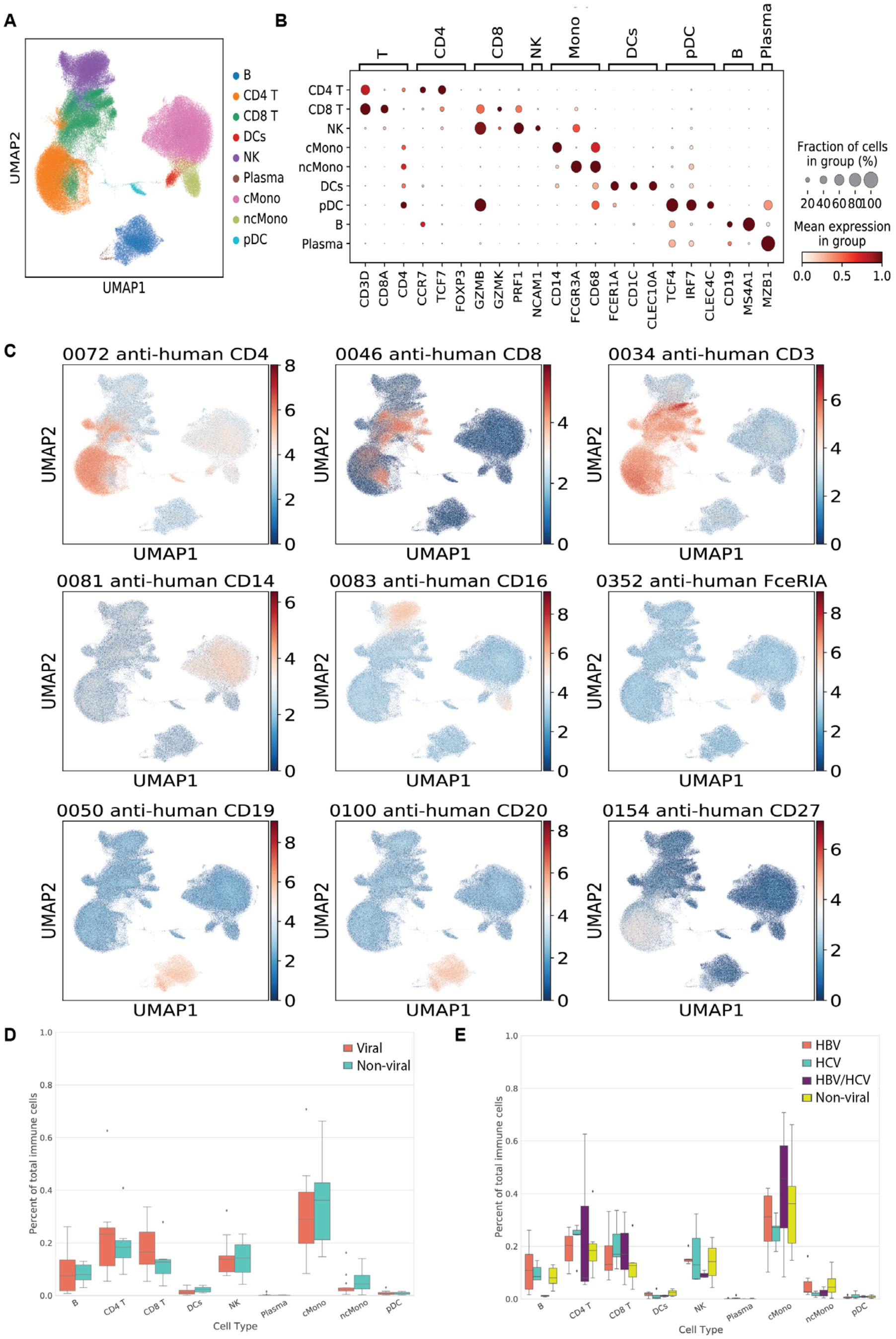

Fig. S1. Characterization of circulating immune cells from patients with advanced HCC with CITEseq. A. Uniform Manifold Approximation and Projection (UMAP) plot of single cell RNA sequencing results from PBMCs colored by immune cell type. **B.** Dotplot demonstrating expression level and the fraction of cells within each immune cell population (y-axis) expressing the RNA molecules indicated. The groupings on the x-axis correspond to the category of markers used to define populations. **C.** UMAP colored by protein expression as indicated by the CITEseq antibody at the top of each plot. **D-E.** Frequency of immune cell type at baseline (pre) for all patients (n=20) by viral status (viral (n=14) or non-viral (n=6) (**D**) and specific viral associations (HBV: n=6, dual HBV/HCV: n=3, HCV: n=5) (**E**)). Boxes denote inter-quartile range (IQR) while bars denote 25% - 1.5xIQR and 75% + 1.5xIQR.

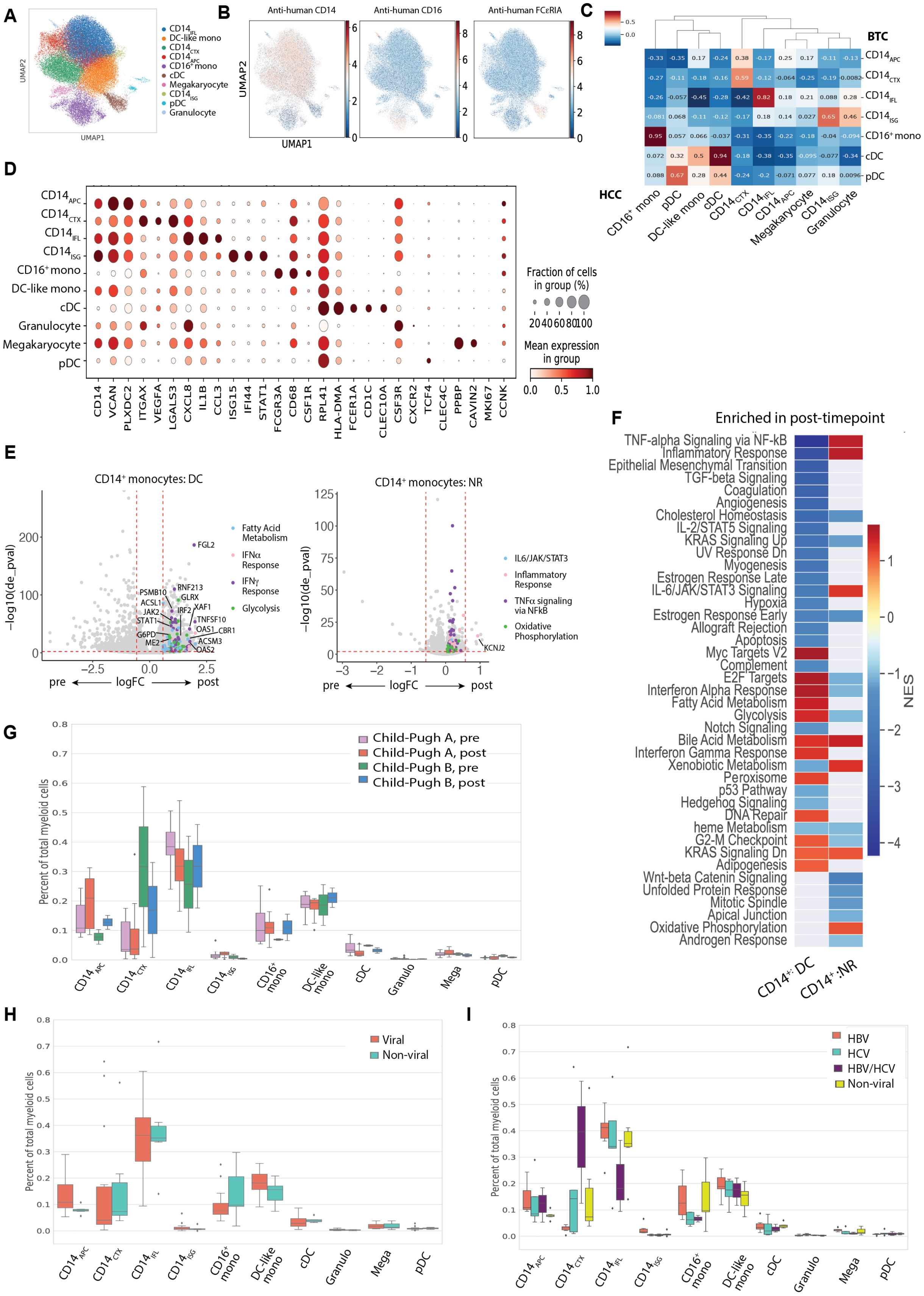

A
CD14IFL
DC-like mono
CD14CTX
CD14APC
CD16+ mono
cDC
Megakaryocyte
CD14ISG
pDC
Granulocyte
UMAP1
UMAP2
B
Anti-human CD14
Anti-human CD16
Anti-human FCεRIA
C
BTC
HCC
CD16+ mono
pDC
DC-like mono
cDC
CD14CTX
CD14IFL
CD14APC
Megakaryocyte
CD14ISG
Granulocyte
D
Fraction of cells in group (%)
20 40 60 80 100
Mean expression in group
0.0 0.5 1.0
CD14 VCAN PLXDC2 ITGAX VEGFA LGALS3 CXCL8 IL1B CCL3 ISG15 IFI44 STAT1 FCGR3A CD68 CSF1R RPL41 HLA-DMA FCER1A CD1C CLEC10A CSF3R CXCR2 TCF4 CLEC4C PPBP CAVIN2 MKI67 CCNK
E
CD14+ monocytes: DC
CD14+ monocytes: NR
-log10(de_pval)
pre ← logFC → post
Fatty Acid Metabolism
IFNα Response
IFNγ Response
Glycolysis
IL6/JAK/STAT3
Inflammatory Response
TNFα signaling via NFkB
Oxidative Phosphorylation
FGL2
RNF213
PSMB10
GLRX
ACSL1
IRF2
XAF1
JAK2
STAT1
TNFSF10
OAS1
CBR1
G6PD
ME2
ACSM3
OAS2
KCNJ2
F
Enriched in post-timepoint
TNF-alpha Signaling via NF-kB
Inflammatory Response
Epithelial Mesenchymal Transition
TGF-beta Signaling
Coagulation
Angiogenesis
Cholesterol Homeostasis
IL-2/STAT5 Signaling
KRAS Signaling Up
UV Response Dn
Myogenesis
Estrogen Response Late
IL-6/JAK/STAT3 Signaling
Hypoxia
Estrogen Response Early
Allograft Rejection
Apoptosis
Myc Targets V2
Complement
E2F Targets
Interferon Alpha Response
Fatty Acid Metabolism
Glycolysis
Notch Signaling
Bile Acid Metabolism
Interferon Gamma Response
Xenobiotic Metabolism
Peroxisome
p53 Pathway
Hedgehog Signaling
DNA Repair
heme Metabolism
G2-M Checkpoint
KRAS Signaling Dn
Adipogenesis
Wnt-beta Catenin Signaling
Unfolded Protein Response
Mitotic Spindle
Apical Junction
Oxidative Phosphorylation
Androgen Response
NES
CD14+: DC
CD14+: NR
G
Child-Pugh A, pre
Child-Pugh A, post
Child-Pugh B, pre
Child-Pugh B, post
Percent of total myeloid cells
CD14APC
CD14CTX
CD14IFL
CD14ISG
CD16+ mono
DC-like mono
cDC
Granulo
Mega
pDC
H
Viral
Non-viral
Percent of total myeloid cells
I
HBV
HCV
HBV/HCV
Non-viral
Percent of total myeloid cells

Fig. S2. Characterization of circulating myeloid cells from patients with advanced HCC and association with clinical response and liver disease characteristics. A. Uniform Manifold Approximation and Projection (UMAP) plot of single cell RNA sequencing results from PBMCs colored by myeloid cell sub-type. **B.** UMAP colored by protein expression as indicated by the CITEseq antibody at the top of each plot. **C.** Comparison of gene signatures for each myeloid cell type in the HCC dataset (x-axis) with myeloid cells from the BTC dataset (y-axis) using pseudobulk gene expression. Legend shows correlation values demonstrated in heatmap format. **D.** Dotplot demonstrating expression level and the fraction of cells within each myeloid cell population (y-axis) expressing the RNA molecules indicated. **E.** Differentially expressed genes in post-treatment vs pre-treatment timepoints for all $CD14^{+}$ monocytes in the DC and NR groups are plotted, with genes of interest in the indicated pathways highlighted with the corresponding color dot. **F.** Pathways enriched within all $CD14^{+}$ monocytes ($CD14_{ISG}$, $CD14_{CTX}$, $CD14_{APC}$, $CD14_{IFL}$, and DC-like monocytes) (x-axis) in the post-treatment compared to pre-treatment timepoint are shown on the y-axis; heatmap corresponds to normalized enrichment score (NES). Columns correspond to the disease control (DC) or non-responder (NR) groups. **G.** Percent of each cell type out of total myeloid cells in circulation for patients with Child-Pugh A (n=10) or Child-Pugh B (n=4) prior to treatment (pre) and following one cycle of combination treatment (post). **H-I.** Frequency of myeloid cell type out of total circulating myeloid cells at baseline (pre) for all patients (n=20) by viral status (viral (n=14) or non-viral (n=6)) (**B**) and specific viral associations (HBV: n=6, dual HBV/HCV: n=3, HCV: n=5) (**C**). Boxes denote inter-quartile range (IQR) while bars denote 25% - 1.5xIQR and 75% + 1.5xIQR.

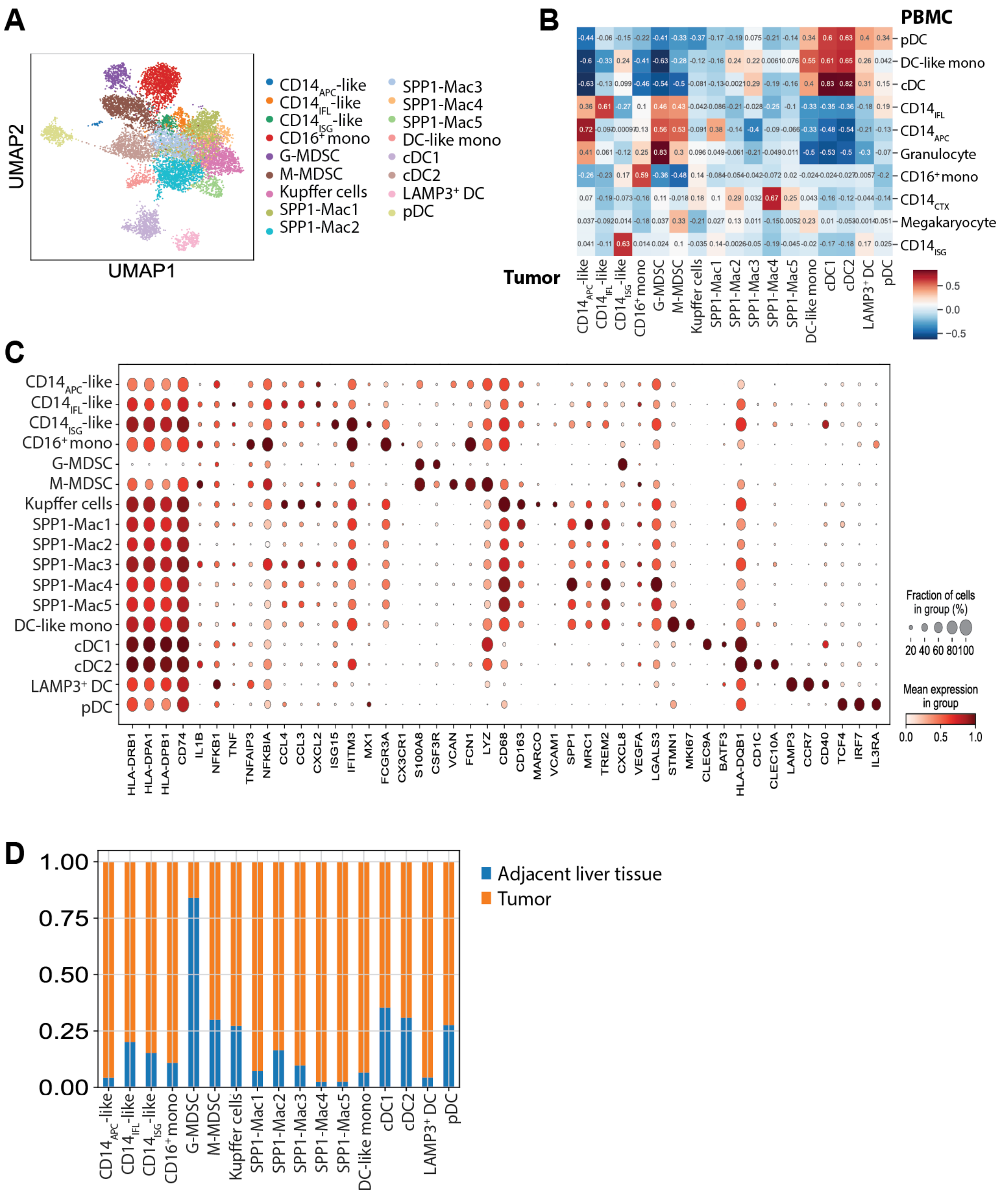

Fig. S3. Intra-tumoral myeloid cells from HCC tumor tissues and comparison to circulating myeloid cell populations. A. UMAP plot of single cell RNA sequencing results from tumor and adjacent liver tissue colored by myeloid cell sub-type. (MDSC = myeloid-derived suppressor cell, Mac = macrophage). **B.** Comparison of gene signatures for each myeloid cell type in the HCC tumor dataset (x-axis) with myeloid cells from the HCC PBMC dataset (y-axis) using pseudobulk gene expression. Legend shows correlation values demonstrated in heatmap format. **C.** Dotplot demonstrating expression level and the fraction of cells within each myeloid cell population (y-axis) expressing the RNA molecules indicated. **D.** Barplot showing the fraction of each population made up by the cell type from either the tumor specimens or adjacent liver tissue.

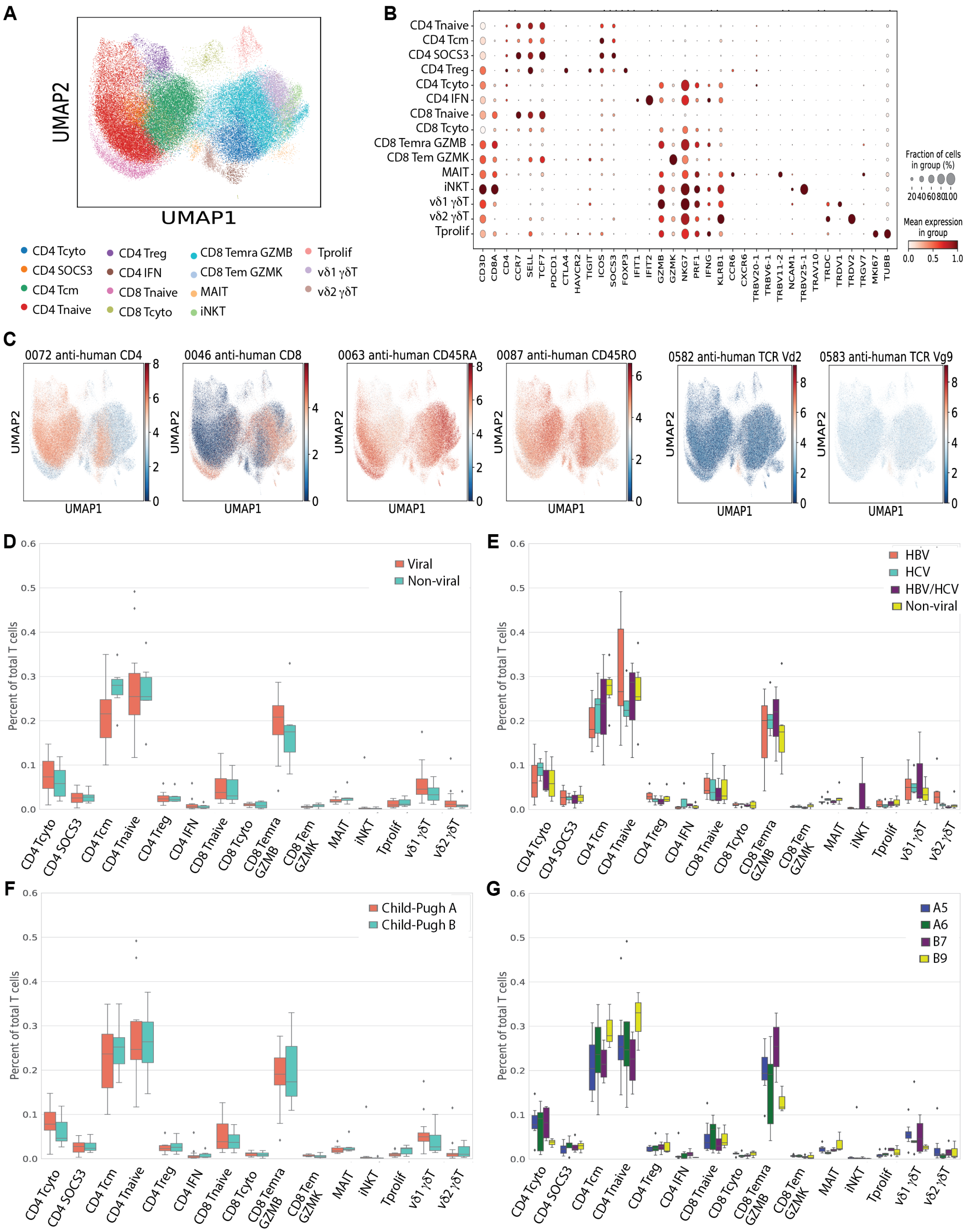

Fig. S4. Characterization of circulating T cells from patients with advanced HCC with CITEseq. A. Uniform Manifold Approximation and Projection (UMAP) plot of single cell RNA sequencing results from PBMCs colored by T cell sub-type. **B.** Dotplot demonstrating expression level and the fraction of cells within each T cell population (y-axis) expressing the RNA molecules indicated. The groupings on the x-axis correspond to the category of markers

used to define populations. **C.** UMAP colored by protein expression as indicated by the CITEseq antibody at the top of each plot. **D-G.** Frequency of T cell type out of total circulating T cells at baseline (pre) for all patients (n=20) by viral status (viral (n=14) or non-viral (n=6)) (**D**), specific viral associations (HBV: n=6, dual HBV/HCV: n=3, HCV: n=5) (**E**)) and by Child-Pugh status (A (n=13) vs B (n=7) (**F**), Child-Pugh sub-score (A5: n=8, A6: n= 5, B7: n=4, B9: n=3) (**G**)). Boxes denote inter-quartile range (IQR) while bars denote 25% - 1.5xIQR and 75% + 1.5xIQR.

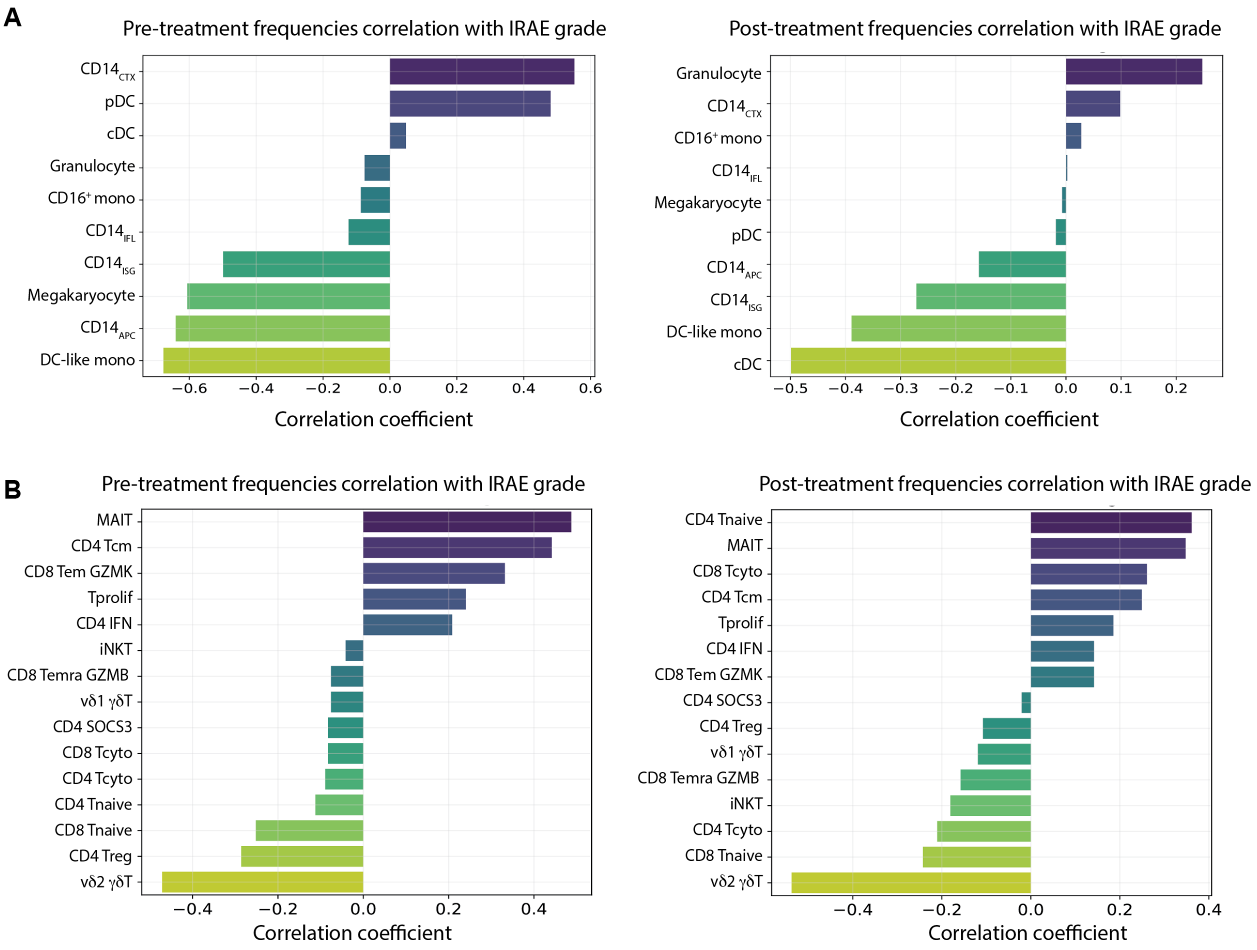

Fig. S5. Association of circulating myeloid and T cells with immune-related adverse events (IRAEs). A. Correlation coefficients for the frequency of each myeloid cell sub-type at the pre- and post-treatment timepoints and grade of IRAE in patients that experienced IRAEs. **B.** Correlation coefficients for the frequency of each T cell sub-type at the pre- and post-treatment timepoints and grade of IRAE in patients that experienced IRAEs.

Table S1. Demographic characteristics of patients with collected PBMCs for analysis.

**Demographic**		
		**Screening only cohort**
		**n=5**
Median age, years (SD)		65 (5.3)
Male, n (%)		4 (80)
Race, n (%)	White	4 (80)
	Black or African American	0
	Asian	1 (20)
	American Indian or Alaska Native	0
Ethnicity	Non-Hispanic/Latinx	4 (80)
	Hispanic/Latinx	1 (20)
Viral status	HBV+*	1 (20)
	HCV+*	2 (40)
	Non-viral	2 (40)
Child-Pugh score at	A5	2 (40)
screening	A6	1 (20)
	B7	1 (20)
	B9	1 (20)
ALBI grade at enrollment	1	0
	2	2 (40)
	3	3 (60)

Patients included in the table were screened and not enrolled in the Phase II clinical trial of combination sorafenib and nivolumab.

*Some patients had both HBVcAb+ and HCV antibody positive, but were HBsAg negative

Table S2. Clinical characteristics of separate HCC tumor resection cohort.

Patient ID	Viral Status	Age at collection	Race	Ethnicity	TNM staging	BCLC stage	Child-Pugh stage
A-12	non-viral	64	White	Non-Hispanic/Latinx	IA	0	A
A-18	HBV+HCV+	58	Asian	Non-Hispanic/Latinx	IB	A	A
A-19	HBV+	66	Asian	Non-Hispanic/Latinx	II	A	A
A-20	HBV+	58	Asian	Non-Hispanic/Latinx	IA	0	A
A-23	non-viral	81	White	Non-Hispanic/Latinx	IIIA	A	A
A-24	HBV+	64	Asian	Non-Hispanic/Latinx	II	A	A
A-30	non-viral	67	White	Non-Hispanic/Latinx	IIIA	A	A

Table S3. Treatment-related adverse event (TRAE) occurring in more than one patient by grade and dose level.

**Dose Level -1 (n=11)**			
**Common TRAEs (n>1 in total cohort)**			
**AE**	**Grade 1/2**	**Grade 3/4**	**Any grade**
Rash	7 (63.6)	1 (9.1)	8 (72.7)
Hypertension	5 (45.5)	1 (9.1)	6 (54.5)
Increased aspartate aminotransferase	2 (18.2)	3 (27.3)	5 (45.5)
Diarrhea	4 (36.4)	0	4 (36.4)
Decreased appetite/anorexia	3 (27.3)	0	3 (27.3)
Emesis	3 (27.3)	0	3 (27.3)
Hoarseness	3 (27.3)	0	3 (27.3)
Increased alanine aminotransferase	3 (27.3)	0	3 (27.3)
Myalgia	3 (27.3)	0	3 (27.3)
Mucositis	2 (18.2)	1 (9.1)	3 (27.3)
Nausea	3 (27.3)	0	3 (27.3)
Palmar-plantar erythrodysesthesia syndrome	2 (18.2)	1 (9.1)	3 (27.3)
Pruritus	3 (27.3)	0	3 (27.3)
Dry skin	2 (18.2)	0	2 (18.2)
Fatigue	2 (18.2)	0	2 (18.2)
Weight loss	2 (18.2)	0	2 (18.2)
Constipation	1 (9.1)	0	1 (9.1)
Headache	1 (9.1)	0	1 (9.1)
Hyperbilirubinemia	0	1 (9.1)	1 (9.1)
Hypoalbuminemia	1 (9.1)	0	1 (9.1)
Dry eye	0	0	0
Dyspepsia	0	0	0
Hyponatremia	0	0	0

**Dose Level 1 (n=5)**			
**Common TRAEs (n>1 in total cohort)**			
**AE**	**Grade 1/2**	**Grade 3/4**	**Any grade**
Rash	3 (60)	0	3 (60)
Constipation	2 (40)	0	2 (40)
Diarrhea	2 (40)	0	2 (40)
Dry eye	2 (40)	0	2 (40)
Dry skin	2 (40)	0	2 (40)

Dyspepsia	2 (40)	0	2 (40)
Hyperbilirubinemia	0	2 (40)	2 (40)
Hyponatremia	1 (20)	1 (20)	2 (40)
Increased alanine aminotransferase	2 (40)	0	2 (40)
Increased aspartate aminotransferase	2 (40)	0	2 (40)
Myalgia	2 (40)	0	2 (40)
Palmar-plantar erythrodysesthesia syndrome	0	2 (40)	2 (40)
Headache	1 (20)	0	1 (20%)
Hoarseness	1 (20)	0	1 (20%)
Hypoalbuminemia	1 (20)	0	1 (20%)
Fatigue	0	1 (20%)	1 (20%)
Mucositis	1 (20%)	0	1 (20%)
Decreased appetite/anorexia	0	0	0
Emesis	0	0	0
Hypertension	0	0	0
Nausea	0	0	0
Pruritus	0	0	0
Weight loss	0	0	0

Table S4. Treatment-related adverse event (TRAE) occurring in more than one patient by grade and Child-Pugh status.

**Child Pugh A (n=10)**			
**Common TRAEs (n>1 in total cohort)**			
**AE**	**Grade 1/2**	**Grade 3/4**	**Any grade**
Rash	7 (70)	1 (10)	8 (80)
Increased alanine aminotransferase	4 (40)	0	4 (40)
Increased aspartate aminotransferase	4 (40)	0	4 (40)
Palmar-plantar erythrodysesthesia syndrome	1 (10)	3 (30)	4 (40)
Decreased appetite/anorexia	3 (30)	0	3 (30)
Diarrhea	3 (30)	0	3 (30)
Fatigue	2 (20)	1 (10)	3 (30)
Hoarseness	3 (30)	0	3 (30)
Hypertension	2 (20)	1 (10)	3 (30)
Mucositis	3 (30)	0	3 (30)
Myalgia	3 (30)	0	3 (30)
Constipation	2 (20)	0	2 (20)
Dry eye	2 (20)	0	2 (20)
Dry skin	2 (20)	0	2 (20)
Dyspepsia	2 (20)	0	2 (20)
Emesis	2 (20)	0	2 (20)
Headache	2 (20)	0	2 (20)
Hypoalbuminemia	2 (20)	0	2 (20)
Hyponatremia	1 (10)	1 (10)	2 (20)
Nausea	2 (20)	0	2 (20)
Pruritus	2 (20)	0	2 (20)
Weight loss	2 (20)	0	2 (20)
Hyperbilirubinemia	0	1 (10)	1 (10)
**Other Gr 3/4 TRAEs (n=1 in total cohort)**			
Keratoacanthoma/florid lichenoid skin reaction		1 (10)	
**Individual patients with Gr3/4 TRAE**		**7 (70)**	
**Child Pugh B (n=6)**			
**Common TRAEs (n>1 in total cohort)**			
**AE**	**Grade 1/2**	**Grade 3/4**	**Any grade**
Diarrhea	3 (50)	0	3 (50)
Hypertension	3 (50)	0	3 (50)

Increased aspartate aminotransferase	0	3 (50)	3 (50)
Rash	3 (50)	0	3 (50)
Dry skin	2 (33.3)	0	2 (33.3)
Hyperbilirubinemia	0	2 (33.3)	2 (33.3)
Myalgia	2 (33.3)	0	2 (33.3)
Constipation	1 (16.7)	0	1 (16.7)
Emesis	1 (16.7)	0	1 (16.7)
Hoarseness	1 (16.7)	0	1 (16.7)
Increased alanine aminotransferase	1 (16.7)	0	1 (16.7)
Mucositis	0	1 (16.7)	1 (16.7)
Nausea	1 (16.7)	0	1 (16.7)
Palmar-plantar erythrodysesthesia syndrome	1 (16.7)	0	1 (16.7)
Pruritus	1 (16.7)	0	1 (16.7)
Decreased appetite/anorexia	0	0	0
Dry eye	0	0	0
Dyspepsia	0	0	0
Headache	0	0	0
Hypoalbuminemia	0	0	0
Hyponatremia	0	0	0
Fatigue	0	0	0
Weight loss	0	0	0
**Other Gr 3/4 TRAEs (n=1 in total cohort)**			
Immune related hepatitis		1 (16.7)	
**Individual patients with Gr3/4 TRAE**		**4 (66.7)**	

Table S5. Immune-related adverse event (IRAE) occurring in more than one patient by grade.

Immune-related adverse event occurring in n>1 patient, n (%)	Grade 1 or 2	Grade 3 or 4	Any grade
Rash	9 (56.3)	0	9 (56.3)
Increased aspartate aminotransferase	3 (18.8)	2 (12.5)	5 (31.3)
Diarrhea	3 (18.8)	0	3 (18.8)
Fatigue	2 (12.5)	1 (6.25)	3 (18.8)
Mucositis	2 (12.5)	1 (6.25)	3 (18.8)
Myalgia	3 (18.8)	0	3 (18.8)
Constipation	2 (12.5)	0	2 (12.5)
Decreased appetite	2 (12.5)	0	2 (12.5)
Dry eye	2 (12.5)	0	2 (12.5)
Dry skin	2 (12.5)	0	2 (12.5)
Emesis	2 (12.5)	0	2 (12.5)
Hyperbilirubinemia	1 (6.25)	1 (6.25)	2 (12.5)
Hyponatremia	1 (6.25)	1 (6.25)	2 (12.5)
Increased alanine aminotransferase	2 (12.5)	0	2 (12.5)
Nausea	2 (12.5)	0	2 (12.5)

Table S6. Immune-related adverse event (IRAE) occurring in more than one patient by grade and Child-Pugh status.

Child Pugh A (n=10)				Child Pugh B (n=6)		
**Common IRAEs (n>1 in total cohort)**						
**Immune-related adverse event, n (%)**	**Grade 1 or 2**	**Grade 3 or 4**	**Any grade**	**Grade 1 or 2**	**Grade 3 or 4**	**Any grade**
Rash	6 (60)	0	6 (60)	3 (50)	0	3 (50)
Increased aspartate aminotransferase	3 (30)	0	3 (30)	0	2 (33.3)	2 (33.3)
Diarrhea	2 (20)	0	2 (20)	1 (16.7)	0	1 (16.7)
Fatigue	2 (20)	1 (10)	3 (30)	0	0	0
Myalgia	2 (20)	0	2 (20)	1 (16.7)	0	1 (16.7)
Mucositis	2 (20)	0	2 (20)	0	1 (16.7)	1 (16.7)
Constipation	1 (10)	0	1 (10)	1 (16.7)	0	1 (16.7)
Decreased appetite	2 (20)	0	2 (20)	0	0	0
Dry eye	2 (20)	0	2 (20)	0	0	0
Dry skin	0	0	0	2 (33.3)	0	2 (33.3)
Emesis	1 (10)	0	1 (10)	1 (16.7)	0	1 (16.7)
Hyperbilirubinemia	1 (10)	0	1 (10)	0	1 (16.7)	1 (16.7)
Hyponatremia	1 (10)	1 (10)	2 (20)	0	0	0
Increased alanine aminotransferase	1 (10)	0	1 (10)	1 (16.7)	0	1 (16.7)
Nausea	1 (10)	0	1 (10)	1 (16.7)	0	1 (16.7)
**Individual patients with Gr3/4 IRAEs**		2 (20)			3 (50)	

Table S7. Clinical responses overall and by dose level (DL).

	DL –1 (n=11)	DL 1 (n=5)	Total (n=16)
Complete response (CR)	0	0	0
Partial response (PR)	1 (9.1)	0	1 (6.3)
Stable disease (SD)	8 (72.7)	0	8 (50)
Progressive disease (PD)	2 (18.1)	5 (100)	7 (43.8)
Not evaluable (NE)	0	0	0
**Response rate, n (%)**			
Objective response rate (CR/PR)	1 (9.1)	0	1 (6.3)
Disease control rate (CR/PR/SD)	9 (81.8)	0	9 (56.3)
**Kaplan-Meier estimate of median duration of SD, months (95% confidence interval)**			
Progression-free survival (PFS)	3.68 (2.83, NR)	1.84 (1.58, NR)	2.58 (2.01, 7.17)
Overall survival (OS)	15.2 (10.03, NR)	8.78 (5.95, NR)	12.99 (8.65, 35.8)
